# Glial dysregulation in the human brain in fragile X-associated tremor/ataxia syndrome

**DOI:** 10.1073/pnas.2300052120

**Published:** 2023-05-30

**Authors:** Caroline M. Dias, Biju Issac, Liang Sun, Abigail Lukowicz, Maya Talukdar, Shyam K. Akula, Michael B. Miller, Katherine Walsh, Shira Rockowitz, Christopher A. Walsh

**Affiliations:** ^a^Division of Developmental Medicine, Boston Children’s Hospital, Boston, MA 02115; ^b^Division of Genetics and Genomics, Manton Center for Orphan Disease Research, Boston Children’s Hospital, Boston, MA 02115; ^c^Department of Pediatrics, Harvard Medical School, Boston, MA 02115; ^d^Department of Pediatrics, Section of Developmental Pediatrics, Section of Genetics and Metabolism, and Denver Fragile X Clinic and Research Center, Children’s Hospital Colorado, University of Colorado Anschutz Medical Campus, Aurora, CO 80045; ^e^Research Computing, Department of Information Technology, Boston Children’s Hospital, Boston, MA 02115; ^f^Harvard-Massachusetts Institute of Technology MD/PhD Program, Program in Bioinformatics & Integrative Genomics, Harvard Medical School, Boston, MA 02115; ^g^Harvard-Massachusetts Institute of Technology MD/PhD Program, Program in Neuroscience, Harvard Medical School, Boston, MA 02115; ^h^Department of Pathology, Brigham and Women’s Hospital, Boston, MA 02115; ^i^HHMI, Boston Children’s Hospital, Boston, MA 02115; ^j^Department of Neurology, Harvard Medical School, Boston, MA 02115

**Keywords:** FMR1, FXTAS, human brain, glia, snRNA-seq

## Abstract

Short trinucleotide expansions at the FMR1 locus are associated with the late-onset condition fragile X-associated tremor/ataxia syndrome (FXTAS), which shows very different clinical and pathological features from fragile X syndrome (associated with longer expansions), with no clear molecular explanation for these marked differences. One prevailing theory posits that the shorter, premutation expansion uniquely causes extreme neurotoxic increases in FMR1 mRNA (i.e., four to eightfold increases), but evidence to support this hypothesis is largely derived from analysis of peripheral blood. We applied single-nucleus RNA sequencing to postmortem frontal cortex and cerebellum from 7 individuals with premutation and matched controls (n = 6) to assess cell type–specific molecular neuropathology. We found only modest upregulation (~1.3-fold) of FMR1 in some glial populations associated with premutation expansions. In premutation cases, we also identified decreased astrocyte proportions in the cortex. Differential expression and gene ontology analysis demonstrated altered neuroregulatory roles of glia. Using network analyses, we identified cell type–specific and region-specific patterns of FMR1 protein target gene dysregulation unique to premutation cases, with notable network dysregulation in the cortical oligodendrocyte lineage. We used pseudotime trajectory analysis to determine how oligodendrocyte development was altered and identified differences in early gene expression in oligodendrocyte trajectories in premutation cases specifically, implicating early cortical glial developmental perturbations. These findings challenge dogma regarding extremely elevated *FMR1* increases in FXTAS and implicate glial dysregulation as a critical facet of premutation pathophysiology, representing potential unique therapeutic targets directly derived from the human condition.

## Significance

Genetic variation at the *FMR1* locus confers risk for both the neurodevelopmental disorder fragile X syndrome and the neurodegenerative condition fragile X-associated tremor/ataxia syndrome. Although animal models have been critical in elucidating molecular mechanisms of cellular dysfunction in fragile X-related disorders, understanding how the human brain is directly impacted remains unresolved. We conducted a cell type–specific transcriptomic analysis of postmortem human brains from individuals with fragile X mutations and matched controls, sequencing over 120,000 nuclei from the frontal cortex and cerebellum. We find evidence for cell type–specific, disease-specific, and regional-specific patterns of transcriptional and FMR1 protein (FMRP) network perturbations, providing a foundation for therapeutic development directly derived from the human condition.

*FMR1-*related disorders contribute to neurologic dysfunction across the lifespan (1, 2). Long trinucleotide (CGG) expansion (i.e., full mutations) in the 5′ UTR of the *FMR1* gene are associated with the neurodevelopmental disorder fragile X syndrome (FXS), while short, “premutations” are associated with fragile X-associated tremor and ataxia syndrome (FXTAS), a late-onset condition characterized by executive functioning decline and progressive cerebellar ataxia, presenting in a subset of premutation carriers (3–7). In the latter, neuropathological and imaging studies have identified intranuclear neuronal and astrocytic inclusions, prominent white matter abnormalities including myelin pallor and spongiosis, and characteristic T2 white matter hyperintensities on MRI (3, 8–11). In contrast, in FXS, an early-onset neurodevelopmental disorder characterized by intellectual disability and characteristic facial features (12, 13), only subtle functional changes in white matter in humans have been identified on imaging (14–16). The molecular correlates of these findings in both conditions are unknown.

The full mutation is associated with hypermethylation and transcriptional silencing of the *FMR1* locus, and absent FMR1 protein (FRMP), while the premutation has been reported to be paradoxically associated with increases in FMR1 mRNA, particularly in blood, with variable reductions in FMRP levels (5–7, 17–21). FMRP is a critical RNA-binding and regulatory protein that acts as a central hub in brain function (22–25). Although individuals with the premutation may present with alterations in typical neurodevelopment, FXS patients generally do not present with features of FXTAS. These divergent clinical and molecular phenotypes have led to the hypothesis that the clinical symptomatology associated with FXTAS is related to a neurotoxic effect of increased levels of FMR1 mRNA in the nervous system. This argument is bolstered by findings of a four to eightfold increase of FMR1 mRNA in peripheral blood cells of individuals with the premutation (19). However, prior bulk studies of human postmortem brain tissue from individuals with the premutation have revealed more modest, ~0.9- to 1.5-fold, changes in FMR1 mRNA (9, 26).

Prior studies in postmortem human brain in both FXS and FXTAS have focused on bulk cellular analysis, which does not resolve cell type–specific molecular alterations. Whereas it is possible that the cellular heterogeneity of the human CNS may mask toxic levels of FMR1 mRNA, other hypotheses, including an inappropriate DNA damage response, mitochondrial stress, and polyglycine-containing peptide accumulation, have been put forth as additional hypotheses to explain the pathophysiology of FXTAS (11, 27, 28). It is also possible that reduced FMRP contributes to premutation pathology in a developmentally distinct manner from the total loss that occurs in FXS. Finally, while studies of the impact of *FMR1* disruption have been focused on postmitotic neurons, there is increasing evidence implicating important roles for *FMR1* in a diversity of cellular subtypes at multiple points in nervous system development, including in glia (29–33). Despite these gaps in knowledge, there has been no global cell type–specific analysis of transcriptional changes related to *FMR1* expansions in the human brain to date.

To understand the molecular and cellular perturbations associated with fragile X expansion in the human brain, we applied single-nuclei RNA-sequencing (snRNA-seq) to postmortem frontal cortex and cerebellar hemisphere of individuals with *FMR1* premutations and controls. We identified changes in *FMR1* expression, cellular proportion, global gene expression, and oligodendrocyte cortical development that challenge current assumptions about molecular mechanisms underlying FXTAS pathogenesis and specifically implicate glial dysregulation as critical in fragile X molecular neuropathology.

## Results

Prior to tissue processing, we reviewed available medical records to ensure that clinical and neuropathological data were consistent with genetic diagnoses (Table 1). We used tissue samples from primarily BA10 and lateral cerebellar hemisphere. All cases here have been previously presented in prior published work (34–36). Although we focus on the premutation, we also studied two cases of FXS, to assess whether well-known effects on *FMR1* expression were present in our dataset.

**Table 1.** Demographic information for postmortem samples used

ID	Sample^*^	Region	Age	Race	Sex	PMI	RIN	Notes	Prior validation
1793	CON	FC, CBL	11	Black	M	19			
5408	CON	FC	6	Black	M	16	7.6		
5497	CON	FC, CBL	68	White	M	13	8		
5541	CON	FC, CBL	84	White	M	12	8.3		
5657	CON	FC, CBL	82	White	M	22	8.4		
AN10723	CON	CBL	60		M	24			
4555	FXPM (67)	FC, CBL	80	White	M	12		Clinical diagnosis of Parkinson’s disease	CGG repeat-primed PCR (Esanov 2016)
4664	FXPM (100)	FC, CBL	71	White	M	3		Progressive neurological decline, history of hydrocephalus	^§^CGG repeat-primed PCR (D’Gama 2015, Esanov 2016)
4751	FXPM (88)	FC, CBL	21	White	M	5		Perinatal ischemic event, seizures	CGG repeat-primed PCR (Esanov 2016)
5006	FXPM (150)	FC, CBL	85	White	M	5	7.1	FXTAS neuropathology	^§^CGG repeat-primed PCR (D’Gama 2015, Esanov 2016)
5212	FXPM	CBL	80	White	M	12		FXTAS neuropathology, progressive neurological decline	CGG repeat-primed PCR (Esanov 2016), western blotting (Tran 2019)
5529	FXPM (58)	FC	89+^†^	White	M	16	7.1	FXTAS neuropathology	western blotting (Tran 2019)
5746^‡^	FXPM (116)	CBL, BA22	80	White	M	22		Progressive neurological decline, middle cerebellar peduncle sign	western blotting (Tran 2019)
4806	FXS	FC, CBL	9	White	M	22	4.3	FXS, gene deletion, no neuropathological findings	
5319	FXS (450+)	FC, CBL	71	White	M	17	6.6	FXS, full mutation, no neuropathological changes	CGG repeat-primed PCR (Esanov 2016)

^*^(approximate FMR1 repeat size if applicable)

^†^age deidentified

^‡^misclassified as FXS in Tran 2019 despite only partially reduced FMR1 protein; BA22 used only for *SI Appendix*, Fig. S8, sample not included in frontal cortex analysis

^§^in addition to premutation, mosaic full mutation was identified in small percent of cells, records consistent with premutation-associated pathology and clinical presentation

Repeat size if applicable ascertained from clinical records and prior published work. Validation summary describes published reports of cases in addition to clinical records. CON: nondisease control, FXPM: fragile X premutation, FXS: fragile X syndrome. FC: frontal cortex (BA10: Brodmann area 10; 4555 and 5529 listed as prefrontal cortex), CBL: lateral cerebellar hemisphere [(section 5); 1793, 5212, 5746, 4555 listed as cerebellum], BA22: Brodmann area 22 (posterior superior temporal cortex), RIN: RNA integrity number, PMI: postmortem interval.

**Fig. 1.**
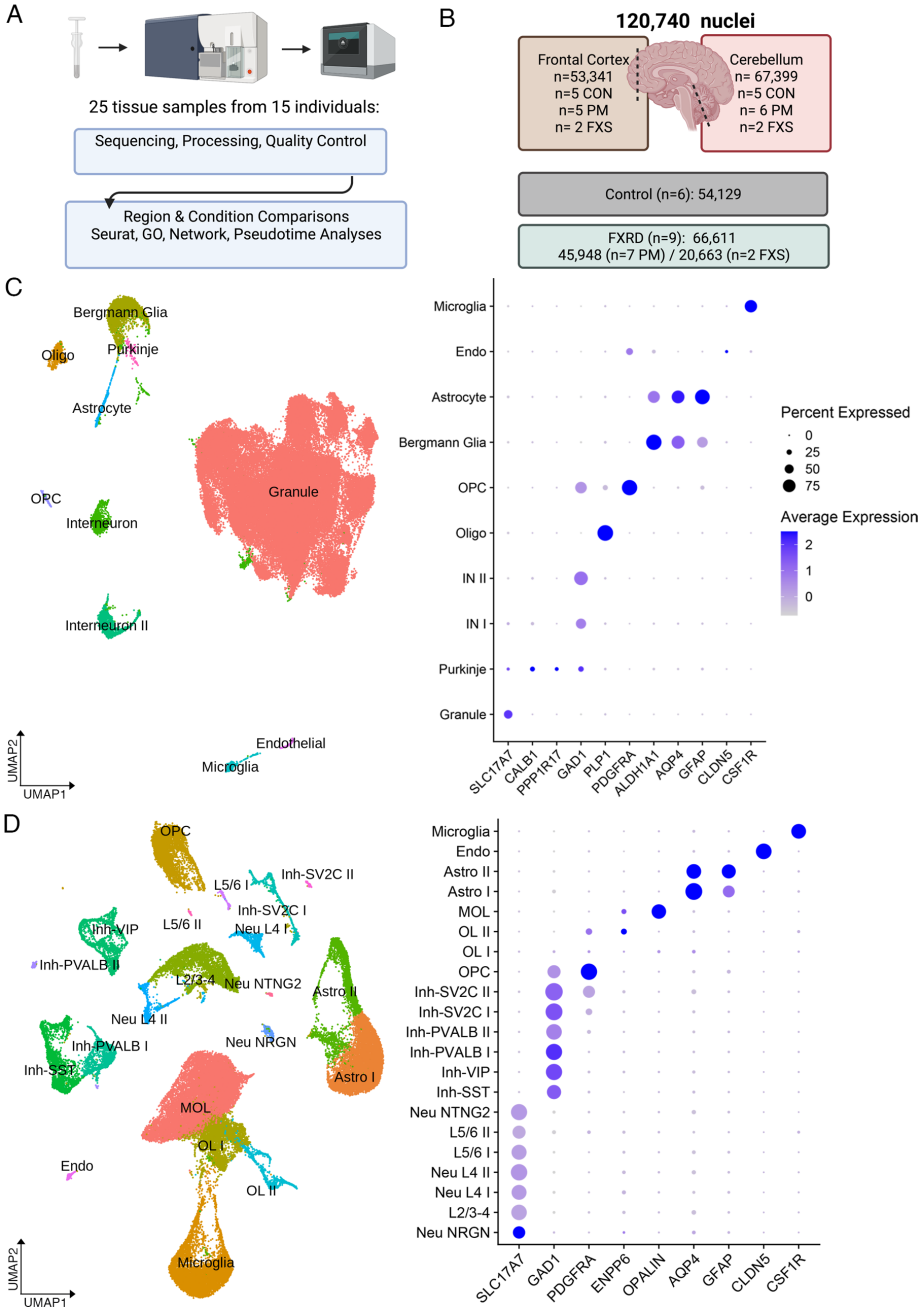
Cell type–specific analysis of frontal cortex and cerebellum. (*A*) Sample preparation included dounce homogenization, sucrose centrifugation, fluorescent nuclear sorting, and nuclear encapsulation. (*B*) Summary of sample size and final filtered nuclei number per condition and region. (*C*) Cerebellar UMAP plot and dot plot of cell type–specific markers. (*D*) Frontal cortex UMAP plot and dot plot of cell type–specific markers in the frontal cortex. Abbreviations- FXRD: Fragile X-related disorders, Endo: endothelial, Astro: astrocyte, MOL: mature cortical oligodendrocyte, Oligo: mature cerebellar oligodendrocyte, OPC: oligodendrocyte progenitor, IN: interneuron, OL: oligodendrocyte lineage, Neu: excitatory neuron, Inh: inhibitory neuron, L: layer-specific excitatory neuron clusters.

The majority of premutation cases had either clinical and/or neuropathological evidence of FXTAS (Table 1). We identified one case that in the past was mistakenly categorized as FXS, but whose clinical records and genetic testing revealed it to be a premutation (Table 1). One case of FXS due to a deletion of *FMR1* was included given the known shared molecular consequences of *FMR*1 deletion and trinucleotide expansion (37) and neither FXS case had neuropathological abnormalities noted. We used dounce homogenization followed by sucrose gradient centrifugation to isolate nuclei (Fig. 1*A*), fluorescent nuclear sorting, and bioinformatic processing to ensure only high-quality nuclei were evaluated, leaving ~120,000 nuclei for downstream analysis (Fig. 1*B* and Table 2 and *SI Appendix*, Fig. S1 *A*–*C*). There were no differences between premutation and control or FXS groups in age, postmortem interval (PMI), or RNA integrity number (RIN) (*SI Appendix*, Fig. S2*A*). There was a reduction in RIN in FXS compared to controls, which may be related to increased metabolic stress that has been reported in FXS (38). There was no association between RIN and PMI and age (*SI Appendix*, Fig. S2*B*). We additionally validated predicted functional changes in FMRP expression with western blotting of frontal cortex samples (*SI Appendix*, Fig. S2*C*).

**Table 2.** Filtered nuclei number

Cluster	CON	FXPM	FXS	Total
Cerebellum				
Granule	25,067	24,237	9,831	
Oligo	366	542	130	
Bergmann glia	643	1276	334	
Interneuron	497	848	155	
Interneuron II	395	930	97	
Microglia	219	268	85	
Astrocyte	263	276	104	
OPC	157	327	79	
Endothelial	87	63	20	
Purkinje	59	21	23	
Cerebellar Total	27,753	28,788	10,858	67,399
Cortex				
Inh Neu	3,916	2,049	1,081	
Exc Neu	2,776	2,689	868	
OPC	2,411	1,467	1,591	
OL I	3,173	1,058	522	
OL II	250	81	630	
MOL	4,381	6,890	1,767	
Astro I	4,264	914	1,577	
Astro II	1,719	468	614	
Microglia	3,348	1,518	1,092	
Endo	138	26	63	
Cortex total	26,376	17,160	9,805	53,341

Inhibitory neuron represents sum of all inhibitory neuron subclasses, and excitatory neuron represents sum of all excitatory neuron subclasses.

### Cell Type Annotation.

We applied known cell type–specific markers to assess the specificity and accuracy of unsupervised clustering (Fig. 1 *C* and *D*). For both prefrontal cortex and cerebellar hemisphere, we identified specific classification of cellular subtypes for both neurons and glia. Layer-specific excitatory neuron and inhibitory neuron subclusters in the frontal cortex were consistent with broader prior published annotations (*SI Appendix*, Fig. S3) (39–42).

There were distinctions between the cerebellum and cortex in overall cell composition. In the frontal cortex, we identified several distinct clusters appearing to reflect different states of oligodendrocyte development, including PDGFRA + oligodendrocyte progenitor cells (OPCs), two intermediate clusters (OLI-PTPRZ1+ and OLII-TFC7L2/ENPP6+), and a mature myelinating oligodendrocyte (MOL) cluster (*SI Appendix*, Figs. S3 and S4). We compared the transcriptional profile of OLI and OLII to oligodendrocyte lineage clusters identified in mouse (43) and found that OLI gene expression resembled mouse committed oligodendrocyte progenitors (COPs) and OLII resembled immature, newly formed, nonmyelinating oligodendrocytes. We also identified astrocyte groups similar to the protoplasmic astrocytes (astrocyte I) and fibrous astrocytes (astrocyte II) previously described in snRNA-seq studies of postmortem brain (39). On the contrary, in the cerebellum, although granule cells accounted for most nuclei captured, as expected, we also identified a cerebellar-specific Bergmann glia cluster, as well as interneuron and interneuron II that resembled molecular layer interneurons I and II, respectively, as described in ref. 44. OLI and OLII clusters were not identified in cerebellar samples. Given our sample size, we may be inadequately powered to detect less frequent cellular subtypes known to reside within these brain regions, including functionally heterogeneous oligodendrocyte subtypes.

### FMR1 Expression.

**Fig. 2.**
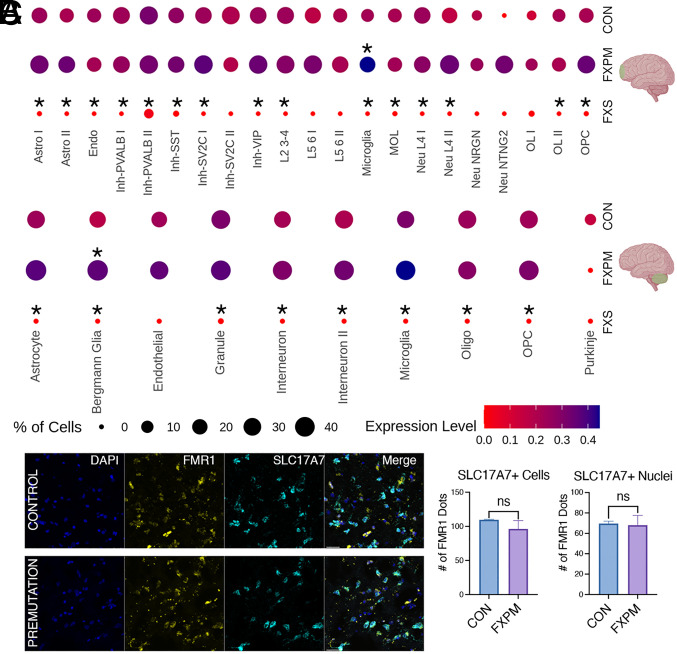
Modest FMR1 changes in premutation postmortem brain. (*A*) snRNA-seq of frontal cortex and cerebellar changes in FMR1 expression in premutation cases and FXS cases vs control. Cluster abbreviations as in Fig. 1. In premutation cases, only cerebellar Bergmann glia and cortical microglia demonstrated significant increases in FMR1 mRNA expression. There was widespread FMR1 reduction in FXS cases despite the smaller sample size. (Asterisk above the dot indicates padj < 0.05 for condition vs control comparison.) Expression level indicates average of scaled log-normalized expression. (*B*) Representative image from fluorescent in-situ hybridization demonstrating comparable FMR1 expression in excitatory neurons (SLC17A7 marker) in premutation vs control in the frontal cortex. (Scale bar, 30 μm). (*C*) No significant difference in FMR1 mRNA was seen in SLC17A7+ nuclei, or, using an extended boundary, SLC17A7+ cells (two-tailed *t* test, *P* = 0.91 (nuclei), *P* = 0.46 (cells)). n = 2 control, n = 3 premutation.

Analysis of individual cluster *FMR1* expression revealed cell type–specific effects of fragile X status on *FMR1* transcription in the premutation cases; specifically, modest but significant upregulation in only a few glial populations, as well as cell type–specific heterogeneity (Fig. 2). In fact, in premutation cases, the only clusters that demonstrated significantly increased FMR1 mRNA expression in either the frontal cortex or cerebellum were nonneuronal, including cerebellar Bergmann glia and cortical microglia (Fig. 2*A*). On the contrary, in fragile X syndrome, despite a smaller n, we identified total abrogation of *FMR1* expression, as expected, in both cases of the full mutation and gene deletion (Fig. 2*A*). This provides important proof of principle that expected transcriptional signatures are present within the snRNA-seq data. Indeed, despite the smaller sample size and nuclei number, reduced *FMR1* expression was robust among different clusters in FXS in both neuronal and glial subpopulations across the brain, consistent with the large effect size of this genetic driver.

To rule out inadequate power as a reason for lack of significant FMR1 regulation in cortical neuronal premutation populations, we also grouped inhibitory and excitatory neuronal subclusters and similarly identified no significant upregulation in this pseudo-bulk analysis (*SI Appendix*, Fig. S5). Thus, in general, the lack of significant upregulation of *FMR1* mRNA in most neuronal subclusters in the premutation cases is not due to a lack of power. Rather, it suggests that overall, the increase in FMR1 expression in the brain caused by the premutation is far more modest than the four to eightfold increase observed in blood, and shows a preferential impact on glia, in the regions assessed here. Given our sample size, we are underpowered to detect changes in very rare cell types, such as Purkinje cells, and cannot rule out significant changes in those clusters.

To validate these findings, we conducted fluorescent in-situ hybridization (RNAscope) on a small subset of premutation and control samples. Like the snRNA-seq findings, we found no evidence for significant upregulation of FMR1 in excitatory neurons. (Fig. 2 *B* and *C*). In RNAscope, we have the advantage of defining a larger border around the nuclei to incorporate an estimation of cellular expression outside of just the nucleus defined by DAPI expression. Using either the nuclear or extended “cellular” border, we found the same result (Fig. 2*C*). We also found no evidence for increases in FMR1 expression overall in all cortical nuclei/cells, and there were no major outliers in the distribution of FMR1 expression within samples that drove these findings. (*SI Appendix*, Fig. S6 *A* and *B*).

Individual donors demonstrated heterogeneity with respect to FMR1 expression changes (*SI Appendix*, Fig. S7A-C). Premutation repeat size has been found to be a critical factor on the molecular and clinical level in FXTAS, with a significant correlation between repeat size and age of symptom onset as well as reduced FMRP and increased FMR1 transcription (17, 45). We interestingly found a significant positive correlation with cortical microglia FMR1 expression and repeat size (*SI Appendix*, Fig. S7*B*), in agreement with past work and suggesting that the modest cell type–specific increases in FMR1 expression observed may have clinical relevance. However, it was not generally a specific donor that drove FMR1 changes. Thus, the findings of changes in FMR1 expression are robust across samples.

### Changes in Cellular Abundance in FXTAS.

**Fig. 3.**
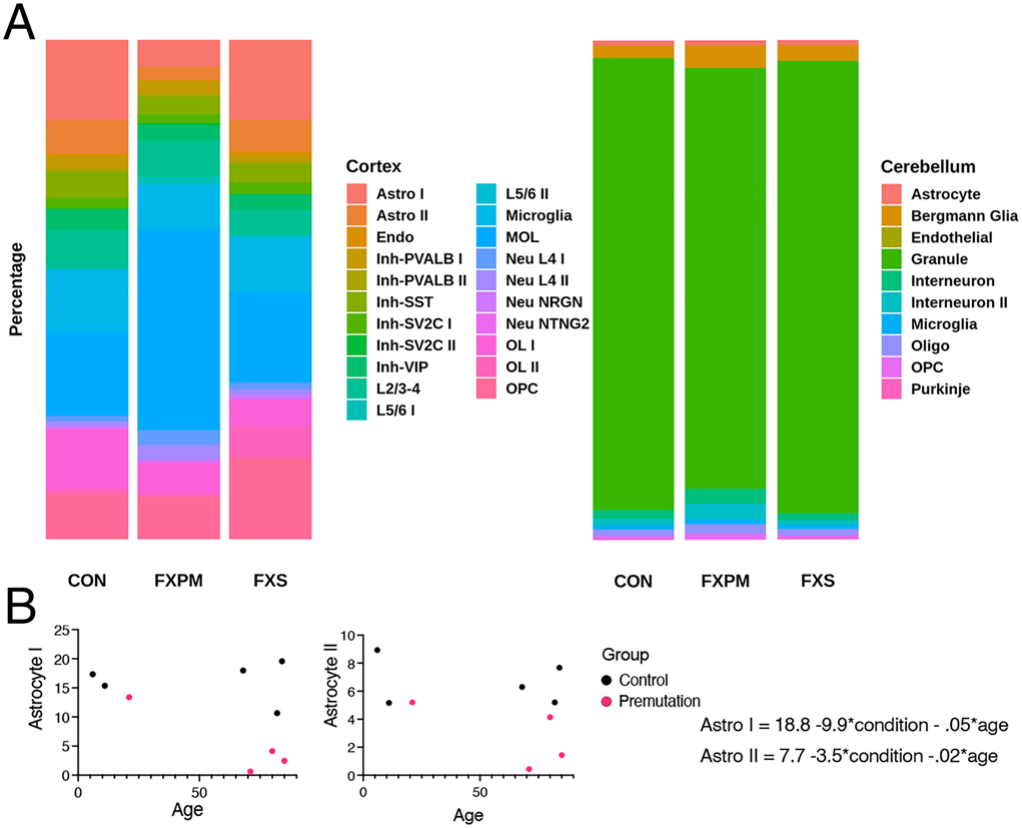
Change in cellular abundance in postmortem brain. (*A*) Average percentage of nuclear composition in the frontal cortex and cerebellum. (*B*) For premutation and control samples, linear regression analysis was conducted to determine the effect of condition and age on cellular abundance, using the equation cluster % = β_0_ + β_1_*condition + β_2_*age. There was a significant effect of premutation condition on both cortical astrocyte groups (β_1_ *P* < 0.05), but not age (β_2_ *P* > 0.05). 89+ aged individual is omitted from graph but was included in regression analysis.

Given that we used an unbiased nuclear collection, we can use nuclei number as a proxy for cellular composition (Fig. 3). We identified changes in relative cell types in the frontal cortex (BA10) in association with the premutation that were unexpected. Premutation cases demonstrated fewer-than-expected astrocytes, observations not due to the effect of age, and not observed in the cerebellum (Fig. 3 *A* and *B*, Supplemental Information File 1). To determine whether this was specific to BA10, we assessed BA22 in one premutation sample and identified astrocyte levels to be similar to control, suggesting that these findings may reflect a subcortical specific finding (*SI Appendix*, Fig. S8). We were surprised to see no significant changes in neuronal proportions. We wondered whether age-associated changes in neuronal composition might mask subtle effects of the premutation on neuronal clusters, given that age-associated changes in inhibitory density have also been observed (46–48). In this case, we identified a significant age-related decline in inhibitory neuron/total neuron composition in the frontal cortex as previously reported (49), although there was no detectable effect of fragile X mutation status on this decline (*SI Appendix*, Fig. S9 and *Supplemental Information File 1*). Note, although the changes in inhibitory neuron density have been previously reported with respect to age, our experimental design cannot distinguish between aging and maturational changes during development. We also identified age-related changes in cortical OPCs and microglia, but there was no impact of FMR1 status in those cluster proportions. Thus, we identified alterations in astrocyte number specific to the frontal cortex in premutation cases.

In the cerebellum, changes in abundance in premutation cases also recapitulated past neuropathological studies, specifically previous work demonstrating cerebellar Purkinje cell loss and Bergmann cell gliosis in individuals with the premutation (50) (Fig. 3*A*). Consistent with this, we identified a trend toward relatively fewer Purkinje cell nuclei, and greater Bergmann glial cell nuclei, in the cerebellum of premutation carriers versus controls (*SI Appendix*, Supplemental Information File 1), suggesting that loss of Purkinje cells may contribute to FXTAS signs and symptoms.

### Differential Gene Expression & Gene Ontology.

We next interrogated global patterns of differentially expressed genes (DEG) (Datasets S1–S6 and *SI Appendix*, Tables S1–S7) in each cluster subtype between conditions. We observed that many neuronal clusters in FXS demonstrated a marked tendency toward upregulation of gene expression, reflecting a derepressed state, an effect not observed to the same extent in neuronal premutation comparisons. Additionally, premutation vs FXS comparison DEG lists were larger than premutation or FXS vs control comparisons, suggestive of divergent gene expression regulation between these two closely related conditions.

Although there were no significant differences in age or PMI between control and premutation groups, these variables are important to consider given the wide age range we include and developmentally dependent clinical phenotypes. To explore the effects of age and PMI, we used PCA to determine whether these variables impacted gene expression variability and reran differential expression analysis in MAST (*SI Appendix*, Fig. S10) including these variables independently. Most comparisons demonstrated high overlap in DEG significant findings, and changes in FMR1 expression were equivalent (*SI Appendix*, Fig. S10 and Datasets S7–S18). It was only for primarily FXS comparisons, in which inclusion of age rarely altered the DEG list substantially. This suggests that our main findings of FMR1 changes in expression, and global DEG in premutation cases, are not due to confounding effects of these variables.

We conducted gene ontology (GO) analysis to identify perturbed biological processes. In premutation cases relative to controls, we identified evidence of disrupted neuroregulatory roles of glia, in both cortex and cerebellum (*SI Appendix*, *Supplemental Information Files 2 and 3*). For example, in multiple glial clusters, biological process terms including synaptic functioning, axon guidance, and neurotransmitters were enriched. On the contrary, classical glial terms were not ubiquitously found in different condition comparisons. For example, myelination terms were uniquely enriched in the OLI population in premutation vs control comparisons, but in the OPC population, in FXS vs control comparisons. There was also evidence of neuronal dysfunction: neurons in premutation cases demonstrated evidence of altered neuronal function and structure, such as synaptic signaling and neuronal arborization.

Other notable processes that were revealed through GO analysis included widespread enrichment of protein folding and prion disease terms, as well as signaling cascades implicated in *FMR1* pathophysiology [including Wnt signaling, phosphoinositide 3 kinase (PI3K) signaling, and MAPK signaling (33, 51, 52)] in both neurons and glia in both premutation and FXS comparisons in multiple populations (*SI Appendix*, *Supplemental Information Files 1 and 2*). Interestingly, however, in any individual cell type, GO terms in FXS vs CON and PM vs CON comparisons were distinct, demonstrating the unique biological changes occurring in these conditions.

The observed increase in FMR1 expression in premutation cases that has been observed in the past has been ascribed to increased transcription as opposed to changes in mRNA stability (53). Our findings here of isolated increases of FMR1 in glial subtypes suggest that this may reflect cell type–specific transcriptional mechanisms. To assess this further, we inspected the DEG lists from the cell clusters that demonstrated significant FMR1 upregulation in cortical microglia and cerebellar Bergmann glia and used the gene lists to identify core transcriptional regulators (*SI Appendix*, Tables S8 and S9). We identified transcriptional regulators, including IRF1 and STAT2, that have not been previously implicated in FXTAS pathogenesis, that warrant future study, particularly given some reports of immune-mediated disorders preceding FXTAS symptoms in individuals with the premutation (54).

### FMRP Network Dysregulation.

**Fig. 4.**
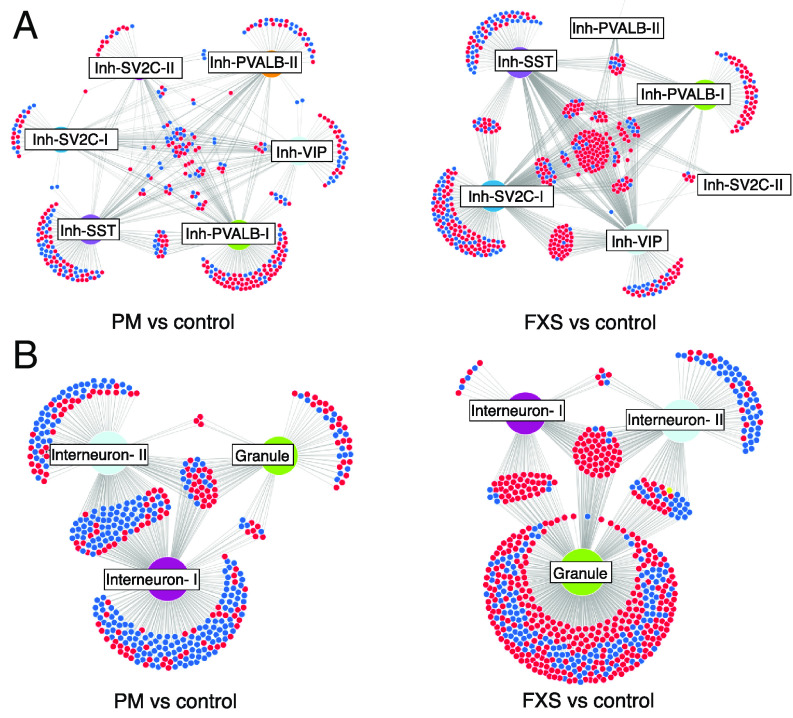
Network analysis of neuronal FMRP target dysregulation. Frontal cortex inhibitory neurons (*A*) demonstrate a hub of common derepressed FMRP target genes in FXS (*Right*), which is not observed in premutation cases (*Left*). (*B*) Cerebellar neurons in FXS also demonstrate a shared derepressed hub, with an opposite pattern in premutation cases. Different cell types demonstrate disproportionate effects of FMRP dysregulation depending on mutation status. Cluster abbreviations as in Fig. 1. Red, upregulation; blue, downregulation; yellow, opposite regulation in different cell types.

We wondered whether the patterns observed in DEG lists were reflective of altered FMRP network functioning or more downstream, nonspecific effects. We noticed that known FMRP targets were present in both neuronal and glial differentially expressed gene lists in both FXS and premutation cases, so we next used network analysis and visualization to understand changes in FMRP network function. To do this, we generated a list of FMRP targets previously functionally validated in human cells and used DiVenn to visualize cell type– and network-specific patterns of regulation of these target genes among significantly differentially expressed gene lists (Dataset S19) (35, 55–57). We identified both expected and unexpected network perturbations. For example, cortical inhibitory neurons in FXS demonstrated a hub of shared, derepressed FMRP target genes, consistent with loss of FMRP’s role as a transcriptional repressor (Fig. 4*A*). This network convergence was absent in inhibitory cortical neurons in premutation cases, but present in cerebellar neurons in FXS (Fig. 4*B*), demonstrating that there are distinct effects on FMRP target dysregulation in FXS and FXTAS. Importantly, it suggests that despite variable FMRP loss in premutation cases, neuronal transcriptional derepression is not the main biological consequence, consistent with global DEG analysis. Intriguingly, in cerebellar premutation neurons, frequent downregulation of FMRP targets was observed. Additionally, there was differential impact on different cell types. For example, in FXS cases, granule cells demonstrated a disproportionate burden of the FMRP dysregulation not seen in premutation cases (Fig. 4*B*).

**Fig. 5.**
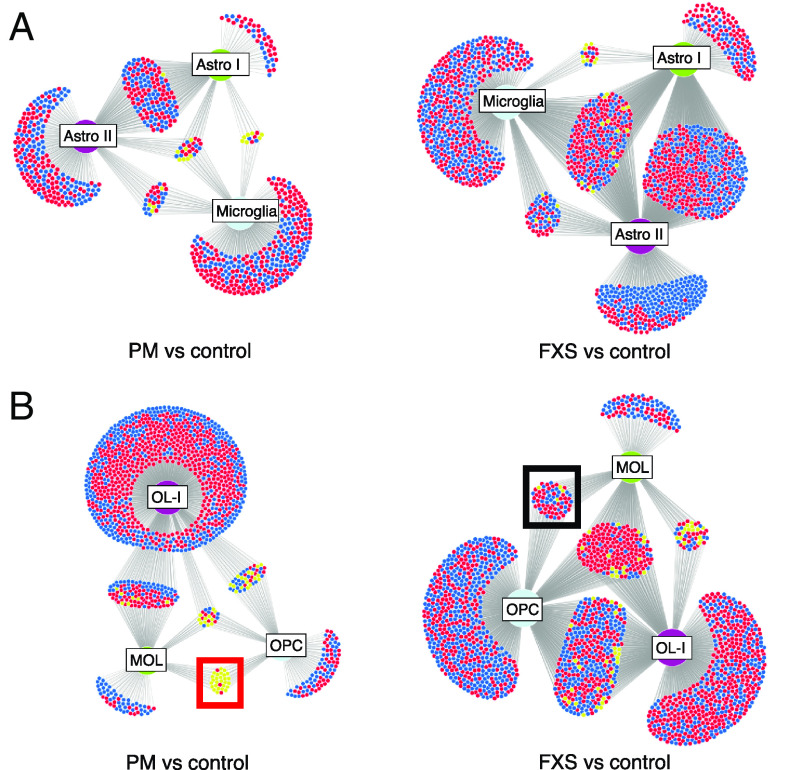
Network analysis of cortical glial FMRP target dysregulation. (*A*) Frontal cortex astrocytes and microglia demonstrate cell type–specific effects of FMRP network dysregulation in premutation cases (*Left*) and FXS (*Right*) (*B*) Oligodendrocyte lineage in premutation cases demonstrate uniquely divergent regulation of FMRP targets between OPCs and MOLs (red box); this pattern is not seen in FXS (black box). Cluster abbreviations as in Fig. 1. Red, upregulation; blue, downregulation; yellow, opposite regulation in different cell types.

Examination of FMRP target dysregulation in glia in premutation cases also revealed an intriguing example of network discordance (Fig. 5 *A* and *B*). Unlike the majority of FMRP targets that were dysregulated in multiple cell types in the same direction, in premutation cases, in OPCs and MOLs, there was notable opposite regulation of the same *FMRP* target genes. (Fig. 5*B*). This was not observed to the same extent in FXS and highlights a disease-specific “switch” in gene expression regulation in two closely related oligodendrocyte cell clusters. Additionally, in several glial subtypes in FXS, such as astrocyte II, there was a preponderance of unique FMRP targets which demonstrated downregulation (Fig. 5*A*). This is contrary to the known role of FMRP as a repressor and potentially highlights alternative cell type contexts of FMRP in vivo, such as in mRNA stabilization (58).

### Pseudotime Analysis Implicates Abnormal Oligodendrocyte Development in FXTAS.

**Fig. 6.**
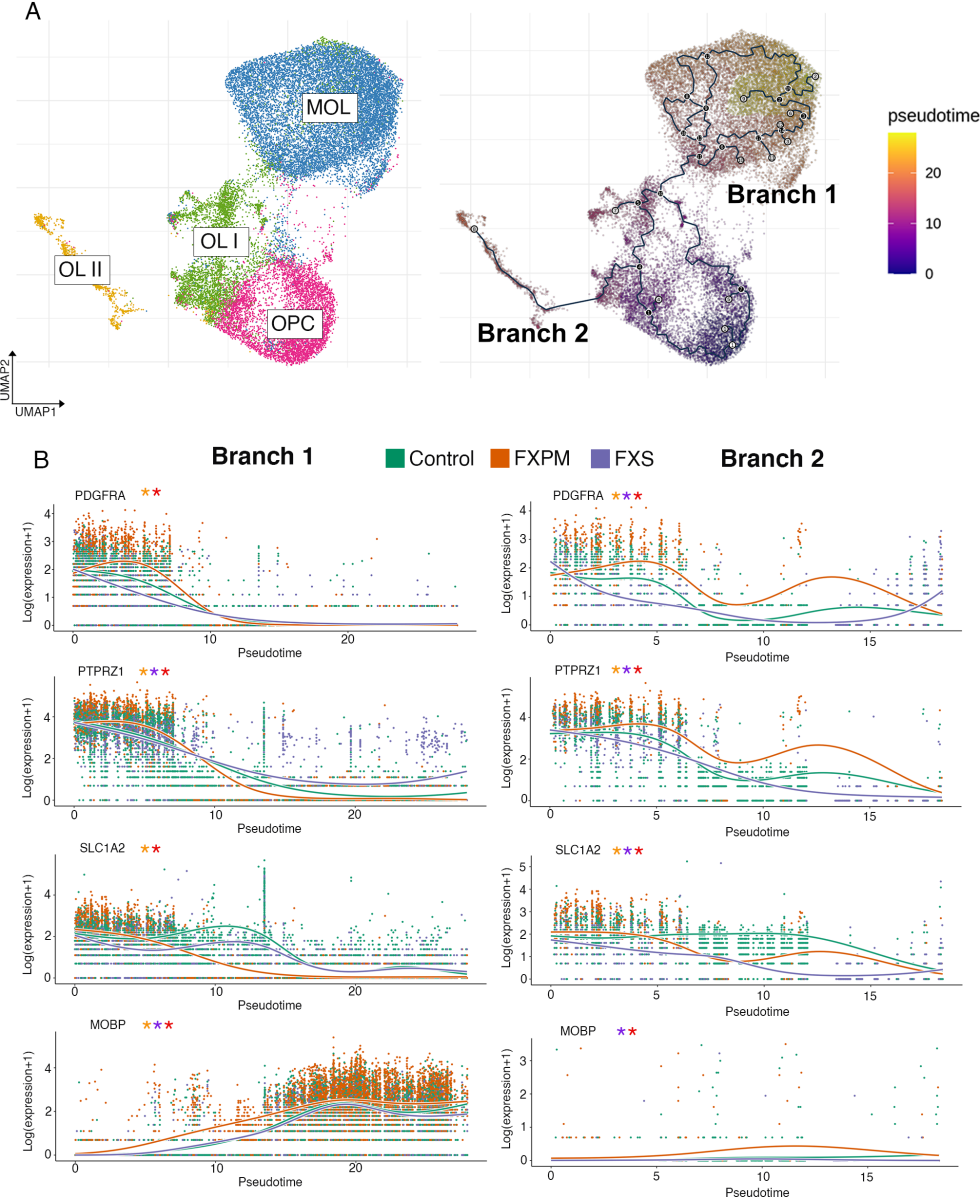
Pseudotime analysis of cortical oligodendrocytes. (*A*) Reclustering of major oligodendrocyte clusters identifies two major trajectory branches identified in the frontal cortex. (A) Expression of oligodendrocyte markers track in both trajectories as expected, with significant differences between conditions across pseudotime. Orange asterisk: significant difference between premutation and control; purple: significant difference between FXS and control; red: significant difference between premutation and FXS, see *SI Appendix*, Methods for details on the Wald test.

Given the observed changes in oligodendrocyte FMRP network function in premutation cases, and because FMRP is known to be critical in oligodendrocyte development (29, 30, 32, 59), we hypothesized that oligodendrocyte development would be uniquely perturbed in premutation cases. To assess this, we conducted a pseudotime trajectory analysis of oligodendrocyte clusters in the frontal cortex. Following reclustering with Monocle3 (Fig. 6*A* and *SI Appendix*, Fig. S11 *A*–*B*), we identified 2 distinct trajectories, one that went from OPC →OLI→MOL (branch 1), and another that went from OPC →OLI → OLII (branch 2). As expected, immature and mature markers tracked as expected with pseudotime (*SI Appendix*, Fig. S11*C*); however, there were marked differences across pseudotime in glial developmental markers (Fig. 6*B*), suggesting that *FMR1* disruption impacts key processes in oligodendrocyte development in a temporally dependent manner. Indeed, the distribution of cell types along pseudotime trajectories revealed noticeable shifts in the distribution density in premutation cases (*SI Appendix*, Fig. S11*D*).

**Fig. 7.**
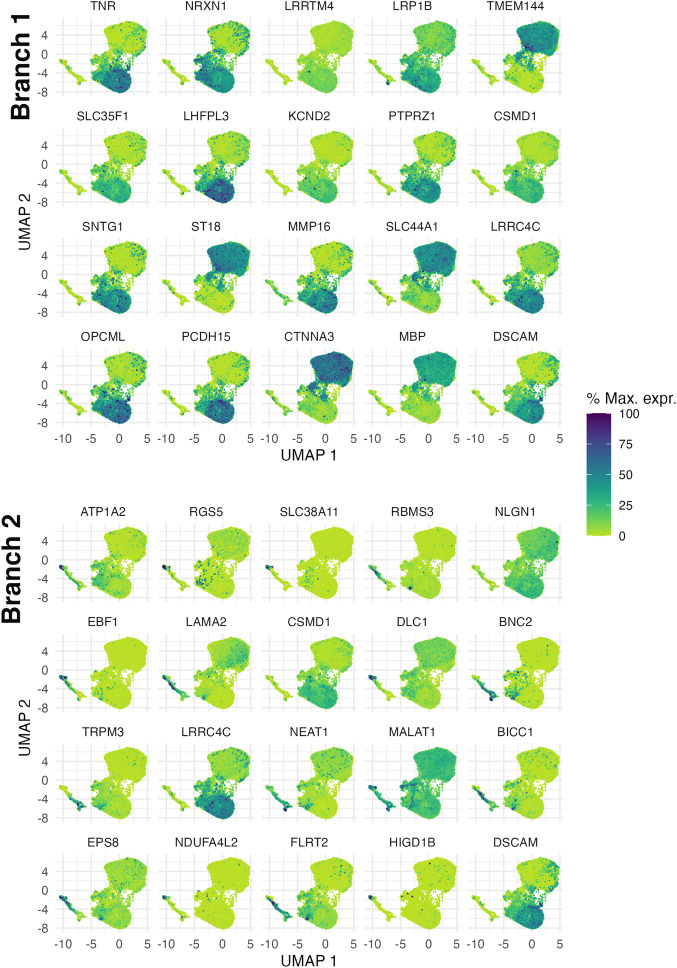
Spatial autocorrelation analysis of differentially expressed genes along pseudotime in the frontal cortex. Top 20 genes that change with pseudotime in branch 1 (*Top*) and branch 2 (*Bottom*). Genes were selected by q value and Moran’s I statistic.

**Fig. 8.**
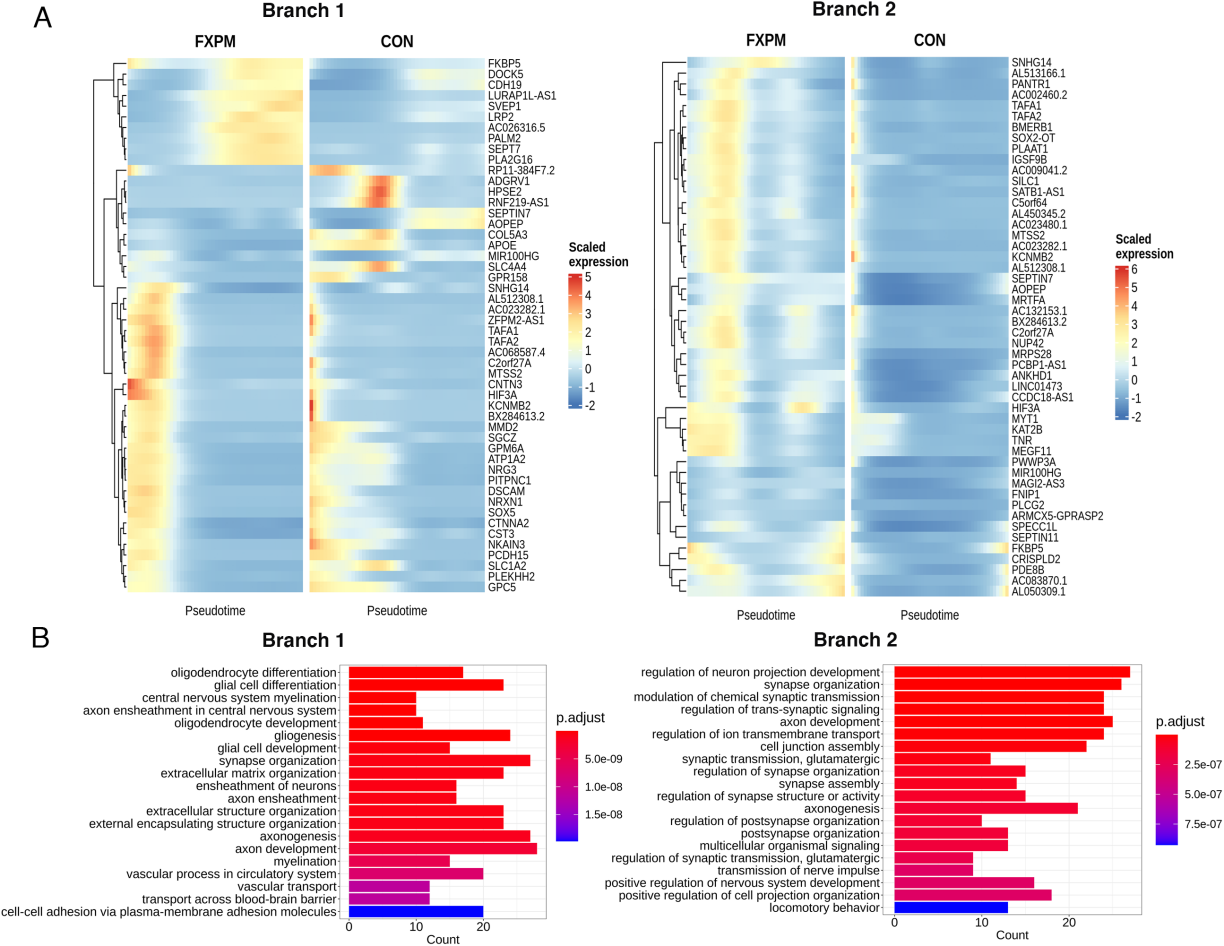
Differentially expressed genes along oligodendrocyte pseudotime in the frontal cortex. (*A*) Heatmap of top 50 genes with strongest difference in expression (pair-wise comparison, Wald statistic) in branch 1 (*Left*) and branch 2 (*Right*) between premutation cases and controls. (*B*) Top 20 gene ontology biological processes enriched in branch 1 (*Left*) and branch 2 (*Right*) in the premutation vs control comparison

We conducted differential expression analysis both along the pseudotime trajectory and between conditions (*SI Appendix*, Table S7 and Dataset S20). Using Moran’s I test to assess spatial autocorrelation of gene expression along the two trajectories, we identified several genes (*NRXN1*, *CSMD1,* for example) that have been implicated in cognitive function and aging, but not to our knowledge specifically through oligodendrocyte function (60–66) (Fig. 7). Additionally, assessment of top differentially expressed genes between conditions in pseudotime revealed an intriguing phenomenon in frontal cortex premutation cases: Genes that were expressed very early in narrow pseudotime windows in control cases were shifted later and with widened expression windows in premutation cases in both trajectories. (Fig. 8*A*) This was not the case in cortical FXS vs control comparisons (*SI Appendix*, Fig. S12 *A* and *B*), or cerebellar oligodendrocyte pseudotime analysis of premutation vs control comparisons (*SI Appendix*, Fig. S12 *C* and *D*), which suggests unique perturbation of early oligodendrocyte development in the frontal cortex, in premutation cases. GO analysis of biological process dysregulation identified from these significantly differentially expressed genes between different conditions in pseudotime revealed a similar pattern in the top 20 most significant biological processes––prominent inclusion of multiple terms implicating neuroregulatory roles of glia (Fig. 8*B*). Additionally, in branch 1, myelination terms were uniquely identified in premutation comparisons (Fig. 8*B*).

## Discussion

We present a cell type–specific analysis of gene expression of fragile X-related disorders in the human brain. We identified changes in FMR1 mRNA expression and cell type–specific gene expression that sheds light on molecular perturbations associated with *FMR1* and specifically highlights an important role for glial molecular dysregulation in premutation pathology.

### Modest Glial FMR1 Upregulation in Premutation Cases.

It has been shown that the FXTAS CGG repeat expansion leads to increased FMR1 expression and mRNA-rich intranuclear inclusions that impact RNA-binding protein function, supportive of an RNA gain-of-function hypothesis (9, 19, 67, 68); however, our data suggest that FMR1 mRNA expression in premutation cases, at least in the brain regions analyzed here, is more modestly affected than has been observed in peripheral blood cells and furthermore that it preferentially affects glial cells more than neurons. Given the robust elimination of FMR1 mRNA expression observed here in association with FXS, regardless of the genetic driver (trinucleotide expansion vs gene deletion), nuclei cluster, or brain region, we are confident in the validity of our approach. In the premutation cases, we identified modest upregulation of *FMR1* expression, limited to some glial subclusters in both cerebellum and cortex demonstrating significant increases. Rather than extreme neurotoxic increases in FMR1 mRNA, our findings suggest a modest, ~1.3 fold, increase in FMR1 transcript levels, paralleling past studies of brain homogenate (9, 26). Findings of FMR1 upregulation in cortical microglia are intriguing given past reports of altered microglial activation in FXTAS human cases (31). It opens up the possibility that these prior reported changes may at least be in part directly driven by the effects of the premutation within microglia, as opposed to solely an inflammatory response to the microenvironment.

Although we cannot rule out that neural cells expressing toxic levels of FMR1 transcript are selectively vulnerable and preferentially lost with time, our cellular proportion analysis (see below) does not support this interpretation, as one would expect remaining cells in populations that are disproportionately lost to have relatively higher increases in *FMR1* mRNA. Finally, changes in FMR1 mRNA expression were comparable between clusters known to be vulnerable to premutation-associated intranuclear inclusions (neurons, astrocytes) and those known to be spared (oligodendrocytes), arguing against inclusion presence/nuclear measurement as being a confounding factor in FMR1 mRNA measurement. Our in-situ analysis using an extended cellular border also argues against nuclear measurement as being the reason for our findings for modest FMR1 changes in premutation cases. Finally, past work suggests that the FMR1 canonical poly(A) site is used comparably in normal and premutation cases (69), suggesting that alternative 3′ variants that might be underrepresented in our sequencing approach are an unlikely source for the differences observed in premutation cases here. These findings challenge the RNA toxicity hypothesis that dominates the literature, at least at the levels extrapolated from studies of peripheral blood. Given the ongoing controversy around the relative importance of FMR1 mRNA toxicity and other hypothesized mechanisms in in vitro and animal models (27, 70–72), our work is particularly relevant.

### Alterations in Cellular Abundance Implicate Cortical Glia.

Changes in cell proportions in premutation cases in both cerebellum and cortex implicate glial dysregulation. In premutation cases, we identified a proportional decrease in cortical astrocytes, findings not explained by age. The relationship of basal FMR1 mRNA expression, change in FMR1 expression, and cellular proportion was not straightforward, arguing against a simplistic relationship between cellular proportion and FMR1 toxicity. For example, glial cells in the frontal cortex that demonstrated modest differential expression of FMR1 also demonstrated the most marked changes in cellular proportion. This may be related to earlier developmental time points that are impacted, cellular extrinsic effects on survival and proliferation, or both. Regardless, given the findings of global brain atrophy and the reported decline in executive functioning reported in FXTAS (8, 73–75), these changes in cellular proportion warrant further exploration of the role of glia in FXTAS-associated cognitive symptoms and in other cortical areas.

### Alterations in Known and Unique Cellular Functions Revealed with Differential Gene Expression.

We identified global transcriptional alterations associated with premutation status that support the now well-established principle that glia play central roles in neurodevelopment and disease (76–79). For example, OPCs are known to form synaptic-like structures and respond to neuronal activity (80) and glia more generally are critical in neuronal development, axonal integrity, and behavior (81–83). Differential gene regulation in premutation glia frequently identified perturbations in the maintenance of synaptic structure and function and altered neurotransmission in multiple glial lineage clusters. Indeed, white matter abnormalities in fragile X-related disorders more broadly may reflect subtle disruption of glial regulatory roles in neuronal homeostasis. Determination of whether these glial abnormalities contribute causally to clinical symptomatology or represent a secondary response to neuronal dysfunction will require further work in human model systems. Classical glial function was also uniquely disrupted in premutation cases. For example, in premutation cases, we identified upregulation and enrichment of myelination terms in the intermediate committed oligodendrocyte progenitor OLI cluster, a finding also identified in pseudotime analysis of branch 1, the branch that uniquely contains the OLI–MOL transition (78, 84).

GO analysis also revealed widespread enrichment of terms implicated in protein processing and prion disease. Interestingly, it has recently been shown that the polyglycine region of FMRpolyG (the toxic protein generated from repeat-associated non-AUG translation of *FMR1*, a posited contributor to neurotoxicity in FXTAS) has a prion-like domain with low complexity similar to the so-called prion proteins in yeast, and that it may propagate cell to cell in a prion-like manner (70). Although none of the cases showed the rapid progression or typical clinical features of actual human prion protein (PrP)-related prion disease, such as Creutzfeldt–Jakob disease, our enrichment analysis is consistent with protein processing dysregulation being broadly relevant to *FMR1* dysregulation in both premutation and FXS cases. For example, a heterogeneous increase in de novo protein synthesis in FXS cases is well described and is posited to contribute to challenges in drug development (23, 85). Other processes previously reported to be involved in *FMR1* pathophysiology also appeared in GO lists, including Wnt, MAPK, and PI3K signaling. However, these signaling pathways did not appear ubiquitously, and our unbiased approach may thus inform future research on unexpected critical cellular targets in which these pathways may play outsized roles, such as cerebellar Bergmann glia. Given that cell type–specific transcriptional regulatory mechanisms may contribute to differences in FMR1 regulation, we also identified transcription factor motifs that may provide critical information for future mechanistic work into the molecular steps that lead from the premutation expansion to changes in FMR1 mRNA.

### Unexpected Glial FMRP Network Functioning in Premutation Cases.

FMRP reduction may contribute to clinical symptoms in premutation carriers in a developmentally distinct manner compared to FXS. We observed marked patterns of FMRP network dysregulation in both FXS and in cases with premutations, but these patterns were quite distinctive. For example, neurons, but not glia, demonstrated evidence of coherent FMRP network derepression in FXS. Furthermore, this phenomenon was notably absent in premutation cases, with certain networks demonstrating increased repression or incoherent regulation between cell types of a similar lineage. This analysis suggests that although FMRP loss may be present in both FXS and FXTAS, FXTAS does not represent simply a “milder” hit on FMRP network dysregulation. Rather, there are cell type–specific, region-specific, and developmental-specific factors which fundamentally alter FMRP network functioning.

### Specific Perturbations in Cortical Oligodendrocyte Development in Premutation Cases.

Finally, our pseudotime analysis identified specific alterations in oligodendrocyte developmental trajectories in premutation cases in the frontal cortex and identified potential targets and pathways that may mediate these effects. For example, neurexin/neuroligin signaling has been implicated in neuronal signaling-dependent glioma growth (86). Premutation status specifically perturbed a narrow window of very early gene expression in oligodendrocyte pseudotime; this opens up intriguing lines of inquiry as to how this developmental hit may relate to a primarily degenerative phenotype. It also warrants follow-up given the plethora of developmental phenotypes observed in individuals with the premutation (87). Additionally, although myelination-related genes are known FMRP targets (23, 32, 59), these were not ubiquitously impacted in the oligodendrocyte lineage in different conditions or pseudotime (Fig. 8*B*), revealing the importance of cellular and developmental context. Unexpectedly, we did not identify a branch including both intermediate oligodendrocyte stages and MOLs. It is possible, given our small sample size, that additional rare oligodendrocyte states were missed, given the high functional heterogeneity of the oligodendrocyte lineage within the brain (43, 80).

Given the small sample size of FXS cases, we use it primarily as a comparison for premutation biology and proof of principle that expected changes in biology are observed. However, replication of past findings including absent FMR1 expression (5, 88) and evidence of metabolic stress (89, 90) corroborate known molecular neuropathology of the disorder (38). Thus, our findings on FXS, representing over 20,000 nuclei, serve as proof of principle that expected changes in *FMR1* biology are present in these nuclear transcriptome datasets. Our work demonstrates the need for more comprehensive study of fragile X in human tissue directly in a variety of different cell types, particularly given that our sample size is inadequate to identify meaningful changes in rare but potentially relevant cell types.

In conclusion, we provide compelling evidence from the human brain regarding cell type–specific molecular neuropathology that helps contextualize the clinical heterogeneity associated with genetic variation at the *FMR1* locus in neurodevelopment and neurodegeneration and specifically implicates glial dysregulation in premutation pathology. Our findings in premutation postmortem brain, in light of known neuropathological and imaging abnormalities, support the interpretation of FXTAS as a disorder defined by glial dysfunction and warrant consideration of FXTAS as a “gliodegenerative” disorder (79, 91).

## Materials and Methods

### Samples.

Postmortem human tissues were obtained from the NIH NeuroBioBank, University of Maryland Brain and Tissue Bank, and the Autism BrainNet according to their institutional review board approvals and following written informed consent. Initial dissection of tissue for brain bank specimens was done under standardized procedures using sequential sectioning. Research on these deidentified specimens and data was performed at Boston Children’s Hospital with approval from the Committee on Clinical Investigation. Fragile X mutation status/repeat size was verified through direct review of deidentified clinical records and crossreferenced with prior published validation of the same cases (Table 1). Most of the premutation cases had clinical symptomatology or neuropathological evidence of FXTAS (Table 1). Samples were group matched for age and sex, but no cutoffs were utilized to exclude any cases for PMI and RIN (*SI Appendix*, Fig. S2).

### Western Blotting.

Approximately 25 to 50 mg frozen frontal cortex was homogenized in RIPA buffer + protease inhibitors and centrifuged, and total protein content was then quantified. Laemmli sample buffer was added to the protein supernatant and boiled for 5 min. Equal amounts of protein (10 μg) were loaded onto precast SDS-Page gels with molecular weight ladders. Samples were transferred to membranes, blocked with Licor block (Lincoln, NE), cut along molecular weight markers, and incubated in primary antibody overnight diluted in block at four degrees. Following four washes in tris-buffered saline + tween (TBS + T), blots were incubated with LI-COR secondary fluorescent antibodies in the dark at room temperature for 1 h. After further washing including a final wash of TBS, the blots were scanned on a LI-COR Odyssey imager. The following primary antibodies and dilutions were used: GAPDH (Cell Signaling # 2118S) 1:15,000; FMRP (Cell Signaling 4317S) 1:1,000.

### RNAscope Multiplex Fluorescent V2 Assay.

Frozen tissue blocks were mounted in prechilled Optimal Cutting Temperature compound media, and then placed on dry ice and stored at −80 °C. Ten micrometer sections were cut on a Leica CM1520 cryostat and mounted on charged slides. Fluorescent RNAscope was conducted as per Advanced Cell Diagnostics’ (ACD) instructions, including 30 min postfixation in chilled 10% NBF. A mixture of probes used (diluted 50:1 C1:C2 per ACD’s protocol) included Hs-FMR1-C1 (Cat #590731) and Hs-SLC17A7-C2 (Cat# 415611-C2). Fluorophores used were Akoya Biosciences Opal 520 (Cat #FP1487001KT, 1:1,000, FMR1) and Opal 570 (Cat #FP1488001KT, 1:1,000 to 1,500, SLC17A7). Four z-stacks were taken with identical settings from each slide on a Zeiss LSM 780 confocal. A maximum projection was generated in Fiji and then run through a semi-automated pipeline in Cell Profiler to count nuclei (defined by DAPI), cells (set boundary beyond DAPI), and mRNA puncta within nuclei and cells and for colocalization. The average of four stacks was taken for each sample/slide.

### Isolation of Postmortem Nuclei.

Frozen tissue (~25 mg) from either frontal cortex or cerebellar hemisphere (see table of demographics) was dissected at −20 and subjected to dounce homogenization followed by sucrose gradient centrifugation as previously described. Tissue was primarily BA10 or lateral cerebellar hemisphere although some samples were named only as frontal cortex or cerebellum. Nuclei were filtered and incubated for 5 min in 1:1,000 Hoechst (Invitrogen H3569, Waltham MA). A total of 10,000 Hoechst + nuclei from the suspension were then sorted directly into 10× Genomics RT buffer (Pleasanton, CA) on a chilled plate holder to remove doublets, debris, and dying nuclei on a FACS Aria (BD Biosciences, Franklin Lakes, NJ) with a low-pressure nozzle (*SI Appendix*, Fig. S2). Following sorting, reverse transcriptase enzyme was added on ice, and nuclei were immediately processed for encapsulation in the 10× Chromium controller. cDNA and libraries were prepared according to the 10× documentation protocol for 3′ gene expression v3 chemistry.

### Sequencing and Quality Control.

Samples were prepared and sequenced in matched groups to avoid confounding batch effects. The samples were sequenced on a NovaSeq 6000 (Illumina, San Diego, CA) to obtain high coverage and saturation and demultiplexed with bcl2fastq. CellRanger Count was utilized to generate count matrices with introns included, given intronic information is known to be informative for nuclear preparations. Sequencing metrics for each sample, in the output from CellRanger, are provided in *SI Appendix*, Table S10. To obtain a final high-quality nuclei set, filtering metrics were applied to nuclei in Seurat including: # UMIs> 500, # Genes > 250, log10GenesPerUMI (complexity measure) >0.8, and mitoRatio <0.1. Datasets were processed with SCTransform and integrated, and potential sources of variation were assessed with principal component analysis, with mitochondrial gene expression regressed out. SCTransform conducts normalization, variance stabilization, and regression of unwanted variation; it removes variation due to cellular sequencing depth. Unsupervised clustering was performed with different resolutions followed by application of known cell type markers. For cerebellar Purkinje and endothelial cells, which represented a very small percentage of the total nuclei sample, cell type markers (CALB1; CLDN5) were used to manually select cell clusters using the SelectCells feature in Seurat.

### Analysis.

Computationally intensive work was conducted on the Harvard Computing Cluster, O2, and Boston Children’s Hospital’s High-Performance Computing Cluster, E2. For detailed analysis methods, please see *SI Appendix* Methods.

## Supplementary Material

Appendix 01 (PDF)

Dataset S01 (XLSX)

Dataset S02 (XLSX)

Dataset S03 (XLSX)

Dataset S04 (XLSX)

Dataset S05 (XLSX)

Dataset S06 (XLSX)

Dataset S07 (XLSX)

Dataset S08 (XLSX)

Dataset S09 (XLSX)

Dataset S10 (XLSX)

Dataset S11 (XLSX)

Dataset S12 (XLSX)

Dataset S13 (XLSX)

Dataset S14 (XLSX)

Dataset S15 (XLSX)

Dataset S16 (XLSX)

Dataset S17 (XLSX)

Dataset S18 (XLSX)

Dataset S19 (XLSX)

Dataset S20 (XLSX)

## Data Availability

Data have been deposited in the controlled access section of DbGaP consistent with informed consent of tissue donors and can be accessed through the direct link https://www.ncbi.nlm.nih.gov/projects/gap/cgi-bin/study.cgi?study_id=phs000639.v2.p1 (92).
