# Supplementary material for: Glial dysregulation in the human brain in fragile X-associated tremor/ataxia syndrome
Source: Proc Natl Acad Sci U S A. 2023 May 30;120(23):e2300052120. doi: 10.1073/pnas.2300052120 (PMC10265985; doi:10.1073/pnas.2300052120)
Supplement: Supplementary file 1 — Appendix 01 (PDF) [file pnas.2300052120.sapp.pdf]

PNAS

Supporting Information for

Glial dysregulation in human brain in Fragile X-associated tremor/ataxia syndrome

Caroline M. Dias, Biju Issac, Liang Sun, Abigail Lukowicz, Maya Talukdar, Shyam K. Akula, Michael B. Miller, Katherine Walsh, Shira Rockowitz, Christopher A. Walsh

This PDF file includes:

Figures S1 to S12
Tables S1 to S10
Supplemental Information Files 1-3
Supplemental Methods
Supplemental References

Other supporting materials for this manuscript include the following excel files:

Datasets S1-S20

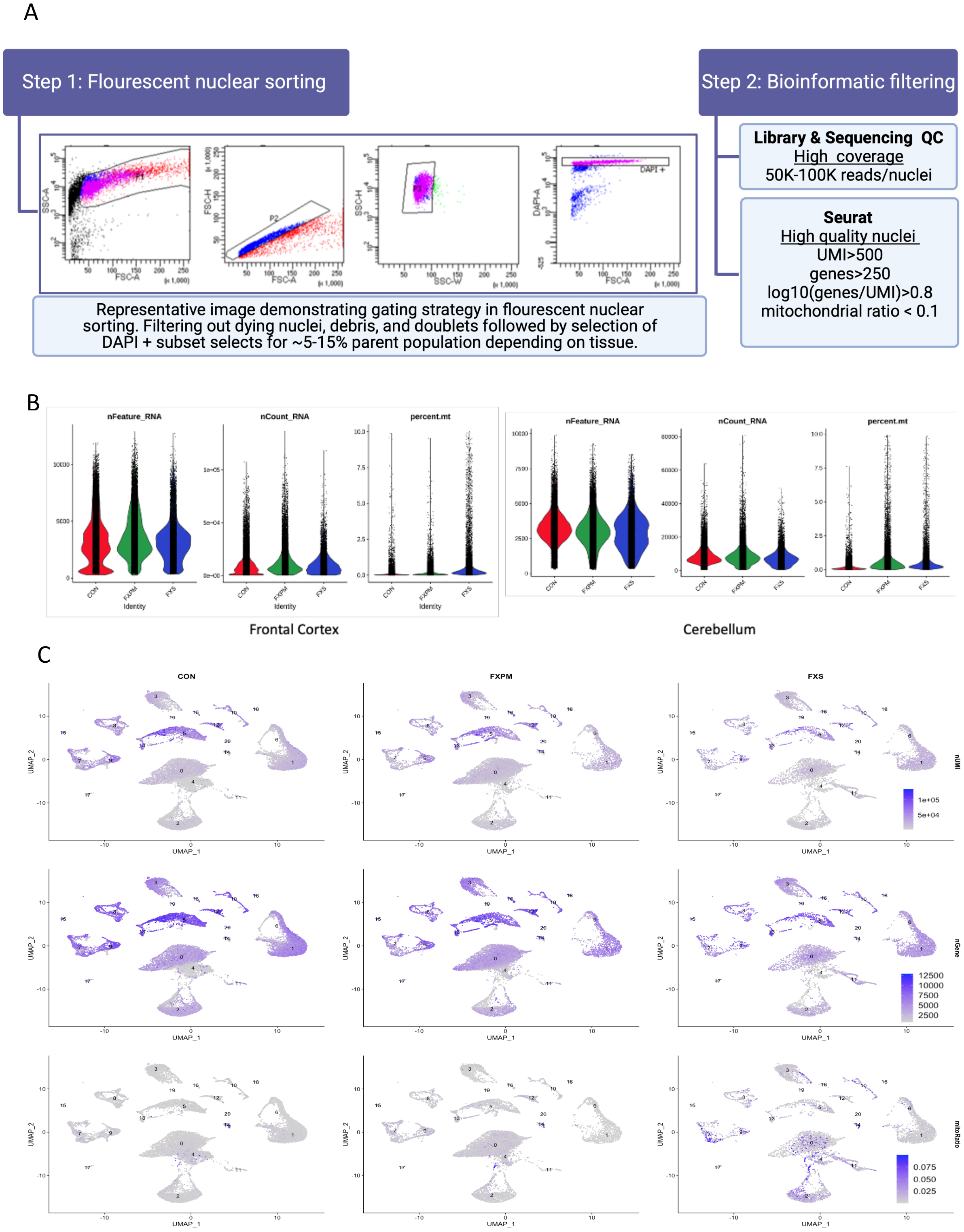

Figure S1: **Experimental and bioinformatic filtering pipeline and quality control metrics. A.** Representative gating strategy to obtain initial nuclei for encapsulation and subsequent bioinformatic processing. **B.** Overall group comparisons of number of genes/nuclei (features), counts/nuclei (UMI) and % mitochondrial gene expression in CON, FXPM, and FXS cases from left to right by region. **C.** UMAP depicting cell-type specific and condition specific differences in metrics in frontal cortex. Top row is number UMI, middle is number of genes, and bottom is mitochondrial gene percentage. (CON, FXPM, FXS from left to right). Clusters are described in Figure 1D.

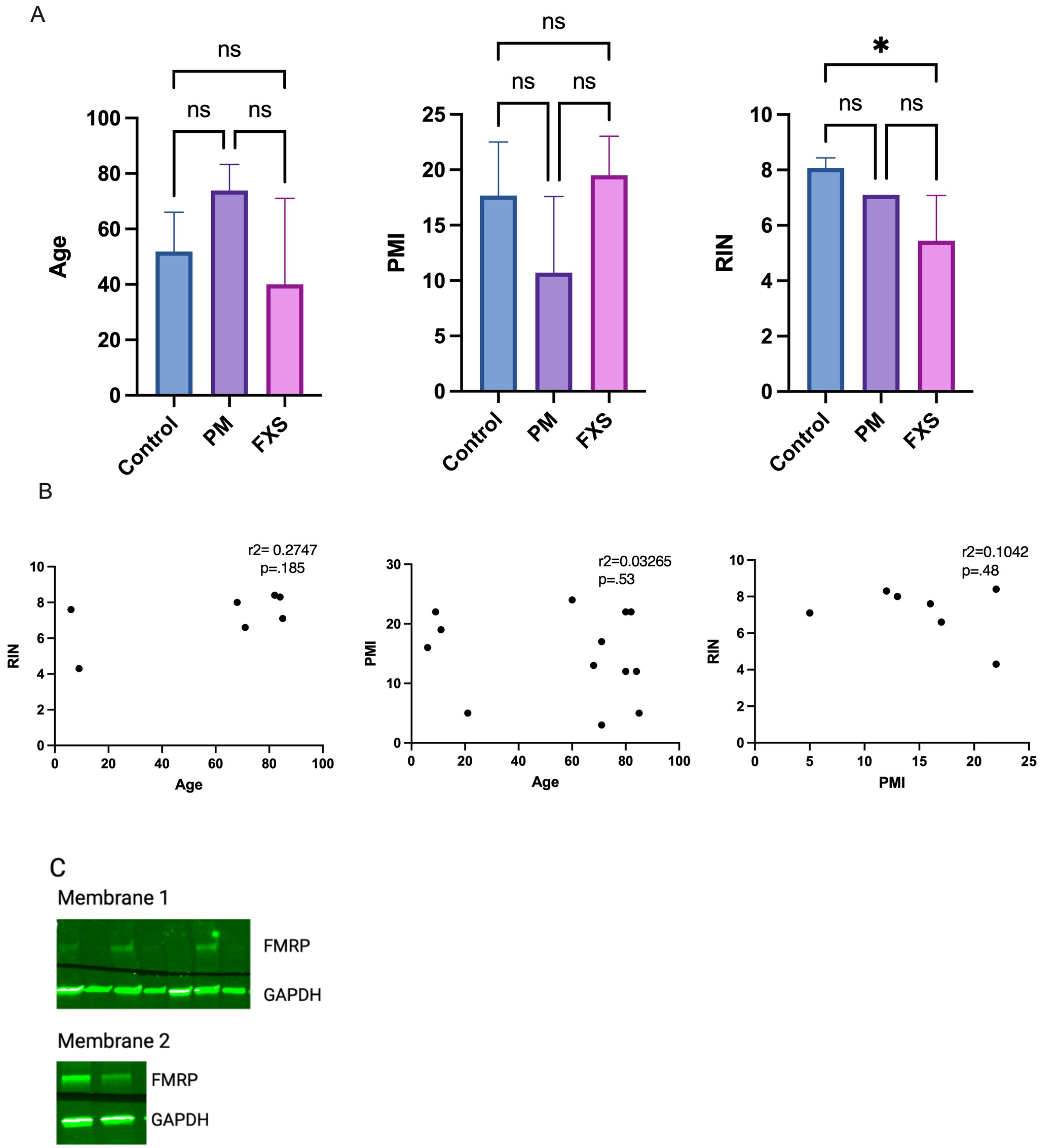

Figure S2: **Demographic and sample information. A.** No significant differences between groups with respect to age and PMI. FXS samples had lower RIN than control but not premutation cases ($p<.05$, one-way ANOVA, post-hoc Tukey's test). **B.** No significant correlation between RIN & Age, PMI & Age, or RIN & PMI among samples. **C**. Western blotting of frontal cortex premutation and FXS samples demonstrating variably reduced FMRP in premutation cases and absent FMRP in FXS cases. From left to right: 4664 (PM), 4806 (FXS), 5408 (CON), 5006 (PM), 5319 (FXS), 5657 (CON), 4555 (PM), 5497 (CON), 1793 (CON). Blots cropped for clarity.

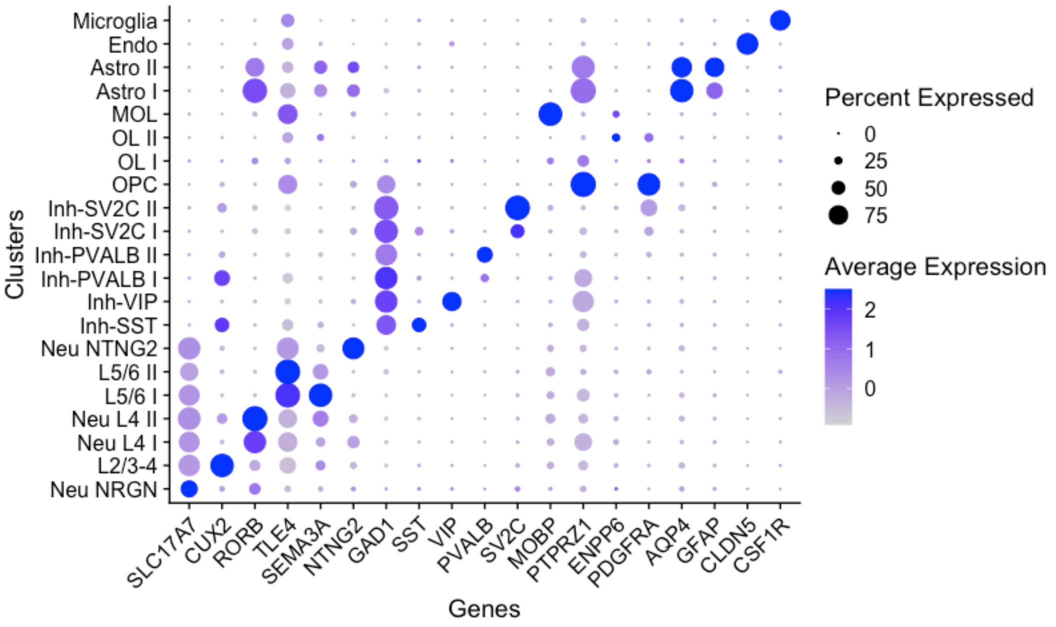

Figure S3: **High resolution frontal cortex cell-type specific markers are concordant with broader classification.** Note layer specific (CUX2, RORB, TLE4, SEMA3A, NTNG2) and inhibitory neuron subcluster specific (SST, VIP, PVALB, SV2C) expression.

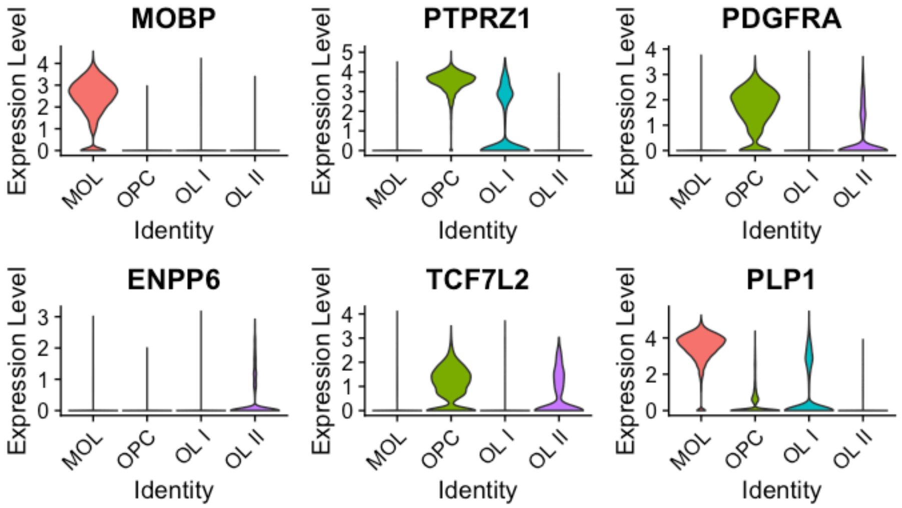

Figure S4: **Frontal cortex oligodendrocyte lineage markers.** Violin plots demonstrating distinct gene expression patterns in oligodendrocyte lineage clusters revealing a spectrum of developmental states.

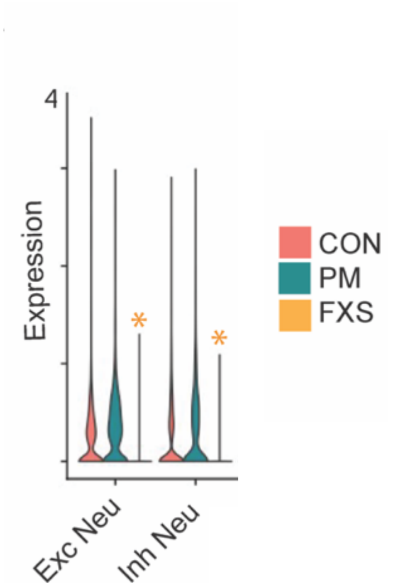

Figure S5: **Pseudobulk analysis of frontal cortex neuronal subpopulations.** This analysis reveals no significant changes in FMR1 mRNA in neurons in PM cases**.** Note significant downregulation in FXS cases despite the smaller n. Orange *: reduced FMR1 in FXS vs CON padj< .05.

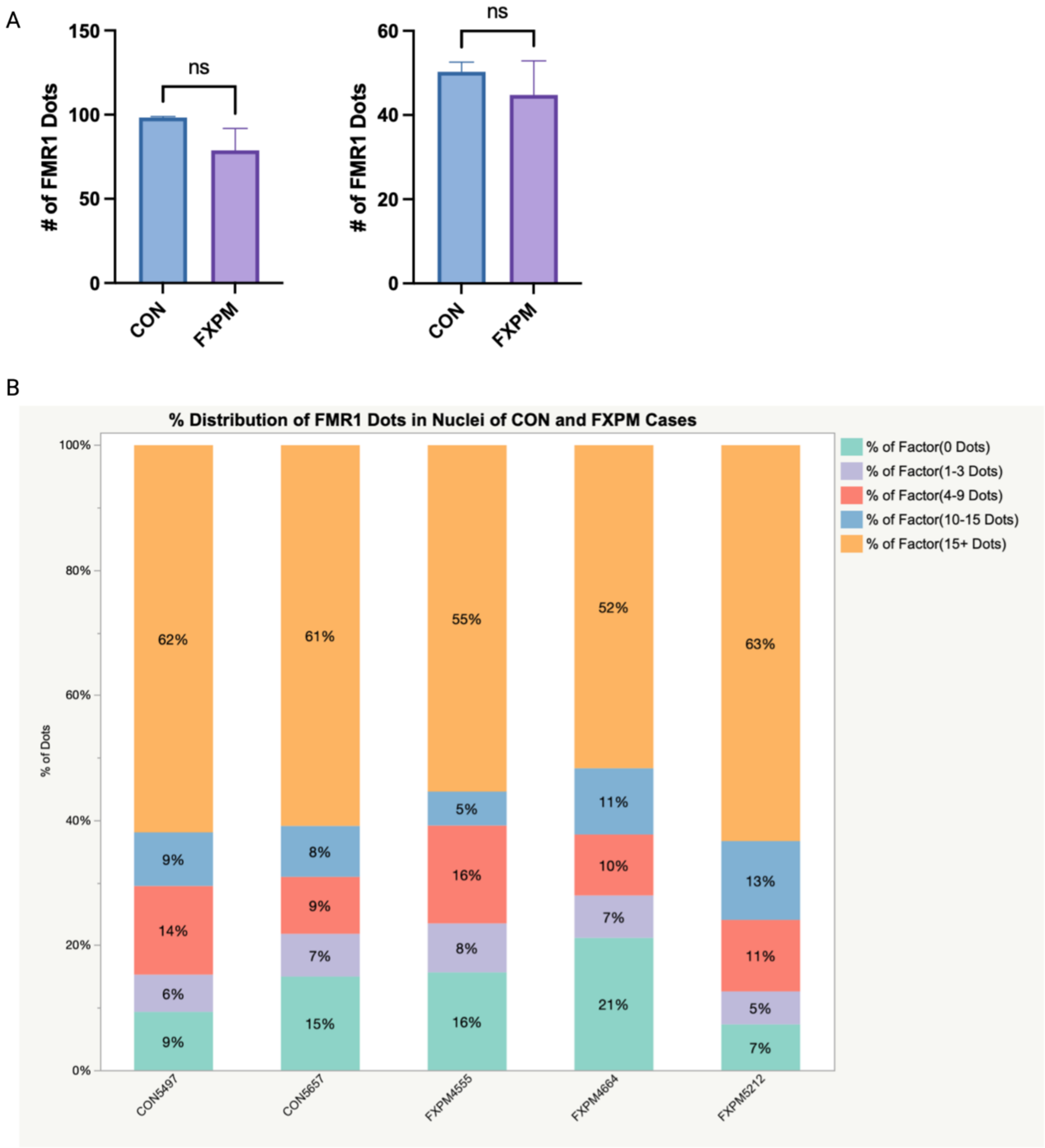

Figure S6: **FMR1 mRNA expression. A.** RNAscope demonstrates no change in FMR1 expression in all cells (left) or nuclei (right). (two-tailed t-test, p>.05) **B.** Distribution of FMR1 expression in all nuclei, binned by # of dots/nuclei, and separated by sample.

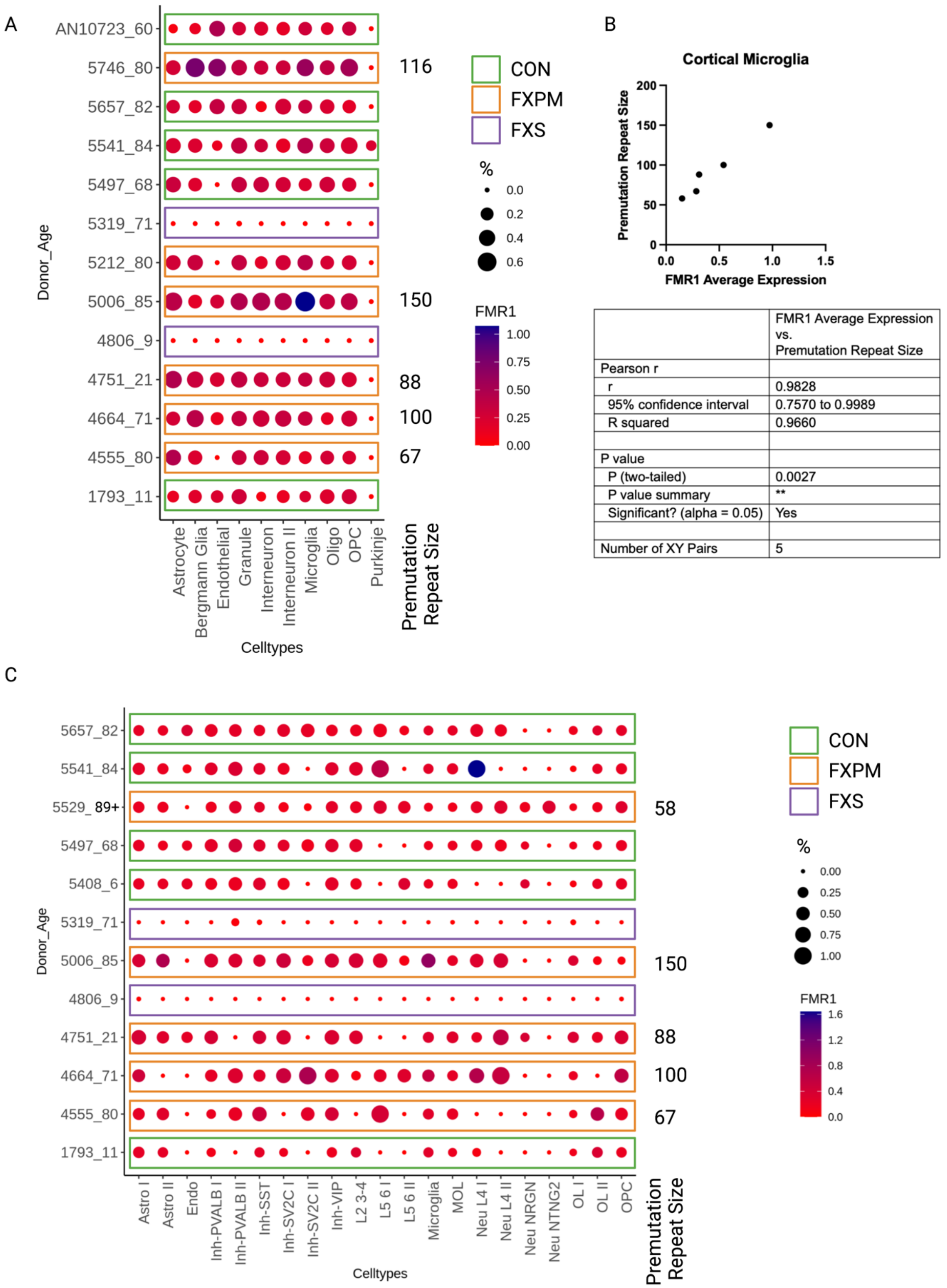

Figure S7: **Heterogeneity in FMR1 expression by donor. A.** snRNA-seq FMR1 expression in cerebellum separated by individual. **B.** There is a significant association between FMR1 expression in cortical microglia and premutation repeat size. **C.** Frontal cortex snRNA-seq FMR1 expression separated by individual. FMR1 heatmap shows average expression.

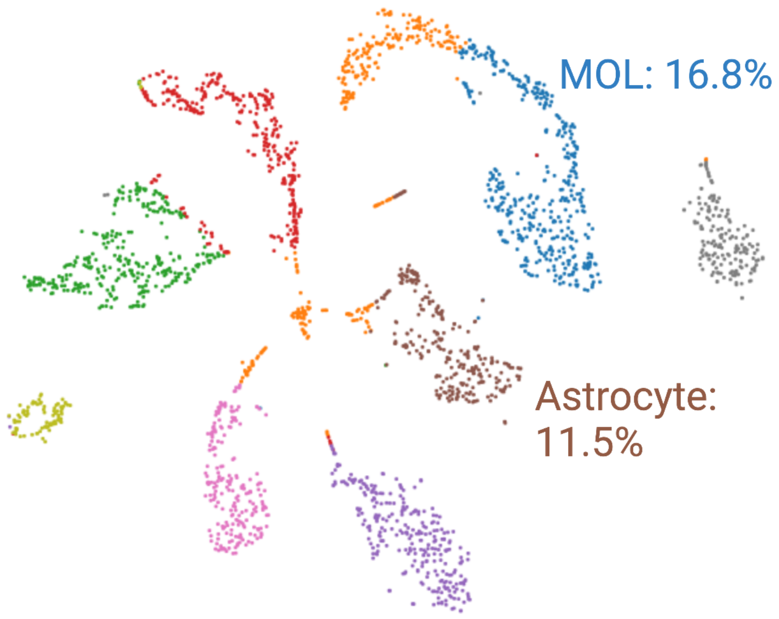

Figure S8: Lack of glial cluster alterations in premutation BA22. BA22 of 5746 demonstrates glial percentage comparable to control BA10, rather than premutation BA10 (see Table 2). Default clustering and tSNE plot output from Cell Ranger displayed, cell clusters for mature oligodendrocyte and astrocytes identified with PLP1 and AQP4 markers.

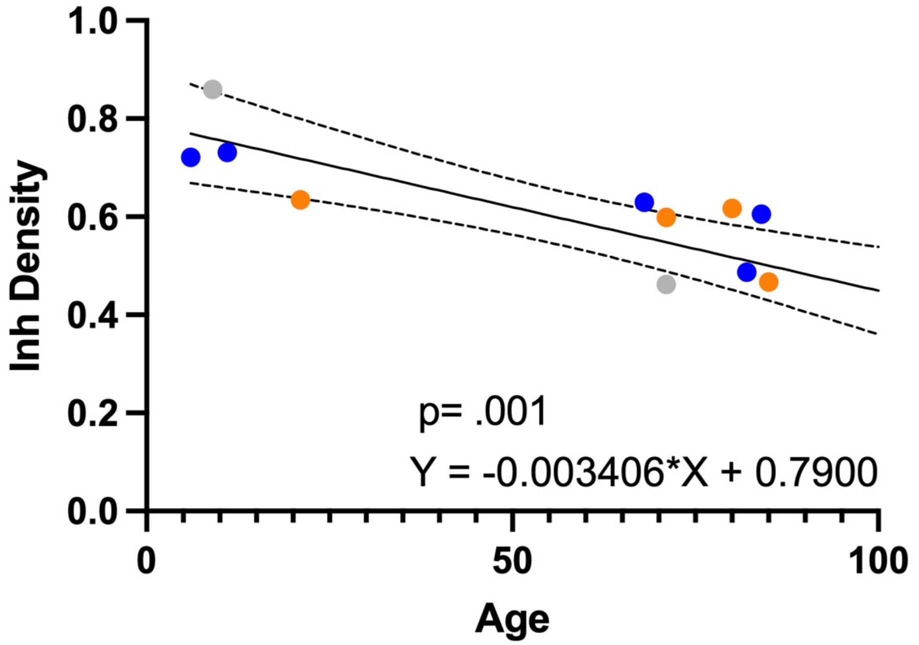

Figure S9: Association between cortical inhibitory neuronal density (inhibitory/total neuron percentage) and age regardless of Fragile X status. Blue: control, grey: FXS, orange: FXPM.

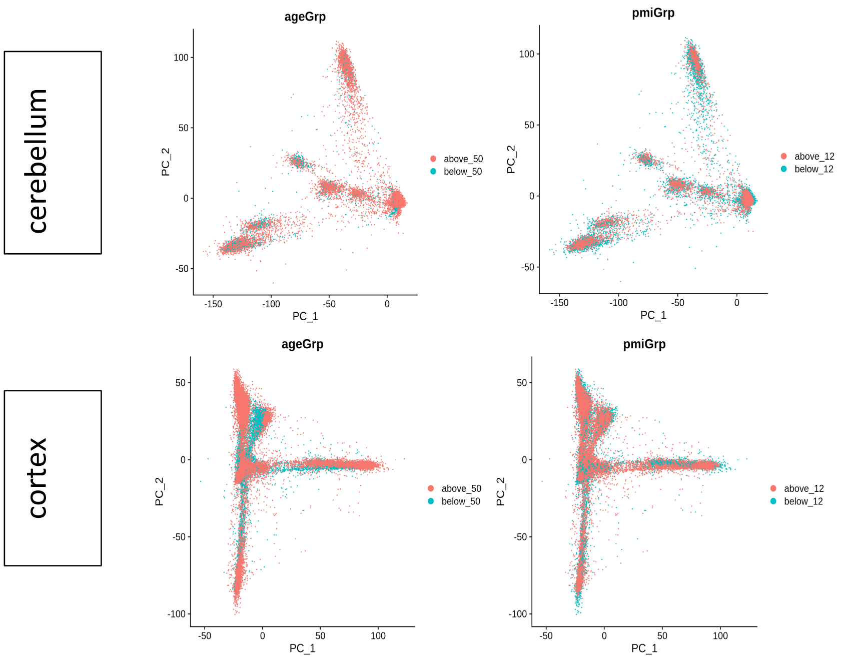

% Unique DEG in Age	PM vs CON	FXS vs CON	PM vs FXS
Granule	0	0	0
Bergmann Glia	0	0.2%	0.1%
Interneuron II	0	4.3%	4.1%

% Unique DEG in PMI	PM vs CON	FXS vs CON	PM vs FXS
Granule	0	0	0
Bergmann Glia	0.4%	0.8%	0.3%
Interneuron II	0	1.7%	5.9%

% Overlap in No Variable and Age Lists	PM vs CON	FXS vs CON	PM vs FXS
Granule	100%	100%	98.9%
Bergmann Glia	98.7%	61.9%	90.2%
Interneuron II	99.7	32.1	81.1%

% Overlap in No Variable and PMI Lists	PM vs CON	FXS vs CON	PM vs FXS
Granule	96.9%	91.4%	98.9%
Bergmann Glia	62.1%	93.1%	63.5%
Interneuron II	80.1	72.1	55%

Figure S10: Analysis of effects of age and PMI on gene expression. PCA plots demonstrating variability grouped by age (above and below 50 years old) and PMI (above and below 12 hours post-mortem). MAST was used to generate cell-type-specific differential expression lists with age and PMI independently and compared to gene lists without these variables. Inclusion of these variables did not add significant additional information (i.e. new unique DEG). The overlap of the no variable analysis and including age analysis, and no variable and PMI analysis, were largely concordant, except for some FXS comparisons. There was no change in significance of FMR1 (or lack thereof) within any of these gene lists although the precise padj varied.

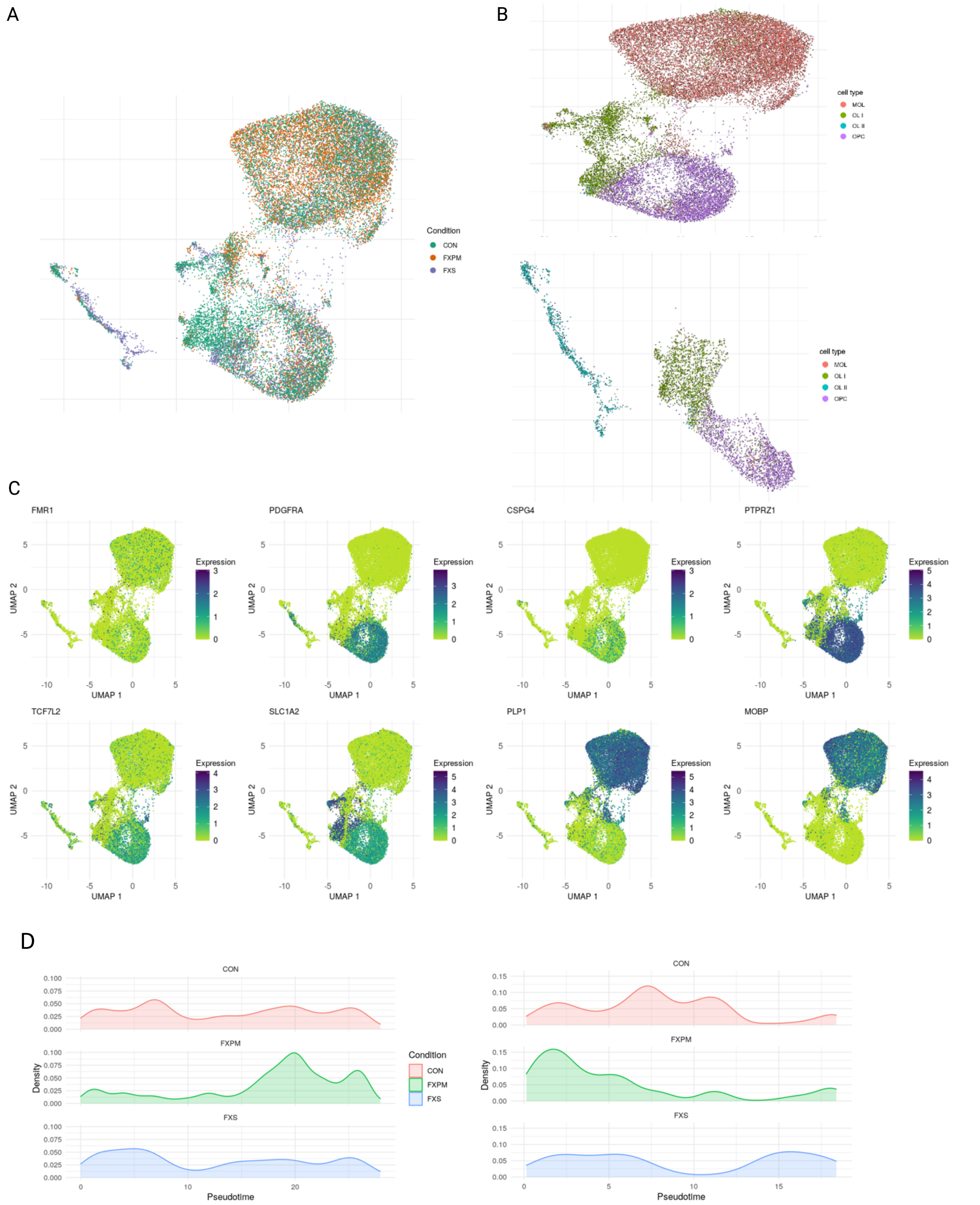

Figure S11. Pseudotime reclustering. **A.** Reclustering demonstrates breakdown of conditions. **B.** Demonstration of two oligodendrocyte branches identified by reclustering. **C.** Markers of oligodendrocyte maturity and FMR1. **D.** Density plot of cell type distribution in branch 1 (left) and branch 2 (right).

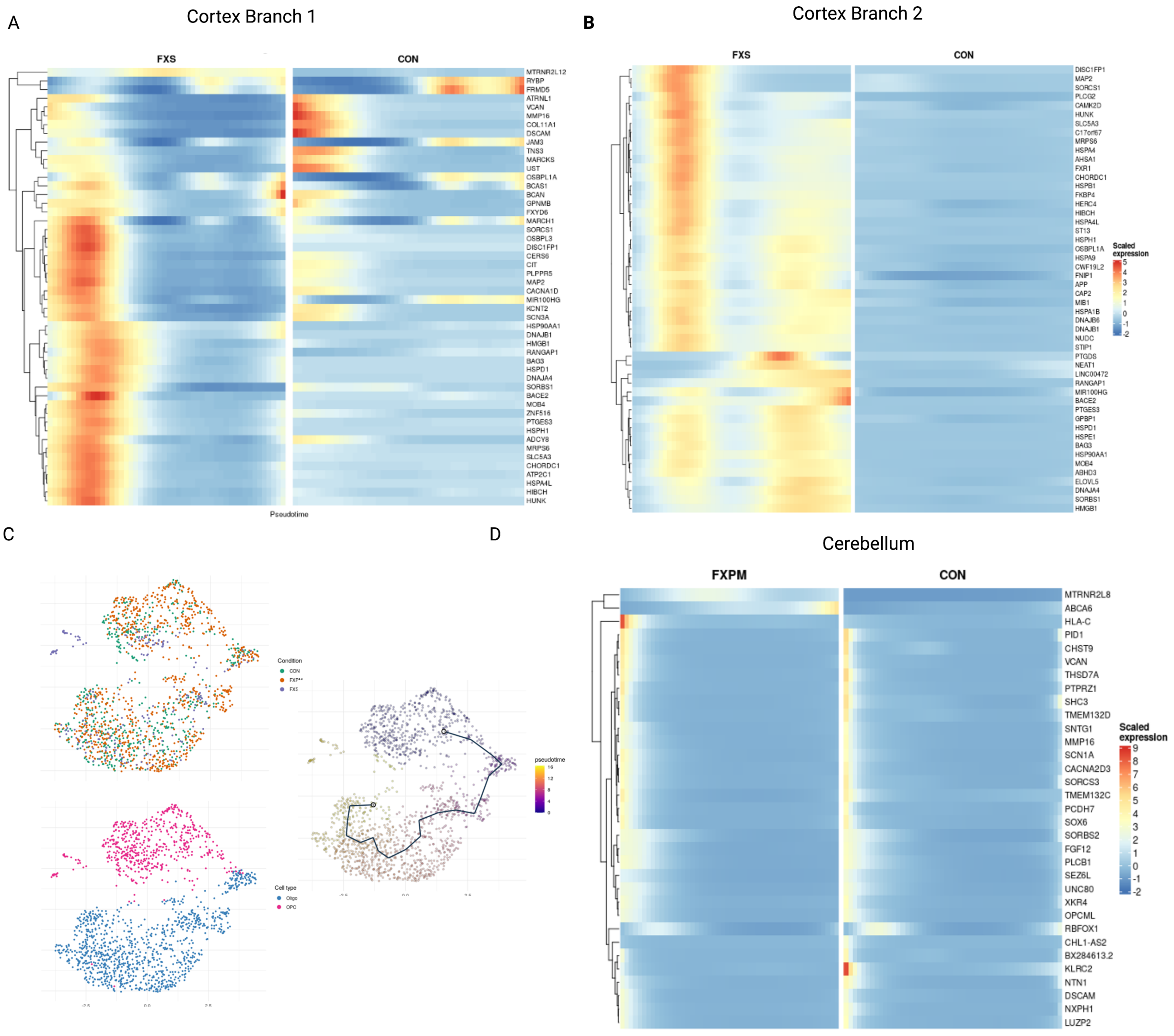

Figure S12 Differential expression in pseudotime. **A.** FXS vs. control comparisons in cortex pseudotime trajectory for branch 1. Heatmap shows top differentially expressed genes (pairwise comparison, Wald statistic). **B.** FXS vs. control comparisons in cortex pseudotime trajectory for branch 2. **C.** Cerebellar pseudotime reclustering broken down by condition (top right) and cell type (bottom right). **D.** Premutation vs. control comparisons in cerebellar pseudotime trajectory. Heatmap shows top differentially expressed genes.

Cell_Type	DEG_Count	Upreg_Count	Downreg_Count
Astro I	1411	768	643
Astro II	1690	792	898
Endo	712	351	361
Inh-PVALB I	296	244	52
Inh-PVALB II	13	11	2
Inh-SST	368	245	123
Inh-SV2C I	494	350	144
Inh-SV2C II	2	1	1
Inh-VIP	332	261	71
L2 3-4	165	100	65
L5 6 I	6	5	1
L5 6 II	0	0	0
Microglia	1443	699	744
MOL	590	395	195
Neu L4 I	239	209	30
Neu L4 II	165	135	30
Neu NRGN	1464	915	549
Neu NTNG2	0	0	0
OL I	1565	924	641
OL II	1936	931	1005
OPC	1907	894	1013

Table S1: Number of differentially expressed genes in frontal cortex clusters FXS vs control. Abbreviations as in Figure 1

Cell_Type	DEG_Count	Upreg_Count	Downreg_Count
Astro I	385	223	162
Astro II	691	371	320
Endo	20	20	0
Inh-PVALB I	280	144	136
Inh-PVALB II	173	64	109
Inh-SST	248	118	130
Inh-SV2C I	138	59	79
Inh-SV2C II	116	55	61
Inh-VIP	193	74	119
L2 3-4	293	151	142
L5 6 I	74	43	31
L5 6 II	38	27	11
Microglia	673	357	316
MOL	421	179	242
Neu L4 I	143	79	64
Neu L4 II	142	78	64
Neu NRGN	1540	900	640
Neu NTNG2	278	179	99
OL I	1557	930	627
OL II	16	11	5
OPC	453	241	212

Table S2: Number of differentially expressed genes in frontal cortex clusters premutation vs control.

Cell_Type	DEG_Count	Upreg_Count	Downreg_Count
Astro I	1518	773	745
Astro II	1986	1157	829
Endo	211	123	88
Inh-PVALB I	673	345	328
Inh-PVALB II	74	41	33
Inh-SST	706	351	355
Inh-SV2C I	687	323	364
Inh-SV2C II	8	3	5
Inh-VIP	704	274	430
L2 3-4	518	257	261
L5 6 I	52	21	31
L5 6 II	2	0	2
Microglia	1447	770	677
MOL	1282	517	765
Neu L4 I	447	192	255
Neu L4 II	350	167	183
Neu NRGN	252	107	145
Neu NTNG2	15	4	11
OL I	1987	1197	790
OL II	781	362	419
OPC	1709	940	769

Table S3: Number of differentially expressed genes in frontal cortex clusters premutation vs FXS.

Cell_Type	DEG_Count	Upreg_Count	Downreg_Count
Astrocyte	867	408	459
Bergmann Glia	1176	667	509
Endothelial	1	1	0
Granule	816	532	284
Interneuron II	216	105	111
Interneuron	149	115	34
Microglia	11	7	4
Oligo	337	221	116
OPC	202	152	50
Purkinje	0	0	0

Table S4: Number of differentially expressed genes in cerebellum clusters FXS vs control.

Cell_Type	DEG_Count	Upreg_Count	Downreg_Count
Astrocyte	209	96	113
Bergmann Glia	226	143	83
Endothelial	3	2	1
Granule	161	75	86
Interneuron II	336	128	208
Interneuron	486	165	321
Microglia	125	57	68
Oligo	123	73	50
OPC	38	29	9
Purkinje	1	1	0

Table S5: Number of differentially expressed genes in cerebellum clusters premutation vs control.

Cell_Type	DEG_Count	Upreg_Count	Downreg_Count
Astrocyte	1345	787	558
Bergmann Glia	1267	617	650
Endothelial	1	1	0
Granule	1024	425	599
Interneuron II	540	172	368
Interneuron	500	240	260
Microglia	126	58	68
Oligo	371	119	252
OPC	360	143	217
Purkinje	0	0	0

Table S6: Number of differentially expressed genes in cerebellum clusters premutation vs FXS.

		Differential expression along the trajectory	Differential expression between conditions
	**Comparison**	**Genes selected (q-value < 0.05, Moran's I > 0.1)**	**Genes with the p.adj < 0.05**
**Branch 1**	FXPM vs CON	1042	383
	FXS vs CON		436
	FXPM vs FXS		695
**Branch 2**	FXPM vs CON	1000	296
	FXS vs CON		741
	FXPM vs FXS		597

Table S7: Number of differentially expressed genes in pseudotime analysis.

motif	TF_highConf	TF_lowConf	NES
transfac_pro__M02771	IRF9 (inferredBy_Orthology).	IRF5 (inferredBy_MotifSimilarity_n_Orthology).	4.95
factorbook__STAT2	STAT1; STAT2 (directAnnotation).	IRF1; IRF2; IRF3; IRF4; IRF5; IRF6; IRF7; IRF8; IRF9 (inferredBy_MotifSimilarity).	4.72
jaspar__MA0517.1	STAT2 (directAnnotation).	IRF1; IRF2; IRF3; IRF4; IRF5; IRF6; IRF7; IRF8; IRF9; STAT1 (inferredBy_MotifSimilarity).	4.19
dbcorrdb__IRF1__ENCSR000EGK_1__m1	IRF1 (directAnnotation).	ELF1; ETV6; GABPA; IRF2; IRF3; IRF4; IRF5; IRF6; IRF7; IRF8; IRF9; POLR2A; PRDM1; RELA; STAT1; STAT2; TBL1XR1 (inferredBy_MotifSimilarity). SPIB (inferredBy_MotifSimilarity_n_Orthology).	4
cisbp__M4635	STAT2 (directAnnotation).	IRF1; IRF2; IRF3; IRF4; IRF5; IRF6; IRF7; IRF8; MTA3; STAT1 (inferredBy_MotifSimilarity).	3.94
cisbp__M5572	IRF3 (directAnnotation).	IRF1; IRF2; IRF4; IRF5; IRF7; IRF8; IRF9; STAT1; STAT2 (inferredBy_MotifSimilarity).	3.93
cisbp__M5580	IRF9 (directAnnotation).	IRF2; IRF3; IRF4; IRF5; IRF7; IRF8 (inferredBy_MotifSimilarity).	3.91
taipale__IRF3_full_NNRRAAAGGAAACCGAAACTN_repr	IRF3 (directAnnotation).	IRF1; IRF2; IRF4; IRF5; IRF7; IRF8; IRF9; STAT1; STAT2 (inferredBy_MotifSimilarity).	3.87
dbcorrdb__STAT1__ENCSR000FAU_1__m1	STAT1 (directAnnotation).	EP300; IKZF1; IRF1; IRF2; IRF3; IRF4; IRF5; IRF6; IRF7; IRF8; IRF9; PRDM1; SPI1; STAT2; STAT3; TBL1XR1 (inferredBy_MotifSimilarity).	3.82
taipale__IRF9_full_AWCGAAACCGAAACY	IRF9 (directAnnotation).	IRF2; IRF3; IRF4; IRF5; IRF7; IRF8 (inferredBy_MotifSimilarity).	3.79
hocomoco__IRF1_MOUSE.H11MO.0.A	IRF1 (inferredBy_Orthology).	BCL11A; EP300; IKZF1; IRF2; IRF3; IRF4; IRF5; IRF6; IRF7; IRF8; IRF9; MTA3; PRDM1; SPI1; SPIB; STAT1; STAT2; STAT3; TBL1XR1 (inferredBy_MotifSimilarity).	3.67
taipale__IRF4_full_NCGAAACCGAAACYN_repr	IRF4 (directAnnotation).	IRF2; IRF3; IRF5; IRF7; IRF8; IRF9 (inferredBy_MotifSimilarity).	3.66
taipale_cyt_meth__IRF8_NYGAAASYGAAASYN_FL	IRF8 (directAnnotation).	IRF1; IRF2; IRF3; IRF4; IRF5; IRF7; IRF9 (inferredBy_MotifSimilarity).	3.58
cisbp__M6309	IRF3 (directAnnotation).	IRF1; IRF2; IRF4; IRF5; IRF6; IRF7; IRF8; IRF9; STAT1; STAT2 (inferredBy_MotifSimilarity).	3.57
cisbp__M6485		SPI1; SPIB; SPIC (inferredBy_MotifSimilarity).	3.56
cisbp__M5579	IRF8 (directAnnotation).	IRF2; IRF3; IRF4; IRF5; IRF7; IRF9 (inferredBy_MotifSimilarity).	3.55
taipale__IRF8_full_NCGAAACCGAAACT	IRF8 (directAnnotation).	IRF2; IRF3; IRF4; IRF5; IRF7; IRF9 (inferredBy_MotifSimilarity).	3.52
tfdimers__MD00026	IRF1; MYB (directAnnotation).		3.51
neph__UW.Motif.0391			3.49
hocomoco__IRF2_HUMAN.H11MO.0.A	IRF2 (directAnnotation).	BCL11A; EP300; IKZF1; IRF1; IRF3; IRF4; IRF5; IRF6; IRF7; IRF8; IRF9; MTA3; PRDM1; SPI1; SPIB; STAT1; STAT2; STAT3; TBL1XR1 (inferredBy_MotifSimilarity).	3.46
cisbp__M6277	HLF (directAnnotation).	ATF2; CEBPB; CEBPD; DBP; NFIL3; TEF (inferredBy_MotifSimilarity). CEBPA; CEBPG (inferredBy_MotifSimilarity_n_Orthology).	3.43
taipale_cyt_meth__IRF9_NYGAAASYGAAACYN_FL_meth	IRF9 (directAnnotation).	IRF1; IRF2; IRF3; IRF4; IRF5; IRF6; IRF7; IRF8 (inferredBy_MotifSimilarity).	3.41
cisbp__M0315	CEBPB (inferredBy_Orthology).	CEBPA; CEBPD; CEBPE; CEBPG; EP300; HLF; PPARGC1A (inferredBy_MotifSimilarity). NFIL3 (inferredBy_MotifSimilarity_n_Orthology).	3.41
homer__GAAAGTGAAAGT_IRF1	IRF1 (directAnnotation).	IRF2; IRF3; IRF4; IRF5; IRF6; IRF7; IRF8; IRF9; PRDM1; RELA; STAT3; TBL1XR1 (inferredBy_MotifSimilarity).	3.4
cisbp__M1271			3.4
taipale_cyt_meth__SPIB_RAWWGMGGAAGTN_FL	SPIB (directAnnotation).	BCL11A; ELF1; ELF2; ELF4; EP300; IKZF1; IRF4; IRF8; SPI1; SPIC (inferredBy_MotifSimilarity).	3.38
cisbp__M5573	IRF4 (directAnnotation).	IRF2; IRF3; IRF5; IRF7; IRF8; IRF9 (inferredBy_MotifSimilarity).	3.38
hocomoco__HLF_HUMAN.H11MO.0.C	HLF (directAnnotation).	ATF2; CEBPB; CEBPD; DBP; NFIL3; TEF (inferredBy_MotifSimilarity). CEBPA; CEBPG (inferredBy_MotifSimilarity_n_Orthology).	3.35
taipale_cyt_meth__IRF5_NYGAAACCGAAACY_FL	IRF5 (directAnnotation).	IRF1; IRF2; IRF3; IRF4; IRF7; IRF8; IRF9 (inferredBy_MotifSimilarity).	3.31
taipale__Hlf_DBD_NRTTACGTAAYN	HLF (inferredBy_Orthology).	ATF2; CEBPE; DBP; NFIL3; TEF (inferredBy_MotifSimilarity). CEBPA (inferredBy_MotifSimilarity_n_Orthology).	3.3
transfac_pro__M00772	IRF1; IRF2; IRF3; IRF4; IRF5; IRF6; IRF7; IRF8 (directAnnotation).	BCL11A; EP300; FOXM1; IRF9; MTA3; PRDM1; RELA; SPI1; STAT1; STAT2; TBL1XR1 (inferredBy_MotifSimilarity).	3.29
dbcorrdb__STAT3__ENCSR000DZV_1__m4	STAT3 (directAnnotation).	IRF4; TBL1XR1 (inferredBy_MotifSimilarity).	3.28
transfac_pro__M06762	ZNF222 (directAnnotation).		3.28
taipale_cyt_meth__IRF2_YGAAASYGAAAS_FL	IRF2 (directAnnotation).	IRF1; IRF3; IRF4; IRF5; IRF7; IRF8; IRF9 (inferredBy_MotifSimilarity).	3.28
transfac_pro__M06268	ZNF260 (directAnnotation).		3.27
taipale_cyt_meth__IRF9_NYGAAASYGAAACYN_FL	IRF9 (directAnnotation).	IRF1; IRF2; IRF3; IRF4; IRF5; IRF7; IRF8 (inferredBy_MotifSimilarity).	3.27
taipale__IRF7_DBD_NCGAAANYGAAACY_repr	IRF7 (directAnnotation).	IRF1; IRF2; IRF4; IRF8; IRF9 (inferredBy_MotifSimilarity). PRDM1 (inferredBy_MotifSimilarity_n_Orthology).	3.26
taipale_cyt_meth__IRF8_NYGAAASYGAAASYN_eDBD	IRF8 (directAnnotation).	IRF1; IRF2; IRF3; IRF4; IRF5; IRF7; IRF9 (inferredBy_MotifSimilarity).	3.26
taipale_cyt_meth__IRF3_NSGAAANSGAAASSGAAASN_FL_repr	IRF3 (directAnnotation).	IRF1; IRF2; IRF4; IRF5; IRF7; IRF8; IRF9; STAT1; STAT2 (inferredBy_MotifSimilarity).	3.25
transfac_pro__M05728		ZNF260 (inferredBy_MotifSimilarity).	3.25
dbcorrdb__POLR2A__ENCSR000ALH_1__m4	POLR2A (directAnnotation).		3.25
transfac_pro__M00699	IRF8 (directAnnotation).	IRF1; IRF2; IRF3; IRF4; IRF5; IRF6; IRF7; IRF9; STAT1; STAT2; TBL1XR1 (inferredBy_MotifSimilarity).	3.24
taipale__IRF8_DBD_NCGAAACCGAAACY	IRF8 (directAnnotation).	IRF2; IRF3; IRF4; IRF5; IRF7; IRF9 (inferredBy_MotifSimilarity).	3.24
taipale_cyt_meth__IRF8_NYGAAASYGAAASYN_FL_meth	IRF8 (directAnnotation).	IRF1; IRF2; IRF3; IRF4; IRF5; IRF6; IRF7; IRF9 (inferredBy_MotifSimilarity).	3.22
cisbp__M6024	HLF (inferredBy_Orthology).	ATF2; CEBPE; DBP; GMEB2; NFIL3; TEF (inferredBy_MotifSimilarity). CEBPA (inferredBy_MotifSimilarity_n_Orthology).	3.22
transfac_pro__M02855	FOXJ3 (inferredBy_Orthology).		3.22
tiffin__TIFDMEM0000011			3.22
cisbp__M6308	IRF2 (directAnnotation).	IRF1; IRF3; IRF4; IRF5; IRF6; IRF7; IRF8; IRF9; PRDM1; STAT2 (inferredBy_MotifSimilarity).	3.21
cisbp__M5578	IRF8 (directAnnotation).	IRF2; IRF3; IRF4; IRF5; IRF7; IRF9 (inferredBy_MotifSimilarity).	3.2
tfdimers__MD00238	BACH1; BACH2; E2F1; MAF; MAFB; MAFF; MAFG; MAFK; NFE2; NFE2L1; NFE2L2; NFE2L3 (directAnnotation).		3.2
idmmpmm__shn	HIVEP1; HIVEP2; HIVEP3; ZNF831 (inferredBy_Orthology).		3.19
dbcorrdb__IRF1__ENCSR000EGU_1__m1	IRF1 (directAnnotation).	BCL11A; EP300; IKZF1; IRF2; IRF3; IRF4; IRF5; IRF6; IRF7; IRF8; IRF9; MTA3; PRDM1; SPI1; STAT1; STAT2; STAT3; TBL1XR1 (inferredBy_MotifSimilarity). SPIB (inferredBy_MotifSimilarity_n_Orthology).	3.19
tfdimers__MD00598	NKX3-2; TAF7; TBP (directAnnotation).		3.19
dbcorrdb__IRF1__ENCSR000EGL_1__m1	IRF1 (directAnnotation).	BCL11A; EP300; IKZF1; IRF2; IRF3; IRF4; IRF5; IRF6; IRF7; IRF8; IRF9; MTA3; PRDM1; SPI1; STAT1; STAT2; STAT3; TBL1XR1 (inferredBy_MotifSimilarity). SPIB (inferredBy_MotifSimilarity_n_Orthology).	3.17
transfac_pro__M07045	IRF1 (directAnnotation).	IRF2; IRF3; IRF4; IRF5; IRF6; IRF7; IRF8; IRF9; PRDM1; STAT1; STAT2 (inferredBy_MotifSimilarity).	3.15
taipale_cyt_meth__IRF4_NYGAAASYGAAASYN_FL	IRF4 (directAnnotation).	IRF1; IRF2; IRF3; IRF5; IRF7; IRF8; IRF9 (inferredBy_MotifSimilarity).	3.15
cisbp__M6307	IRF1 (directAnnotation).	IRF2; IRF3; IRF4; IRF5; IRF6; IRF7; IRF8; IRF9; PRDM1; STAT1; STAT2; STAT3; TBL1XR1 (inferredBy_MotifSimilarity).	3.13
dbcorrdb__STAT2__ENCSR000FBC_1__m1	STAT2 (directAnnotation).	IRF1; IRF2; IRF3; IRF4; IRF5; IRF6; IRF7; IRF8; IRF9; MTA3; SPI1; STAT1; TBL1XR1 (inferredBy_MotifSimilarity).	3.12
transfac_pro__M07432	SOX2 (directAnnotation).	SMAD1; SOX4 (inferredBy_MotifSimilarity).	3.11
cisbp__M4707	STAT1 (directAnnotation).	STAT2 (inferredBy_MotifSimilarity).	3.1
cisbp__M5576	IRF7 (directAnnotation).	IRF1; IRF2; IRF4; IRF8; IRF9 (inferredBy_MotifSimilarity). PRDM1 (inferredBy_MotifSimilarity_n_Orthology).	3.09
transfac_pro__M05305			3.06
transfac_pro__M02880	MAFK (inferredBy_Orthology).		3.05
predrem__nrMotif1219			3.03
taipale__TEF_FL_NRTTACRTAAYN	TEF (directAnnotation).	ATF2; DBP; HLF; NFIL3 (inferredBy_MotifSimilarity). CEBPA; CEBPB (inferredBy_MotifSimilarity_n_Orthology).	3.03
transfac_pro__M08971	SOX15 (directAnnotation).	NANOG; SMAD1 (inferredBy_MotifSimilarity). NANOGP8; SOX14; SOX18; SOX21; SOX4 (inferredBy_MotifSimilarity_n_Orthology).	3.03
cisbp__M5864		ELF2; ELF4; SPI1; SPIB; SPIC (inferredBy_MotifSimilarity). ETV6; ETV7 (inferredBy_MotifSimilarity_n_Orthology).	3.01
transfac_public__M00063	IRF2 (directAnnotation).	IRF1; IRF3; IRF4; IRF5; IRF6; IRF7; IRF8; IRF9 (inferredBy_MotifSimilarity).	3.01
taipale__Dbp_DBD_NRTTACGTAAYN	DBP (inferredBy_Orthology).	ATF2; CEBPB; CEBPD; CEBPE; CEBPG; HLF; NFIL3; TEF (inferredBy_MotifSimilarity). CEBPA (inferredBy_MotifSimilarity_n_Orthology).	3

Table S8: Cortical Microglia output from RCisTarget for premutation vs. control comparison.

motif	TF_highConf	TF_lowConf	NES
jaspar__MA0501.1	NFE2 (directAnnotation).	ARID3A; BACH1; BACH2; EP300; FOS; FOSB; FOSL1; FOSL2; JUN; JUNB; JUND; MAF; MAFB; MAFF; MAFG; MAFK; NFE2L1; NFE2L2; NFE2L3 (inferredBy_MotifSimilarity).	5.34
factorbook__NFE2	NFE2 (directAnnotation).	BACH1; BACH2; FOS; FOSB; FOSL1; FOSL2; JUN; JUNB; JUND; MAF; MAFB; MAFF; MAFG; MAFK; NFE2L1; NFE2L2; NFE2L3 (inferredBy_MotifSimilarity).	5.05
cisbp__M4629	NFE2 (directAnnotation).	BACH1; BACH2; FOSL2; MAF; MAFB; MAFF; MAFG; MAFK; NFE2L1; NFE2L2; NFE2L3 (inferredBy_MotifSimilarity).	4.92
dbcorrdb__BACH1__ENCSR000EGD_1__m1	BACH1 (directAnnotation).	BACH2; EP300; FOS; FOSB; FOSL1; FOSL2; JUN; JUNB; JUND; MAF; MAFB; MAFF; MAFG; MAFK; NFE2; NFE2L1; NFE2L2; NFE2L3 (inferredBy_MotifSimilarity).	4.82
dbcorrdb__NFE2__ENCSR000FAF_1__m1	NFE2 (directAnnotation).	BACH1; BACH2; FOS; FOSB; FOSL1; FOSL2; JUN; JUNB; JUND; MAF; MAFB; MAFF; MAFG; MAFK; NFE2L1; NFE2L2; NFE2L3 (inferredBy_MotifSimilarity).	4.75
homer__AWWNTGCTGAGTCAT_Bach1	BACH1 (directAnnotation).	ARID3A; BACH2; EP300; FOS; FOSB; FOSL1; FOSL2; JUN; JUNB; JUND; MAF; MAFB; MAFF; MAFG; MAFK; NFE2; NFE2L1; NFE2L2; NFE2L3 (inferredBy_MotifSimilarity).	4.65
hocomoco__NFE2_MOUSE.H11MO.0.A		BACH1; BACH2; FOS; FOSB; FOSL1; FOSL2; JUN; JUNB; JUND; MAF; MAFB; MAFF; MAFG; MAFK; NFE2; NFE2L1; NFE2L2; NFE2L3 (inferredBy_MotifSimilarity).	4.64
cisbp__M3617	NFE2 (directAnnotation).	BACH1; BACH2; FOS; FOSB; FOSL1; FOSL2; JUN; JUNB; JUND; MAF; MAFB; MAFF; MAFG; MAFK; NFE2L1; NFE2L2; NRF1 (inferredBy_MotifSimilarity). MAFA (inferredBy_MotifSimilarity_n_Orthology).	4.64
cisbp__M6010	FOXC1 (inferredBy_Orthology).	FOXJ3 (inferredBy_MotifSimilarity). FOXC2; FOXD1; FOXD2; FOXD3; FOXD4; FOXD4L1; FOXD4L3; FOXD4L4; FOXD4L5; FOXD4L6 (inferredBy_MotifSimilarity_n_Orthology).	4.54
hocomoco__NF2L2_HUMAN.H11MO.0.A	NFE2L2 (directAnnotation).	BACH1; BACH2; FOS; FOSB; FOSL1; FOSL2; JUN; JUNB; JUND; MAF; MAFB; MAFF; MAFG; MAFK; NFE2; NFE2L1; NFE2L3 (inferredBy_MotifSimilarity).	4.49
jaspar__MA0150.2	NFE2L2 (inferredBy_Orthology).	BACH1; BACH2; FOS; FOSB; FOSL1; FOSL2; JUN; JUNB; JUND; MAF; MAFB; MAFF; MAFG; MAFK; NFE2; NFE2L1; NFE2L3 (inferredBy_MotifSimilarity).	4.48
taipale__Foxc1_DBD_RTAAAYA_repr	FOXC1 (inferredBy_Orthology).	FOXC2; FOXD1; FOXD2; FOXD3; FOXD4; FOXD4L1; FOXD4L3; FOXD4L4; FOXD4L5; FOXD4L6 (inferredBy_MotifSimilarity_n_Orthology).	4.45
hocomoco__NFE2_HUMAN.H11MO.0.A	NFE2 (directAnnotation).	BACH1; BACH2; FOS; FOSB; FOSL1; FOSL2; JUN; JUNB; JUND; MAF; MAFB; MAFF; MAFG; MAFK; NFE2L1; NFE2L2; NFE2L3 (inferredBy_MotifSimilarity).	4.34
homer__HTGCTGAGTCAT_Nrf2	NFE2L2 (directAnnotation).	BACH1; BACH2; FOS; FOSB; FOSL1; FOSL2; JUN; JUNB; JUND; MAF; MAFB; MAFF; MAFG; MAFK; NFE2; NFE2L1; NFE2L3; TCF12 (inferredBy_MotifSimilarity).	4.34
homer__GATGACTCAGCA_NF-E2	NFE2 (directAnnotation).	BACH1; BACH2; EP300; FOS; FOSB; FOSL1; FOSL2; JUN; JUNB; JUND; MAF; MAFA; MAFB; MAFF; MAFG; MAFK; MEF2A; NFE2L1; NFE2L2; NFE2L3 (inferredBy_MotifSimilarity).	4.34
cisbp__M1966	NFE2L2 (inferredBy_Orthology).	BACH1; BACH2; FOS; FOSB; FOSL1; FOSL2; JUN; JUNB; JUND; MAF; MAFB; MAFF; MAFG; MAFK; NFE2; NFE2L1; NFE2L3 (inferredBy_MotifSimilarity).	4.21
jaspar__MA0555.1		SRF (inferredBy_MotifSimilarity_n_Orthology).	4.15
cisbp__M2348		SRF (inferredBy_MotifSimilarity_n_Orthology).	4.12
cisbp__M6016	FOXJ3 (inferredBy_Orthology).	FOXC2; FOXD2; FOXD3; FOXF1; FOXF2; FOXG1; FOXI1; FOXJ1; FOXJ2; FOXK1; FOXL1; FOXN2; FOXO1; FOXO3; FOXO4; FOXP1; FOXP2; FOXP3; FOXQ1 (inferredBy_MotifSimilarity). FOXD1; FOXD4; FOXD4L1; FOXD4L3; FOXD4L4; FOXD4L5; FOXD4L6; FOXI2; FOXI3; FOXK2; FOXL2; FOXN3; FOXO6; FOXP4 (inferredBy_MotifSimilarity_n_Orthology).	4.09
transfac_pro__M03835	NFE2L2 (directAnnotation).	BACH1; BACH2; FOSL1; MAF; MAFB; MAFF; MAFG; MAFK; NFE2; NFE2L1; NRF1 (inferredBy_MotifSimilarity).	4.08
dbcorrdb__GTF2B__ENCSR000DOE_1__m1	GTF2B (directAnnotation).	GTF2F1; NELFE; POLR2A; TAF1; TBP (inferredBy_MotifSimilarity). TBPL2 (inferredBy_MotifSimilarity_n_Orthology).	4.04
taipale__Foxj3_DBD_RTAAACAA	FOXJ3 (inferredBy_Orthology).	FOXC2; FOXD2; FOXF1; FOXF2; FOXG1; FOXI1; FOXJ1; FOXJ2; FOXK1; FOXL1; FOXN2; FOXO1; FOXO3; FOXO4; FOXP1; FOXP2; FOXP3; FOXQ1 (inferredBy_MotifSimilarity). FOXA2; FOXD1; FOXD3; FOXD4; FOXD4L1; FOXD4L3; FOXD4L4; FOXD4L5; FOXD4L6; FOXI2; FOXI3; FOXK2; FOXL2; FOXN3; FOXO6; FOXP4 (inferredBy_MotifSimilarity_n_Orthology).	3.98
cisbp__M5684	NR3C2 (directAnnotation).	AR; FOXA1; NR3C1 (inferredBy_MotifSimilarity). PGR (inferredBy_MotifSimilarity_n_Orthology).	3.98
swissregulon__hs__TBP.p2	TBP (directAnnotation).	GTF2B; GTF2F1; POLR2A; TAF1 (inferredBy_MotifSimilarity). TBPL2 (inferredBy_MotifSimilarity_n_Orthology).	3.96
cisbp__M1935	NR3C1 (inferredBy_Orthology).	AR; FOXA1; NR3C2; PGR (inferredBy_MotifSimilarity). HSF1; HSF2; HSF4 (inferredBy_MotifSimilarity_n_Orthology).	3.93
cisbp__M5683	NR3C1 (directAnnotation).	AR; FOXA1; NR3C2; PGR (inferredBy_MotifSimilarity).	3.9
taipale__AR_full_RRGWACANNNTGTWCYY	AR (directAnnotation).	NR3C1; NR3C2 (inferredBy_MotifSimilarity).	3.88
cisbp__M4440	NR3C1 (directAnnotation).	AR; FOXA1; PGR (inferredBy_MotifSimilarity). HSF1; HSF2; HSF4 (inferredBy_MotifSimilarity_n_Orthology).	3.88
taipale__NR3C1_DBD_NRGWACANNNTGTWCYN	NR3C1 (directAnnotation).	AR; FOXA1; NR3C2; PGR (inferredBy_MotifSimilarity).	3.88
cisbp__M6142	AR (directAnnotation).	FOXA1; NR3C1; PGR (inferredBy_MotifSimilarity). HSF1; HSF2; HSF4 (inferredBy_MotifSimilarity_n_Orthology).	3.85
swissregulon__hs__NFE2.p2	NFE2 (directAnnotation).	BACH1; BACH2; FOS; FOSB; FOSL1; FOSL2; JUN; JUNB; JUND; MAF; MAFB; MAFF; MAFG; MAFK; NFE2L1; NFE2L2; NRF1 (inferredBy_MotifSimilarity). MAFA (inferredBy_MotifSimilarity_n_Orthology).	3.85
cisbp__M5289	AR (directAnnotation).	NR3C1; NR3C2 (inferredBy_MotifSimilarity).	3.84
yetfasco__YMR042W_1483	SRF (inferredBy_Orthology).		3.83
cisbp__M4010	TBP (directAnnotation).	GTF2B; GTF2F1; POLR2A; TAF1 (inferredBy_MotifSimilarity). TBPL2 (inferredBy_MotifSimilarity_n_Orthology).	3.82
jaspar__MA0108.2		GTF2B; GTF2F1; POLR2A; TAF1; TBP (inferredBy_MotifSimilarity). TBPL2 (inferredBy_MotifSimilarity_n_Orthology).	3.8
factorbook__TBP	POLR2A; TAF1; TBP (directAnnotation).	GTF2B; GTF2F1; NELFE (inferredBy_MotifSimilarity). TBPL2 (inferredBy_MotifSimilarity_n_Orthology).	3.78
taipale__NR3C2_DBD_NRGNACANNNTGTNCYN	NR3C2 (directAnnotation).	AR; FOXA1; NR3C1 (inferredBy_MotifSimilarity). PGR (inferredBy_MotifSimilarity_n_Orthology).	3.77
dbcorrdb__FOXA1__ENCSR000BPX_1__m1	FOXA1 (directAnnotation).	AR; NR3C1; NR3C2; PGR (inferredBy_MotifSimilarity). HSF1; HSF2; HSF4 (inferredBy_MotifSimilarity_n_Orthology).	3.77
homer__CTATAAAAGCSV_TATA-box		POLR2A; TAF1; TBP (inferredBy_MotifSimilarity). TBPL2 (inferredBy_MotifSimilarity_n_Orthology).	3.76
cisbp__M6367	NFE2 (directAnnotation).	BACH1; BACH2; FOS; FOSB; FOSL1; FOSL2; JUN; JUNB; JUND; MAF; MAFB; MAFF; MAFG; MAFK; NFE2L1; NFE2L2; NFE2L3 (inferredBy_MotifSimilarity).	3.75
elemento__GCGCGCC			3.73
transfac_pro__M02263	NFE2L2 (directAnnotation).	BACH1; BACH2; EP300; JUN; MAF; MAFG; MAFK; NFE2; NFE2L1 (inferredBy_MotifSimilarity). MAFF (inferredBy_MotifSimilarity_n_Orthology).	3.64
dbcorrdb__GTF2F1__ENCSR000EHC_1__m1	GTF2F1 (directAnnotation).	GTF2B; NELFE; POLR2A; TAF1; TBP (inferredBy_MotifSimilarity). TBPL2 (inferredBy_MotifSimilarity_n_Orthology).	3.63
cisbp__M5460	FOXL1 (directAnnotation).	FOXC2; FOXD2; FOXF1; FOXF2; FOXG1; FOXI1; FOXJ1; FOXJ2; FOXK1; FOXO1; FOXP2 (inferredBy_MotifSimilarity). FOXC1; FOXD1; FOXD3; FOXD4; FOXD4L1; FOXD4L3; FOXD4L4; FOXD4L5; FOXD4L6; FOXJ3; FOXL2; FOXP1; FOXP3; FOXP4 (inferredBy_MotifSimilarity_n_Orthology).	3.62
transfac_pro__M01240	GTF2IRD1 (directAnnotation).		3.62
stark__STATAWAWRSVVV	TBP; TBPL2 (inferredBy_Orthology).	POLR2A; TAF1 (inferredBy_MotifSimilarity).	3.6
neph__UW.Motif.0510			3.59
swissregulon__hs__FOXP3.p2	FOXP3 (directAnnotation).		3.59
hdpi__RBM35A	ESRP1 (directAnnotation).		3.58
tfdimers__MD00384	IKZF2; STAT1 (directAnnotation).		3.57
neph__UW.Motif.0241			3.54
transfac_pro__M09383			3.53
jaspar__MA0548.1		MEF2A; MEF2C (inferredBy_MotifSimilarity).	3.53
transfac_pro__M02915	SPDEF (inferredBy_Orthology).		3.51
predrem__nrMotif1009			3.51
dbcorrdb__PPARGC1A__ENCSR000EEQ_1__m1	PPARGC1A (directAnnotation).	HSF1; HSF2; HSF4 (inferredBy_MotifSimilarity).	3.5
taipale__FOXL1_full_RTAAAYA	FOXL1 (directAnnotation).	FOXC2; FOXD2; FOXF1; FOXF2; FOXG1; FOXI1; FOXJ1; FOXJ2; FOXK1; FOXO1; FOXP2 (inferredBy_MotifSimilarity). FOXC1; FOXD1; FOXD3; FOXD4; FOXD4L1; FOXD4L3; FOXD4L4; FOXD4L5; FOXD4L6; FOXJ3; FOXL2; FOXP1; FOXP3; FOXP4 (inferredBy_MotifSimilarity_n_Orthology).	3.48
yetfasco__YMR280C_33			3.48
taipale_cyt_meth__NR3C1_NGWACANNNYGTWCN_eDBD	NR3C1 (directAnnotation).	AR; FOXA1; NR3C2 (inferredBy_MotifSimilarity).	3.48
transfac_pro__M09233		HSF1 (inferredBy_MotifSimilarity).	3.47
homer__CCTTTTATAGCC_TATA-Box		GTF2B; GTF2F1; NELFE (inferredBy_MotifSimilarity). TBP (inferredBy_MotifSimilarity_n_Orthology).	3.45

Table S9: Cerebellar Bergmann Glia output from RCisTarget for premutation vs. control comparison. Continued on next page.

cisbp__M5446	FOXD2 (directAnnotation).	FOXA1; FOXA2; FOXA3; FOXC1; FOXC2; FOXD3; FOXF1; FOXF2; FOXG1; FOXI1; FOXJ1; FOXJ2; FOXJ3; FOXK1; FOXL1; FOXO1; FOXO4; FOXO6; FOXP1; FOXP2; FOXP3 (inferredBy_MotifSimilarity). FOXB1; FOXN2; FOXN3; FOXO3; FOXP4 (inferredBy_MotifSimilarity_n_Orthology).	3.45
taipale__Foxk1_DBD_RTAAACA	FOXK1 (inferredBy_Orthology).	FOXC1; FOXC2; FOXD2; FOXD3; FOXF1; FOXF2; FOXG1; FOXI1; FOXJ1; FOXJ2; FOXL1; FOXL2; FOXO1; FOXO4; FOXO6; FOXP1 (inferredBy_MotifSimilarity). FOXD1; FOXD4; FOXD4L1; FOXD4L3; FOXD4L4; FOXD4L5; FOXD4L6; FOXJ3; FOXN2; FOXN3; FOXO3; FOXP2; FOXP3; FOXP4 (inferredBy_MotifSimilarity_n_Orthology).	3.45
transfac_pro__M05297	SOX1 (directAnnotation).	SOX3 (inferredBy_MotifSimilarity_n_Orthology).	3.44
transfac_pro__M09232		HSF1; HSF2 (inferredBy_MotifSimilarity).	3.44
cisbp__M6019	FOXK1 (inferredBy_Orthology).	FOXC1; FOXC2; FOXD2; FOXD3; FOXF1; FOXF2; FOXG1; FOXI1; FOXJ1; FOXJ2; FOXL1; FOXL2; FOXO1; FOXO4; FOXO6; FOXP1; FOXP3 (inferredBy_MotifSimilarity). FOXD1; FOXD4; FOXD4L1; FOXD4L3; FOXD4L4; FOXD4L5; FOXD4L6; FOXJ3; FOXN2; FOXN3; FOXO3; FOXP2; FOXP4 (inferredBy_MotifSimilarity_n_Orthology).	3.43
cisbp__M3450	IKZF1 (directAnnotation).		3.42
cisbp__M5985	AR (inferredBy_Orthology).	NR3C1; NR3C2 (inferredBy_MotifSimilarity).	3.42
swissregulon__hs__NFE2L2.p2	NFE2L2 (directAnnotation).	BACH1; BACH2; FOS; FOSB; FOSL1; FOSL2; JUN; JUNB; JUND; MAFF; MAFG; MAFK; NFE2 (inferredBy_MotifSimilarity). ATF3 (inferredBy_MotifSimilarity_n_Orthology).	3.41
cisbp__M0750	FOXP1; FOXP2; FOXP3; FOXP4 (inferredBy_Orthology).	FOXA1; FOXA2; FOXB1; FOXC1; FOXC2; FOXD1; FOXD2; FOXG1; FOXI1; FOXJ2; FOXJ3; FOXK1; FOXL1; FOXO1; FOXO3; FOXO4; FOXO6; FOXQ1 (inferredBy_MotifSimilarity). FOXD3; FOXD4; FOXD4L1; FOXD4L3; FOXD4L4; FOXD4L5; FOXD4L6; FOXF1; FOXF2; FOXN2; FOXN3; SRF (inferredBy_MotifSimilarity_n_Orthology).	3.41
swissregulon__sacCer__YDR520C			3.4
cisbp__M4681	BACH1 (directAnnotation).	BACH2; JUN; MAF; MAFA; MAFB; MAFF; MAFG; MAFK; NFE2; NFE2L1; NFE2L2; NFE2L3 (inferredBy_MotifSimilarity).	3.4
taipale__FOXJ2_DBD_RTAAACAA	FOXJ2 (directAnnotation).	FOXC2; FOXD2; FOXD3; FOXF1; FOXF2; FOXG1; FOXI1; FOXJ1; FOXJ3; FOXK1; FOXL1; FOXO1; FOXO3; FOXO4; FOXP1; FOXP2; FOXP3; FOXQ1 (inferredBy_MotifSimilarity). FOXD1; FOXD4; FOXD4L1; FOXD4L3; FOXD4L4; FOXD4L5; FOXD4L6; FOXI2; FOXI3; FOXK2; FOXL2; FOXN2; FOXN3; FOXO6; FOXP4 (inferredBy_MotifSimilarity_n_Orthology).	3.39
predrem__nrMotif334			3.39
cisbp__M6260	NR3C1 (directAnnotation).	AR; FOXA1; PGR (inferredBy_MotifSimilarity). HSF1; HSF2; HSF4 (inferredBy_MotifSimilarity_n_Orthology).	3.38
transfac_pro__M09240		HSF1 (inferredBy_MotifSimilarity).	3.36
cisbp__M2341		MEF2A; MEF2C (inferredBy_MotifSimilarity).	3.36
cisbp__M2288	HSF1 (directAnnotation).	HOXB2; HSF2; HSF4 (inferredBy_MotifSimilarity).	3.36
taipale_cyt_meth__HOXA13_NCCAATAAAAM_eDBD_repr	HOXA13 (directAnnotation).	HOXB13; HOXC13; HOXD13 (inferredBy_MotifSimilarity).	3.35
elemento__CGAGCGC			3.35
swissregulon__hs__HSF1_2.p2	HSF1; HSF2 (directAnnotation).	HSF4 (inferredBy_MotifSimilarity_n_Orthology).	3.35
homer__NAGAACAGNCTGTTCT_GRE		AR; FOXA1; NR3C1; NR3C2; PGR (inferredBy_MotifSimilarity). HSF1; HSF2; HSF4 (inferredBy_MotifSimilarity_n_Orthology).	3.33
taipale__Ar_DBD_RRGNACANNNTGTNCYY_repr	AR (inferredBy_Orthology).	NR3C1; NR3C2 (inferredBy_MotifSimilarity).	3.31
elemento__CGCGCGC			3.3
cisbp__M5454	FOXJ2 (directAnnotation).	FOXC2; FOXD2; FOXD3; FOXF1; FOXF2; FOXG1; FOXI1; FOXJ1; FOXJ3; FOXK1; FOXL1; FOXN2; FOXO1; FOXO3; FOXO4; FOXP1; FOXP2; FOXP3; FOXQ1 (inferredBy_MotifSimilarity). FOXA2; FOXD1; FOXD4; FOXD4L1; FOXD4L3; FOXD4L4; FOXD4L5; FOXD4L6; FOXI2; FOXI3; FOXK2; FOXL2; FOXN3; FOXO6; FOXP4 (inferredBy_MotifSimilarity_n_Orthology).	3.29
hocomoco__BACH2_HUMAN.H11MO.0.A	BACH2 (directAnnotation).	BACH1; EP300; FOS; FOSB; FOSL1; FOSL2; JUN; JUNB; JUND; MAF; MAFB; MAFF; MAFG; MAFK; NFE2; NFE2L1; NFE2L2; NFE2L3; NRF1 (inferredBy_MotifSimilarity). ATF3 (inferredBy_MotifSimilarity_n_Orthology).	3.27
transfac_pro__M07568		HSF1; HSF2; HSF4 (inferredBy_MotifSimilarity).	3.27
transfac_pro__M01128			3.27
taipale_tf_pairs__HOXB2_ESRRB_TAATKRNNNNNNNAAGGTCA_CAP_repr	ESRRB; HOXB2 (directAnnotation).		3.26
dbcorrdb__GTF2F1__ENCSR000EBP_1__m1	GTF2F1 (directAnnotation).	GTF2B; POLR2A; TAF1; TBP (inferredBy_MotifSimilarity). TBPL2 (inferredBy_MotifSimilarity_n_Orthology).	3.26
tfdimers__MD00429	ARID3A; IKZF2 (directAnnotation).		3.24
tfdimers__MD00082	GATA1; GATA2; GATA3; GATA4; GATA5; GATA6; ZNF217 (directAnnotation).		3.23
taipale_tf_pairs__HOXB2_SOX15_ACAAWRSNNNNYMATTA_CAP_repr	HOXB2; SOX15 (directAnnotation).		3.23
factorbook__HSF1	HSF1 (directAnnotation).	HOXB2; HSF2; HSF4 (inferredBy_MotifSimilarity).	3.19
cisbp__M2351		MEF2A; MEF2C (inferredBy_MotifSimilarity).	3.19
hocomoco__ANDR_MOUSE.H11MO.0.A	AR (inferredBy_Orthology).	FOXA1; NR3C1; NR3C2; PGR (inferredBy_MotifSimilarity). HSF1; HSF2; HSF4 (inferredBy_MotifSimilarity_n_Orthology).	3.19
taipale__AR_DBD_RRGWACANNNTGTWCYY	AR (directAnnotation).	NR3C1; NR3C2 (inferredBy_MotifSimilarity).	3.17
dbcorrdb__HA-E2F1__ENCSR000EVM_1__m1	E2F1 (directAnnotation).	BRF1; CEBPB; CREB1; CTCF; E2F6; ESRRA; EZH2; HDAC2; MAX; MXI1; MYC; NELFE; NRF1; POLR2A; RAD21; RBBP5; SMARCA4; SREBF1; SREBF2; XRCC4; ZMIZ1; ZNF274 (inferredBy_MotifSimilarity).	3.17
dbcorrdb__POLR2A__ENCSR000AOJ_1__m12	POLR2A (directAnnotation).		3.17
hdpi__PSMA6	PSMA6 (directAnnotation).		3.16
jaspar__MA0591.1	MAFK (inferredBy_Orthology).	BACH1; BACH2; FOS; FOSB; FOSL1; FOSL2; JUN; JUNB; JUND; MAF; MAFB; MAFF; MAFG; MEF2A; NFE2; NFE2L1; NFE2L2; NFE2L3; SMARCC1 (inferredBy_MotifSimilarity).	3.16
predrem__nrMotif1986			3.15
transfac_pro__M07100	HSF1 (directAnnotation).	HOXB2; HSF2; HSF4 (inferredBy_MotifSimilarity).	3.15
dbcorrdb__NR3C1__ENCSR000BHE_1__m1	NR3C1 (directAnnotation).	AR; FOXA1; PGR (inferredBy_MotifSimilarity).	3.15
homer__AGAACAGNCTGTTCTT_ARE	AR (directAnnotation).	FOXA1; NR3C1; PGR (inferredBy_MotifSimilarity).	3.14
transfac_pro__M09216			3.14
transfac_pro__M03573	FOXF1 (directAnnotation).		3.13
hocomoco__PAX6_HUMAN.H11MO.0.C	PAX6 (directAnnotation).	PAX1; PAX2; PAX5; PAX9 (inferredBy_MotifSimilarity).	3.13
hocomoco__ELK3_HUMAN.H11MO.0.D	ELK3 (directAnnotation).	ELF1; ELK4; ERG; FLI1 (inferredBy_MotifSimilarity).	3.12
dbcorrdb__POLR2A__ENCSR000ALH_1__m1	POLR2A (directAnnotation).	GTF2B; GTF2F1; TAF1; TBP (inferredBy_MotifSimilarity).	3.12
cisbp__M0747		FOXA1; FOXA2; FOXA3; FOXB1; FOXC1; FOXC2; FOXD2; FOXD3; FOXF2; FOXI1; FOXK1; FOXL1; FOXN2; FOXO1; FOXO3; FOXO4; FOXP3 (inferredBy_MotifSimilarity). FOXD1; FOXD4; FOXD4L1; FOXD4L3; FOXD4L4; FOXD4L5; FOXD4L6; FOXF1; FOXG1; FOXJ1; FOXL2; FOXP1; FOXP2; FOXP4; SRF (inferredBy_MotifSimilarity_n_Orthology).	3.09
transfac_pro__M06871	ZNF224 (directAnnotation).		3.09
cisbp__M0715		FOXC2; FOXD2; FOXF1; FOXF2; FOXG1; FOXI1; FOXJ1; FOXJ2; FOXJ3; FOXK1; FOXL1; FOXO1; FOXO4; FOXP1; FOXP2 (inferredBy_MotifSimilarity). FOXD1; FOXD3; FOXD4; FOXD4L1; FOXD4L3; FOXD4L4; FOXD4L5; FOXD4L6; FOXI2; FOXI3; FOXK2; FOXL2; FOXN2; FOXN3; FOXO3; FOXO6; FOXP3; FOXP4; FOXQ1 (inferredBy_MotifSimilarity_n_Orthology).	3.08
transfac_pro__M08998	NR3C1 (inferredBy_Orthology).	AR; FOXA1; NR3C2; PGR (inferredBy_MotifSimilarity).	3.08
cisbp__M6360	NFE2L2 (directAnnotation).	BACH1; BACH2; FOSL1; MAF; MAFB; MAFF; MAFG; MAFK; NFE2; NFE2L1; NRF1 (inferredBy_MotifSimilarity). JUN (inferredBy_MotifSimilarity_n_Orthology).	3.08
tfdimers__MD00186	NFATC3; SPI1 (directAnnotation).		3.07
cisbp__M1825			3.07
taipale__Pou2f2_DBD_NWTRMATATKYAWN	POU2F2 (inferredBy_Orthology).	POU2F1; POU2F3; POU3F3; POU5F1B (inferredBy_MotifSimilarity). POU3F1; POU3F2 (inferredBy_MotifSimilarity_n_Orthology).	3.07
flyfactorsurvey__D19B_F10-12_SANGER_5_FBgn0022699			3.06
taipale__FOXD2_DBD_RTAAAYA	FOXD2 (directAnnotation).	ARID3A; FOXA1; FOXA2; FOXA3; FOXB1; FOXC1; FOXC2; FOXD3; FOXF1; FOXF2; FOXG1; FOXI1; FOXJ1; FOXJ2; FOXJ3; FOXK1; FOXL1; FOXN2; FOXO1; FOXO4; FOXO6; FOXP1; FOXP2; FOXP3 (inferredBy_MotifSimilarity). FOXL2; FOXN3; FOXO3; FOXP4 (inferredBy_MotifSimilarity_n_Orthology).	3.05
transfac_pro__M00641	HSF1 (directAnnotation).		3.05
cisbp__M6063	POU2F2 (inferredBy_Orthology).	POU2F1; POU2F3; POU3F3; POU5F1B (inferredBy_MotifSimilarity). POU3F1; POU3F2 (inferredBy_MotifSimilarity_n_Orthology).	3.04
transfac_pro__M07265	NFE2L2 (directAnnotation).	BACH1; BACH2; FOSL1; JUN; MAF; MAFB; MAFF; MAFG; MAFK; NFE2; NFE2L1; NRF1; SMARCC1 (inferredBy_MotifSimilarity). ATF3 (inferredBy_MotifSimilarity_n_Orthology).	3.04
hocomoco__SOX10_MOUSE.H11MO.1.A	SOX10 (inferredBy_Orthology).	NANOG; SMAD1; SOX2; SOX3; SOX4 (inferredBy_MotifSimilarity). NANOGP8; SOX11; SOX6; SOX9 (inferredBy_MotifSimilarity_n_Orthology).	3.04
transfac_public__M00086	IKZF1 (directAnnotation).		3.03
transfac_pro__M09338			3.03
cisbp__M0726	FOXJ3 (inferredBy_Orthology).	AR; FOXA1; FOXA3; FOXC2; FOXD1; FOXD2; FOXG1; FOXJ2; FOXL1; FOXO1; FOXO3; FOXO4; FOXP1; FOXP2 (inferredBy_MotifSimilarity). FOXA2; FOXD3; FOXD4; FOXD4L1; FOXD4L3; FOXD4L4; FOXD4L5; FOXD4L6; FOXF1; FOXF2; FOXJ1; FOXK1; FOXN2; FOXN3; FOXP3; FOXP4 (inferredBy_MotifSimilarity_n_Orthology).	3.03
predrem__nrMotif2134			3.03
hdpi__NME1	NME1 (directAnnotation).		3.02
transfac_pro__M07512			3
dbcorrdb__ZZZ3__ENCSR000DNQ_1__m1	ZZZ3 (directAnnotation).		3

Table S9 Continued: Cerebellar Bergmann Glia output from RCisTarget for premutation vs. control comparison.

CBL	Estimated Number of Cells	Mean Reads per Cell	Median Genes per Cell	Number of Reads	Valid Barcodes	Sequencing Saturation	Q30 Bases in Barcode	Q30 Bases in RNA Read	Q30 Bases in UMI	Reads Mapped to Genome
1793	4,701	56,278	3,524	264,564,211	95.40%	60.10%	91.80%	93.00%	76.50%	95.70%
5497	5,566	70,949	3,126	394,904,625	97.50%	80.60%	91.70%	87.30%	91.50%	95.10%
5541	5,360	90,127	3,704	483,082,020	96.40%	78.10%	91.50%	87.50%	91.40%	94.40%
5657	5,764	92,365	3,372	532,394,454	97.30%	80.20%	95.40%	92.40%	95.10%	95.90%
AN10723	6,372	46,761	2,571	297,964,738	97.50%	75.90%	95.90%	91.80%	95.40%	93.30%
4806	4,645	90,352	2,154	419,682,889	97.10%	88.30%	90.70%	86.00%	90.80%	93.70%
5319	5,674	71,804	3,746	407,415,252	96.60%	71.30%	91.60%	87.60%	91.50%	94.80%
4555	3,944	70,778	2,652	279,149,167	97.60%	82.00%	95.90%	92.00%	95.50%	92.30%
4664	4,741	47,939	2,737	227,279,509	97.40%	71.30%	91.00%	86.30%	90.80%	94.20%
5006	4,670	35,863	3,107	167,481,681	96.60%	55.80%	91.20%	86.50%	91.00%	94.40%
5212	5,070	49,632	3,002	251,631,846	95.80%	61.40%	91.90%	93.50%	76.40%	96.10%
5746	5,166	41,978	2,909	216,860,497	96.00%	60.10%	92.60%	94.00%	77.70%	96.50%
4751	4,821	117,130	3,534	564,684,721	97.20%	85.40%	95.30%	92.40%	95.10%	95.70%

CTX	Estimated Number of Cells	Mean Reads per Cell	Median Genes per Cell	Number of Reads	Valid Barcodes	Sequencing Saturation	Q30 Bases in Barcode	Q30 Bases in RNA Read	Q30 Bases in UMI	Reads Mapped to Genome
1793	3,073	74,963	3,159	230,363,514	95.70%	65.50%	98.40%	91.80%	98.40%	95.70%
5408	4,389	59,487	3,028	261,092,263	97.60%	67.40%	95.90%	91.90%	95.50%	94.10%
5497	5,557	72,214	3,029	401,292,261	97.20%	76.50%	90.80%	85.90%	90.80%	94.50%
5541	5,077	81,152	3,992	412,009,797	96.80%	67.20%	91.60%	87.90%	91.50%	94.70%
5657	4,897	103,724	4,178	507,936,830	97.50%	66.70%	95.40%	92.20%	95.10%	95.90%
4806	4,517	88,308	2,796	398,886,965	97.00%	81.90%	90.80%	85.80%	90.80%	94.10%
5319	5,333	87,892	3,525	468,728,034	96.90%	70.30%	91.60%	87.70%	91.50%	94.70%
5746 (BA22)	2,393	109,360	3,954	261,698,719	96.00%	59.80%	91.70%	93.50%	76.30%	94.90%
4555	2,739	112,219	3,500	307,367,917	97.70%	78.60%	95.90%	91.60%	95.50%	91.80%
4664	2,542	80,464	2,641	204,540,615	97.30%	75.60%	91.10%	86.20%	90.90%	93.90%
4751	3,443	177,604	4,142	611,491,905	97.20%	84.90%	95.30%	92.20%	95.10%	95.40%
5006	3,956	44,910	2,846	177,663,948	97.00%	44.90%	91.00%	86.10%	90.80%	93.70%
5529	4,233	69,880	4,048	295,802,824	95.50%	43.00%	91.90%	93.60%	76.60%	96.20%

Table S10: Metrics raw output from Cell Ranger pipeline for samples.

Supplemental Information File 1. Linear regression was used to assess the effect of premutation condition and age on premutation and control groups using the equation

$y=\beta_0+\beta_1 x_1+\beta_2 x_2$

Nuclei Cluster % $= \beta_0 + \beta_1$*condition $+ \beta_2$*age

Cortex

Excitatory Neurons

Parameter estimates	Variable	Estimate	Standard error	95% CI (asymptotic)	|t|	P value	P value summary
β0	Intercept	-0.9146	6.818	-17.04 to 15.21	0.1341	0.8971	ns
β1	Group[PM]	-1.886	6.698	-17.72 to 13.95	0.2815	0.7864	ns
β2	Age	0.2333	0.1024	-0.008771 to 0.4754	2.279	0.0567	ns

Inhibitory Neurons

Parameter estimates	Variable	Estimate	Standard error	95% CI (asymptotic)	|t|	P value	P value summary
β0	Intercept	7.891	3.971	-1.500 to 17.28	1.987	0.0873	ns
β1	Group[PM]	-6.573	3.901	-15.80 to 2.652	1.685	0.1359	ns
β2	Age	0.1398	0.05963	-0.001238 to 0.2808	2.344	0.0516	ns

Astrocyte I

Parameter estimates	Variable	Estimate	Standard error	95% CI (asymptotic)	|t|	P value	P value summary
β0	Intercept	18.84	2.779	12.26 to 25.41	6.778	0.0003	***
β1	Group[PM]	-9.903	2.730	-16.36 to -3.447	3.627	0.0084	**
β2	Age	-0.05304	0.04173	-0.1517 to 0.04563	1.271	0.2443	ns

Astrocyte II

Parameter estimates	Variable	Estimate	Standard error	95% CI (asymptotic)	|t|	P value	P value summary

β0	Intercept	7.704	1.198	4.871 to 10.54	6.431	0.0004	***
β1	Group[PM]	-3.501	1.177	-6.284 to -0.7179	2.975	0.0207	*
β2	Age	-0.02099	0.01799	-0.06352 to 0.02155	1.167	0.2815	ns

Mature Oligodendrocytes

Parameter estimates	Variable	Estimate	Standard error	95% CI (asymptotic)	|t|	P value	P value summary
β0	Intercept	5.296	11.57	-22.06 to 32.66	0.4577	0.6610	ns
β1	Group[PM]	20.44	11.37	-6.435 to 47.32	1.799	0.1151	ns
β2	Age	0.2323	0.1737	-0.1785 to 0.6431	1.337	0.2231	ns

OPC

Parameter estimates	Variable	Estimate	Standard error	95% CI (asymptotic)	|t|	P value	P value summary
β0	Intercept	21.66	2.683	15.32 to 28.01	8.074	<0.0001	****
β1	Group[PM]	4.463	2.636	-1.770 to 10.70	1.693	0.1343	ns
β2	Age	-0.2454	0.04029	-0.3407 to -0.1501	6.091	0.0005	***

Microglia

Parameter estimates	Variable	Estimate	Standard error	95% CI (asymptotic)	|t|	P value	P value summary
β0	Intercept	21.57	3.523	13.24 to 29.90	6.124	0.0005	***
β1	Group[PM]	-0.6021	3.461	-8.785 to 7.581	0.1740	0.8668	ns
β2	Age	-0.1671	0.05289	-0.2921 to -0.04198	3.158	0.0160	*

Endothelial

Parameter estimates	Variable	Estimate	Standard error	95% CI (asymptotic)	|t|	P value	P value summary
β0	Intercept	0.7394	0.2675	0.1070 to 1.372	2.765	0.0279	*
β1	Group[PM]	-0.3206	0.2628	-0.9419 to 0.3007	1.220	0.2619	ns

β2	Age	-0.003812	0.004016	-0.01331 to 0.005685	0.9491	0.3742	ns

Inhibitory Neuron Density (Inhibitory/Total Neuron percentage)

Parameter estimates	Variable	Estimate	Standard error	95% CI (asymptotic)	|t|	P value	P value summary
β0	Intercept	0.7645	0.05505	0.6343 to 0.8947	13.89	<0.0001	****
β1	Group[PM]	-0.05218	0.05408	-0.1801 to 0.07569	0.9650	0.3667	ns
β2	Age	-0.002581	0.0008265	-0.004535 to -0.0006263	3.122	0.0168	*

<u>Cerebellum</u>

Purkinje

Parameter estimates	Variable	Estimate	Standard error	95% CI (asymptotic)	|t|	P value	P value summary
β0	Intercept	0.1047	0.1228	-0.1785 to 0.3880	0.8525	0.4187	ns
β1	Condition[PM]	-0.1568	0.08565	-0.3543 to 0.04070	1.831	0.1045	ns
β2	Age	0.001808	0.001735	-0.002194 to 0.005810	1.042	0.3279	ns

Bergmann Glia

Parameter estimates	Variable	Estimate	Standard error	95% CI (asymptotic)	|t|	P value	P value summary
β0	Intercept	4.594	1.552	1.014 to 8.174	2.959	0.0182	*
β1	Condition[PM]	2.378	1.082	-0.1182 to 4.874	2.197	0.0593	ns
β2	Age	-0.03712	0.02193	-0.08769 to 0.01346	1.692	0.1290	ns

Granule

Parameter estimates	Variable	Estimate	Standard error	95% CI (asymptotic)	|t|	P value	P value summary
β0	Intercept	87.25	4.912	75.92 to 98.57	17.76	<0.0001	****
β1	Condition[PM]	-6.366	3.425	-14.26 to 1.531	1.859	0.1001	ns
β2	Age	0.05032	0.06939	-0.1097 to 0.2103	0.7252	0.4890	ns

OPC

Parameter estimates	Variable	Estimate	Standard error	95% CI (asymptotic)	|t|	P value	P value summary
β0	Intercept	1.564	0.5186	0.3680 to 2.760	3.016	0.0167	*
β1	Condition[PM]	0.6912	0.3616	-0.1427 to 1.525	1.911	0.0923	ns
β2	Age	-0.01629	0.007327	-0.03319 to 0.0006017	2.224	0.0568	ns

Astrocyte

Parameter estimates	Variable	Estimate	Standard error	95% CI (asymptotic)	|t|	P value	P value summary
β0	Intercept	1.084	0.3860	0.1943 to 1.974	2.809	0.0229	*
β1	Condition[PM]	0.01825	0.2691	-0.6023 to 0.6388	0.06780	0.9476	ns
β2	Age	-0.002173	0.005453	-0.01475 to 0.01040	0.3984	0.7008	ns

Oligodendrocyte

Parameter estimates	Variable	Estimate	Standard error	95% CI (asymptotic)	|t|	P value	P value summary
β0	Intercept	1.382	0.5098	0.2067 to 2.558	2.711	0.0266	*
β1	Condition[PM]	0.5364	0.3554	-0.2833 to 1.356	1.509	0.1697	ns
β2	Age	-0.0008881	0.007202	-0.01750 to 0.01572	0.1233	0.9049	ns

Microglia

Parameter estimates	Variable	Estimate	Standard error	95% CI (asymptotic)	|t|	P value	P value summary
β0	Intercept	1.069	0.4512	0.02846 to 2.109	2.369	0.0453	*
β1	Condition[PM]	0.1759	0.3146	-0.5495 to 0.9014	0.5592	0.5913	ns
β2	Age	-0.004652	0.006375	-0.01935 to 0.01005	0.7298	0.4863	ns

Interneuron I

Parameter estimates	Variable	Estimate	Standard error	95% CI (asymptotic)	|t|	P value	P value summary
β0	Intercept	1.028	1.057	-1.409 to 3.466	0.9728	0.3591	ns
β1	Condition[PM]	1.007	0.7371	-0.6927 to 2.707	1.366	0.2090	ns
β2	Age	0.01240	0.01494	-0.02204 to 0.04684	0.8301	0.4306	ns

Interneuron II

Parameter estimates	Variable	Estimate	Standard error	95% CI (asymptotic)	|t|	P value	P value summary
β0	Intercept	1.708	1.187	-1.030 to 4.446	1.439	0.1882	ns
β1	Condition[PM]	1.837	0.8279	-0.07257 to 3.746	2.218	0.0573	ns
β2	Age	-0.005094	0.01677	-0.04378 to 0.03359	0.3037	0.7691	ns

Supplemental Information File 2

Cerebellar top 20 terms for enriched biological processes (BP) and Kyoto Encyclopedia of Genes and Genomes (KEGG) terms for each cellular population with each condition comparison, potential terms of particular interest highlighted with red box.

Astrocyte

GO_BP

Top 20

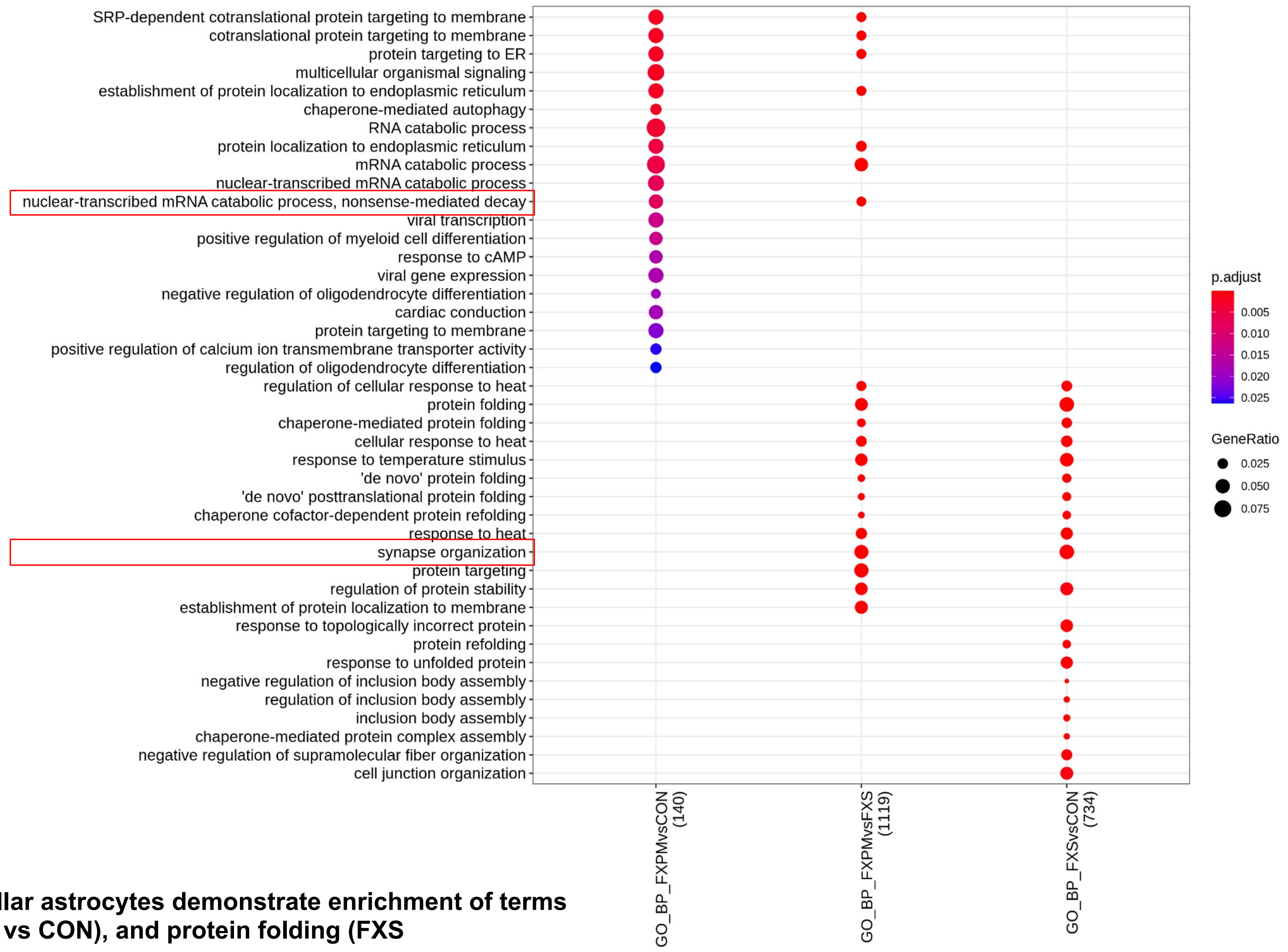

GO Biological Processes for cerebellar astrocytes demonstrate enrichment of terms implicated in mRNA catabolism (PM vs CON), and protein folding (FXS comparisons).

Astrocyte

KEGG

Top 20

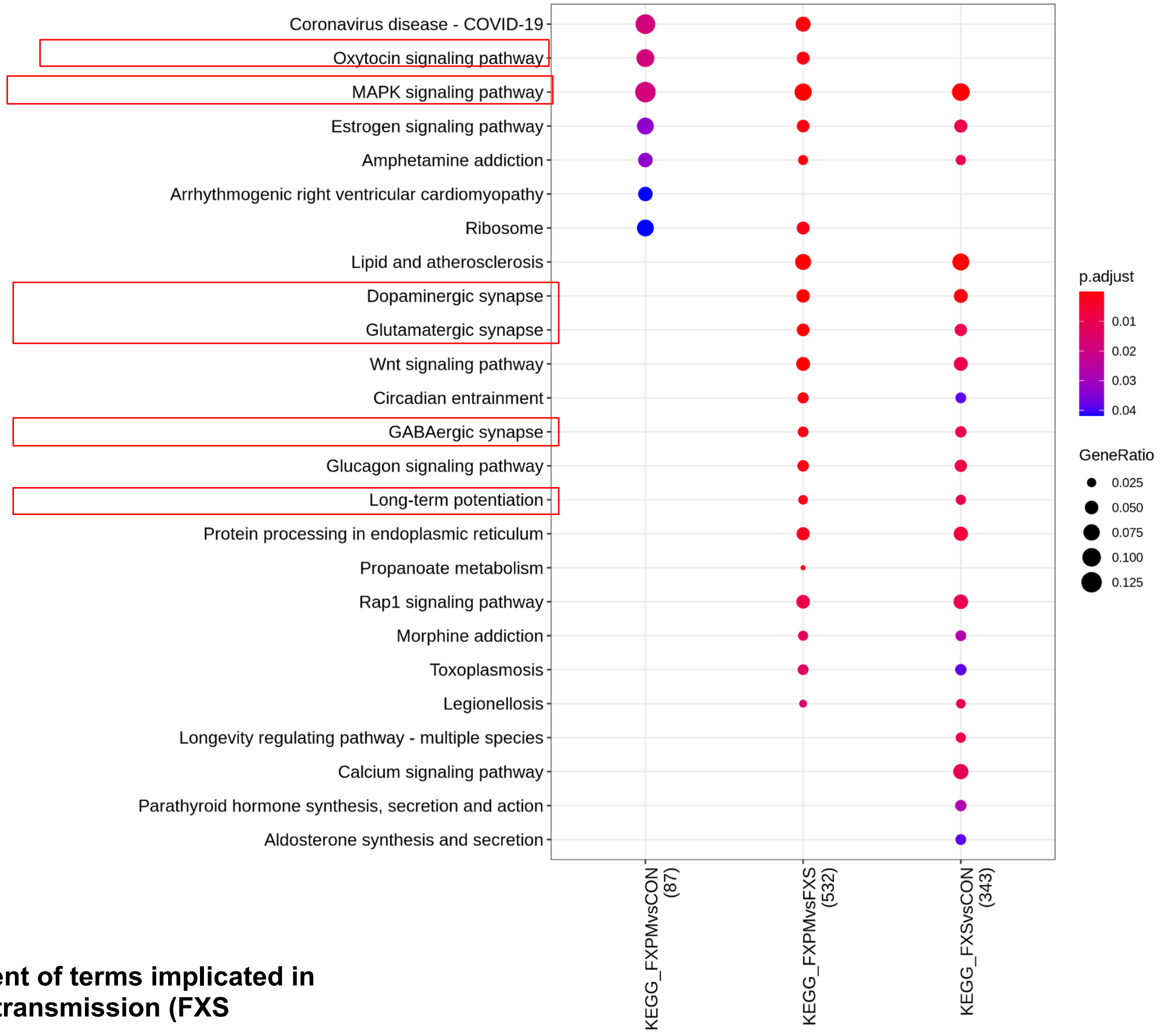

KEGG for cerebellar astrocytes demonstrate enrichment of terms implicated in MAPK signaling (PM & FXS comparisons), and neurotransmission (FXS comparisons).

Bergmann Glia

GO_BP

Top 20

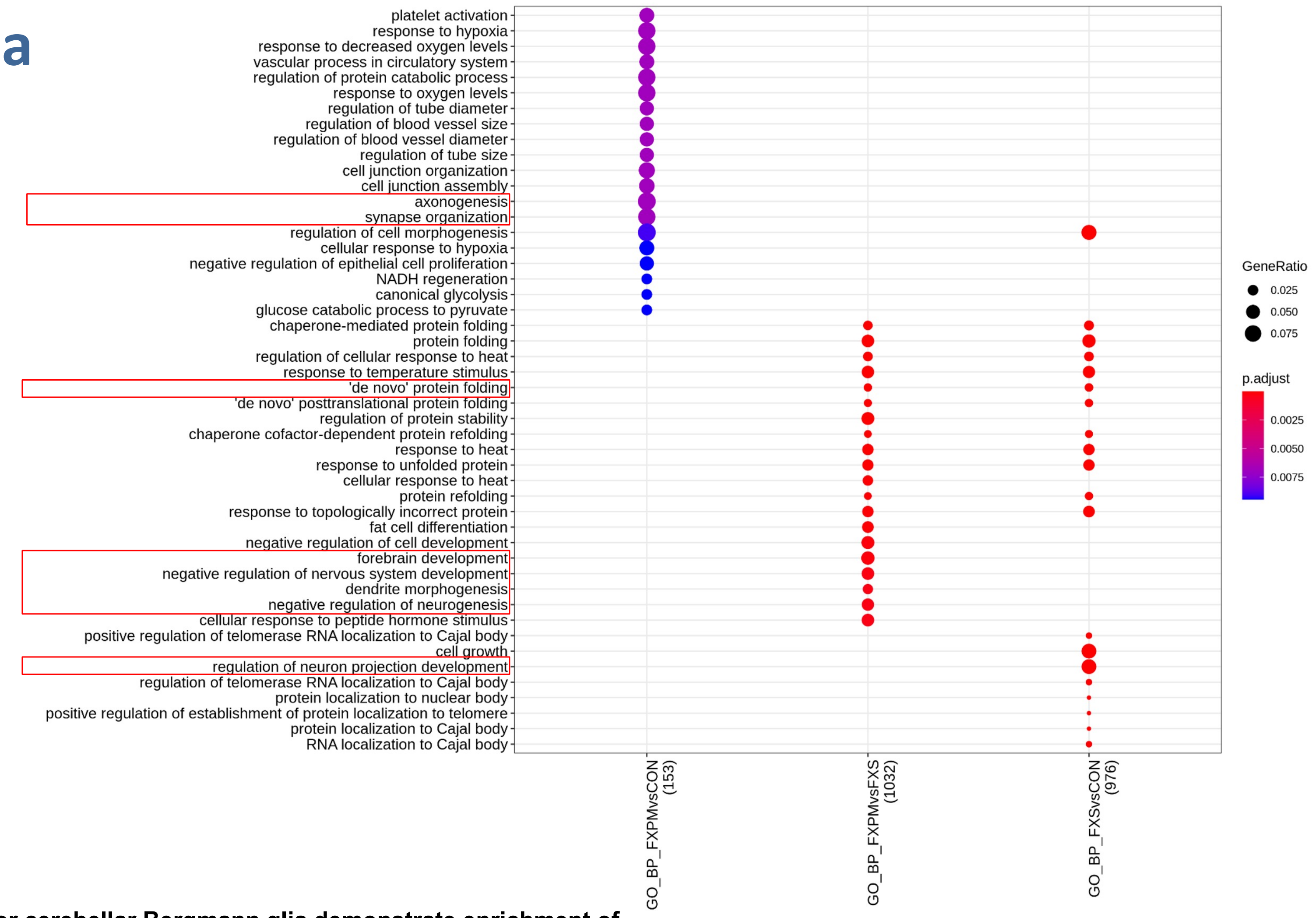

GO Biological Processes for cerebellar Bergmann glia demonstrate enrichment of hypoxia and vascular terms (PM comparisons), and protein folding (FXS comparisons).

Bergmann Glia

KEGG

Top 20

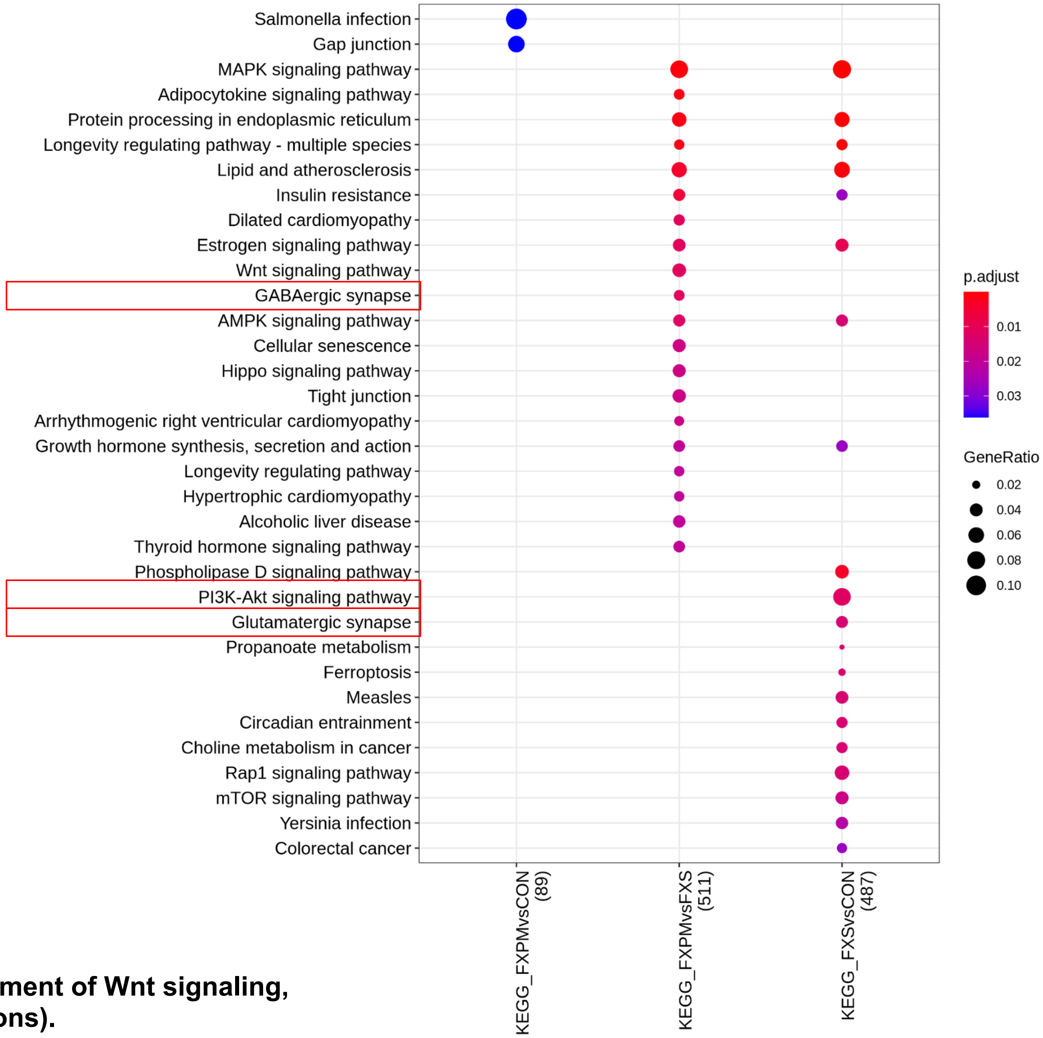

KEGG for cerebellar Bergmann glia demonstrate enrichment of Wnt signaling, PI3K-Akt signaling, and MAPK signaling (FXS comparisons).

Endothelial

GO_BP

Top 20

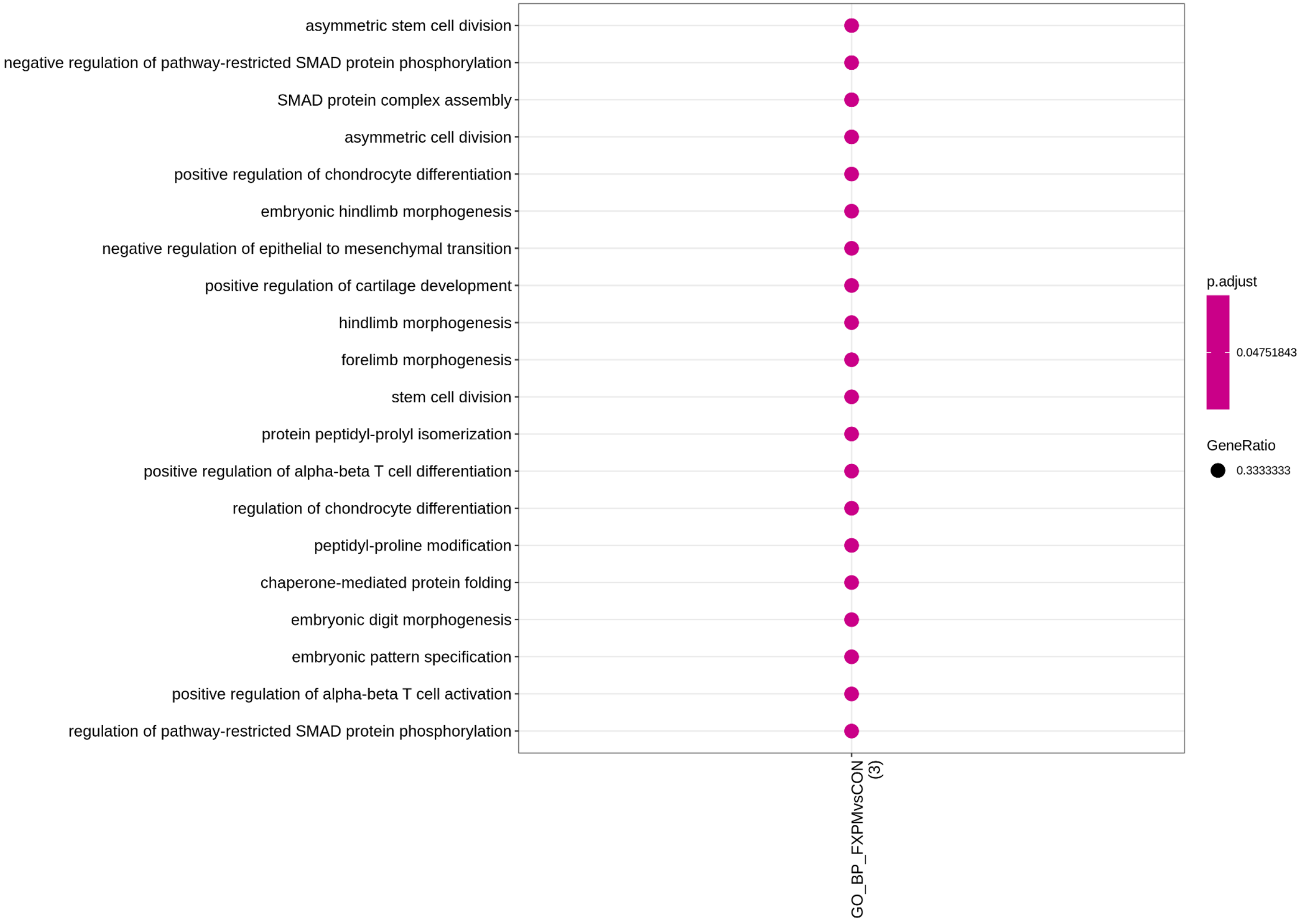

GO Biological Processes for endothelial cells demonstrate enrichment of development/morphogenesis and protein folding (PM comparisons).

Endothelial

KEGG

Top 20

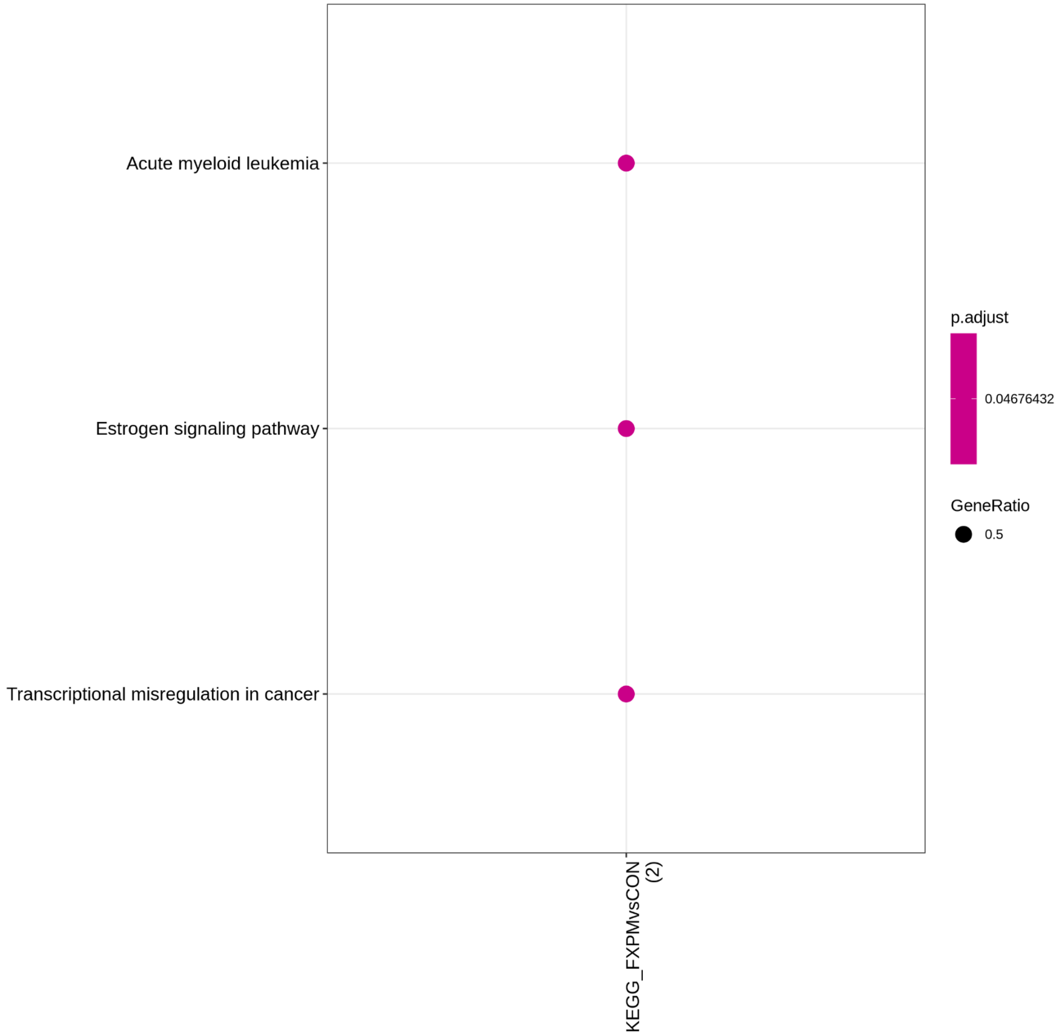

Granule

GO_BP

Top 20

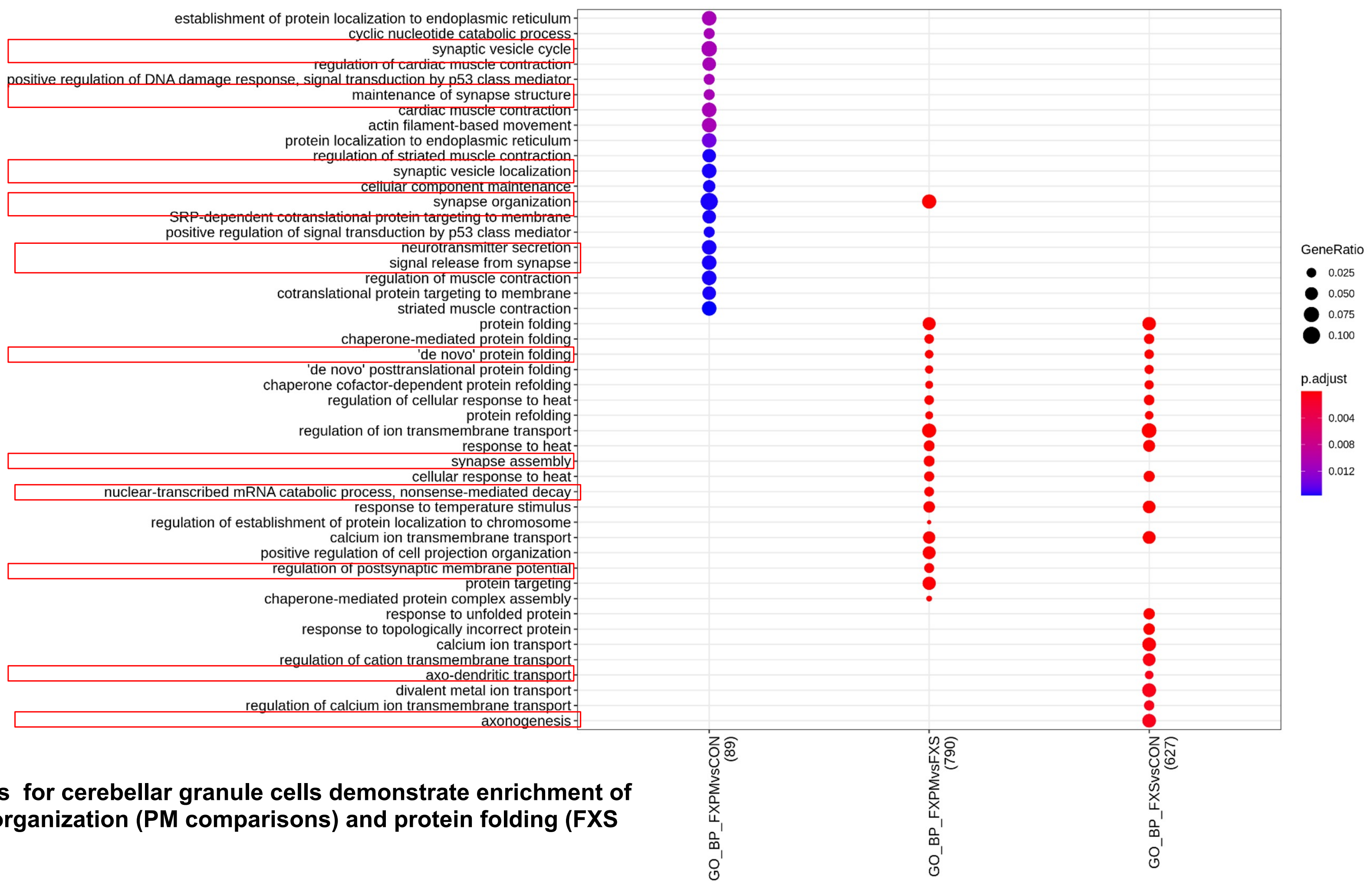

GO Biological Processes for cerebellar granule cells demonstrate enrichment of synaptic structure and organization (PM comparisons) and protein folding (FXS comparisons).

Granule

KEGG

Top 20

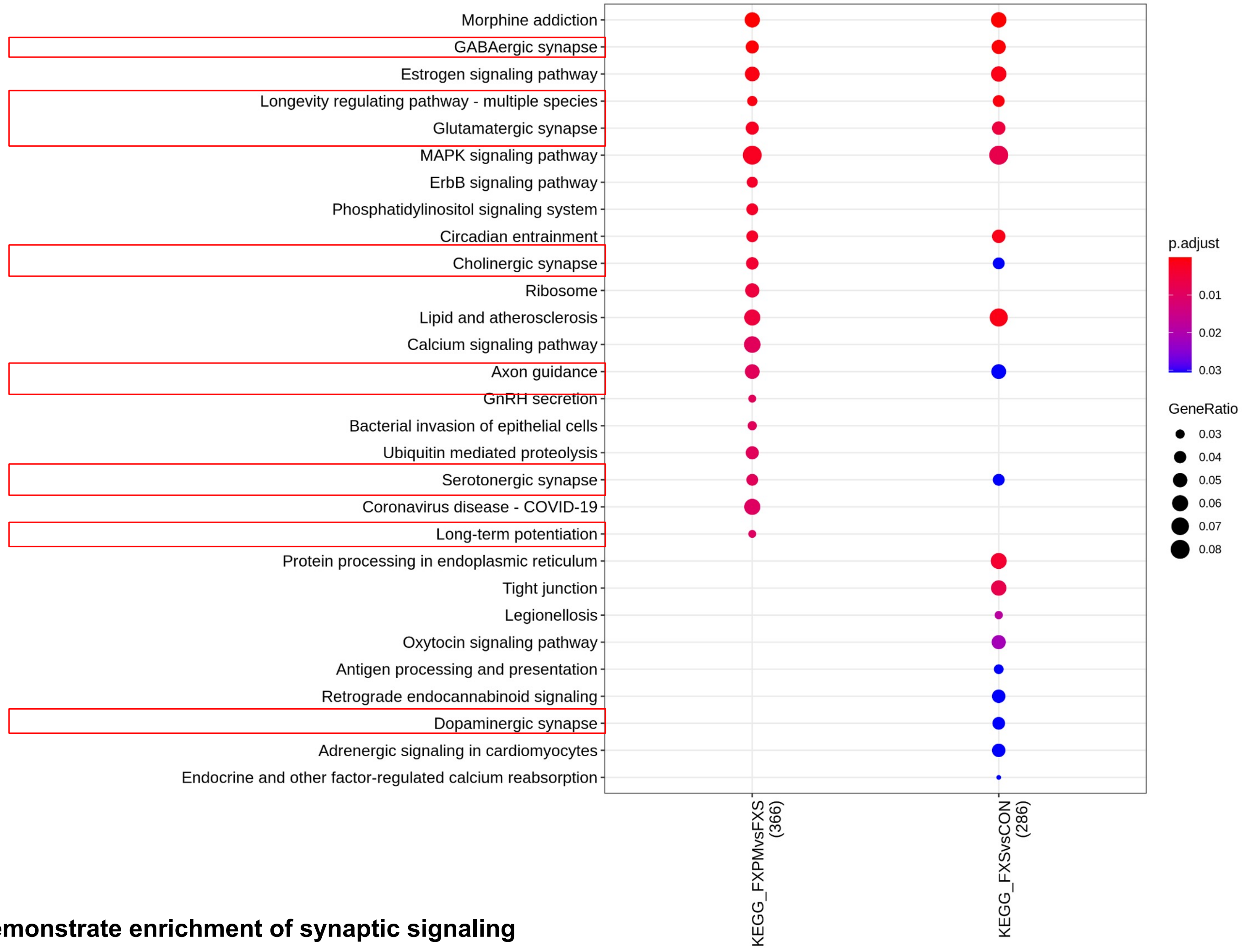

KEGG for cerebellar granule cells demonstrate enrichment of synaptic signaling (PM and FXS comparisons).

Interneuron

GO_BP

Top 20

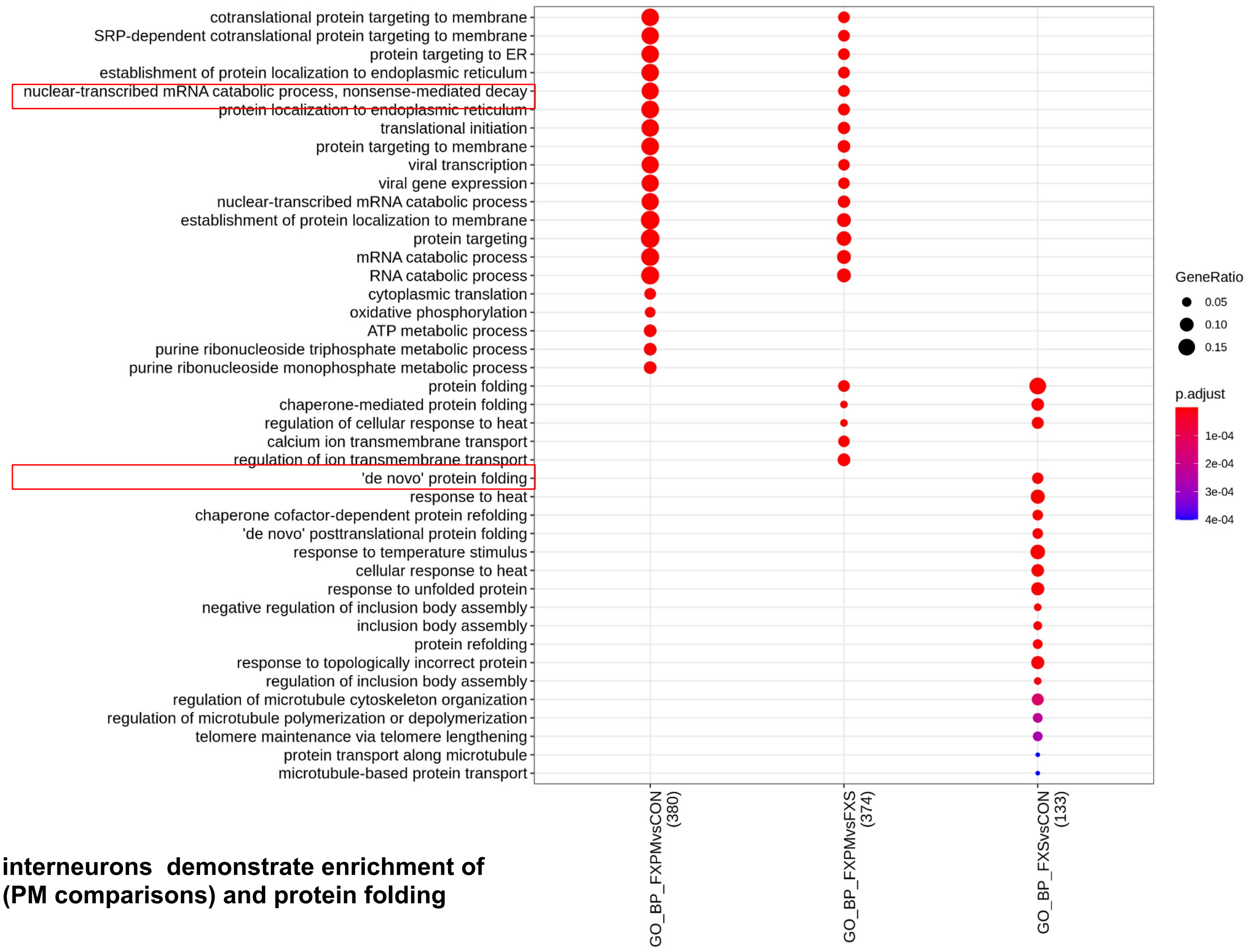

GO Biological Processes for cerebellar interneurons demonstrate enrichment of mRNA catabolism and protein targeting (PM comparisons) and protein folding (FXS comparisons).

Interneuron

KEGG

Top 20

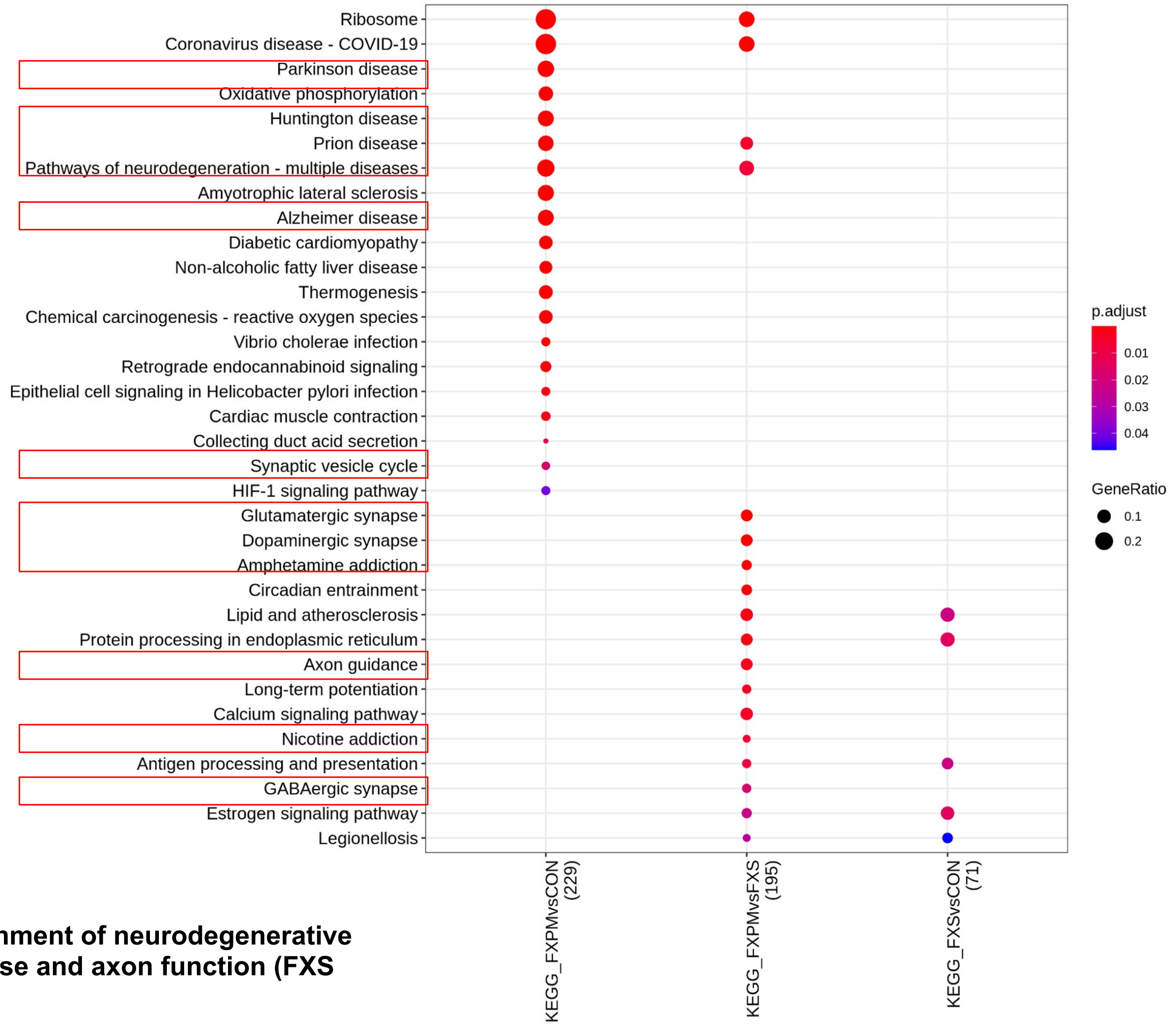

KEGG for cerebellar interneurons demonstrate enrichment of neurodegenerative terms and prion disease (PM comparisons) and synapse and axon function (FXS comparisons).

Interneuron II

GO_BP

Top 20

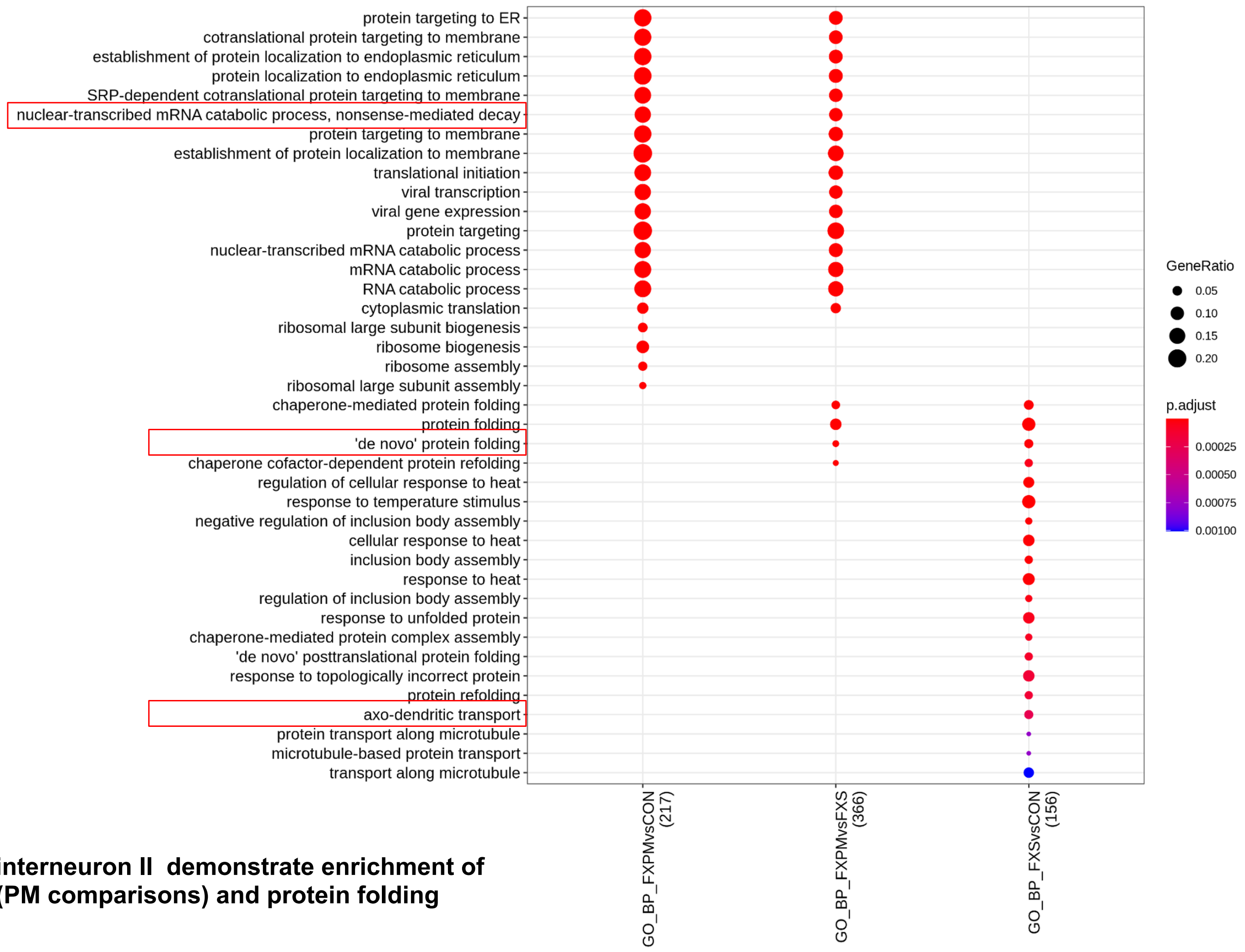

GO Biological Processes for cerebellar interneuron II demonstrate enrichment of mRNA catabolism and protein targeting (PM comparisons) and protein folding (FXS comparisons).

Interneuron II

KEGG

Top 20

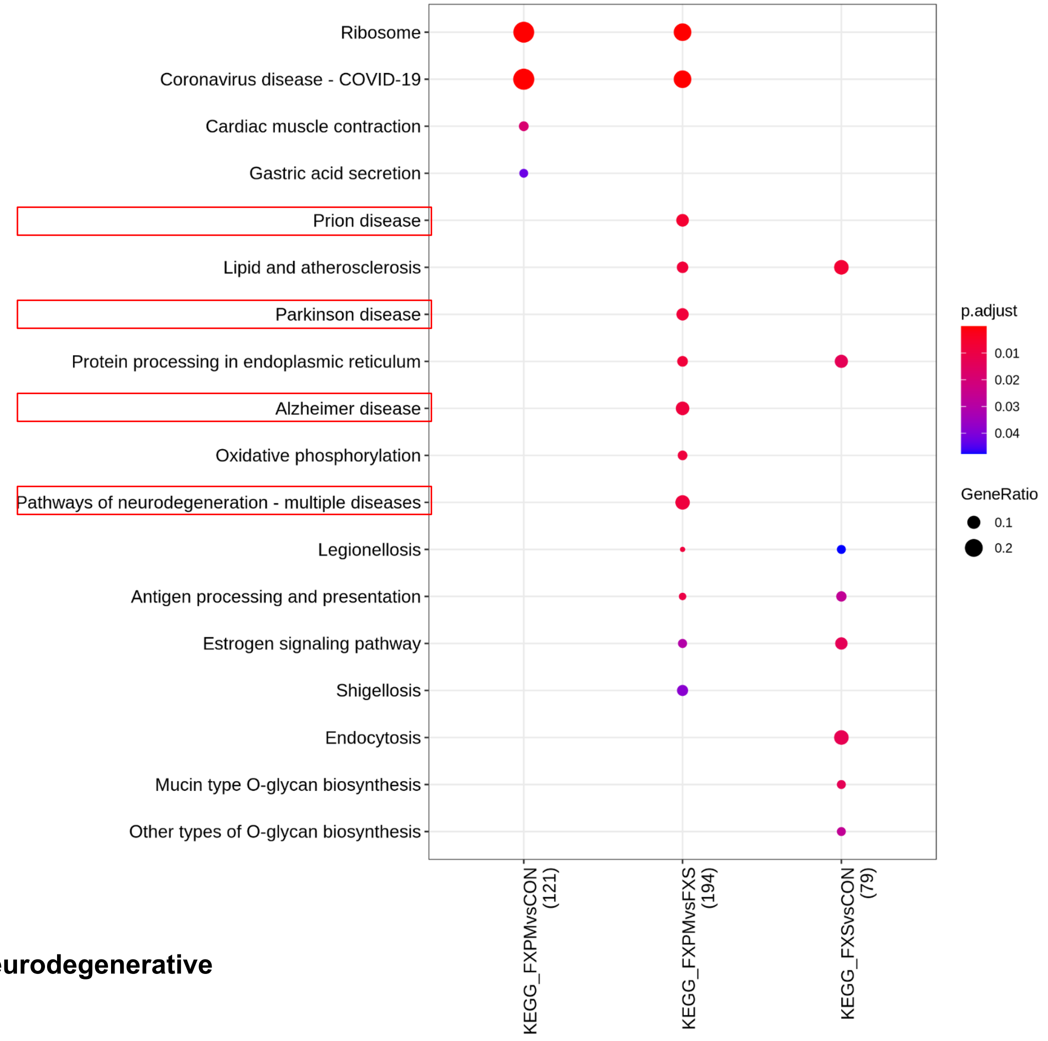

KEGG for cerebellar interneuron II demonstrate enrichment of neurodegenerative and prion terms (PM and FXS comparisons).

Microglia

GO_BP

Top 20

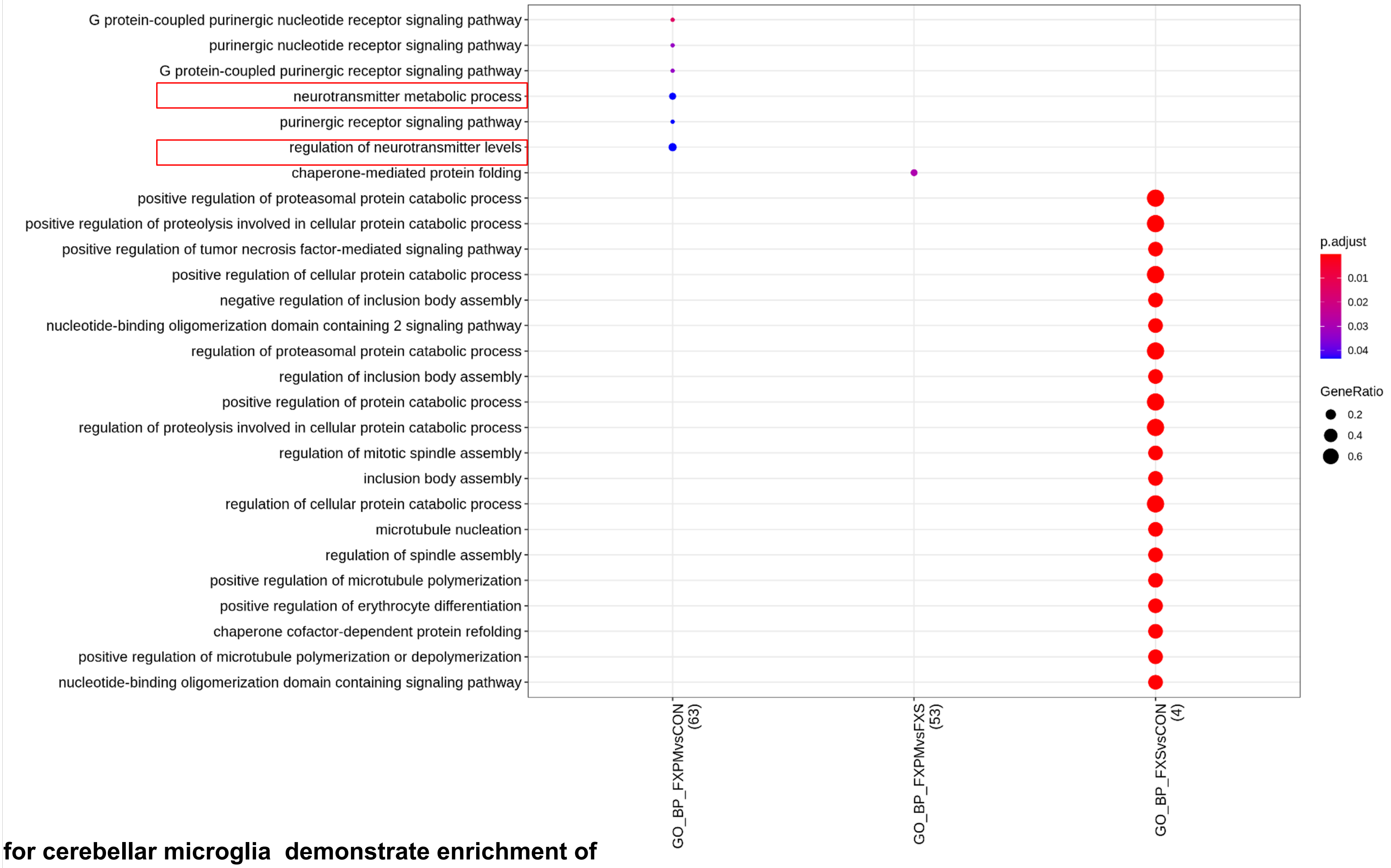

GO Biological Processes for cerebellar microglia demonstrate enrichment of neurotransmitter metabolism (PM comparisons) and protein catabolism (FXS comparisons).

Microglia

KEGG

Top 20

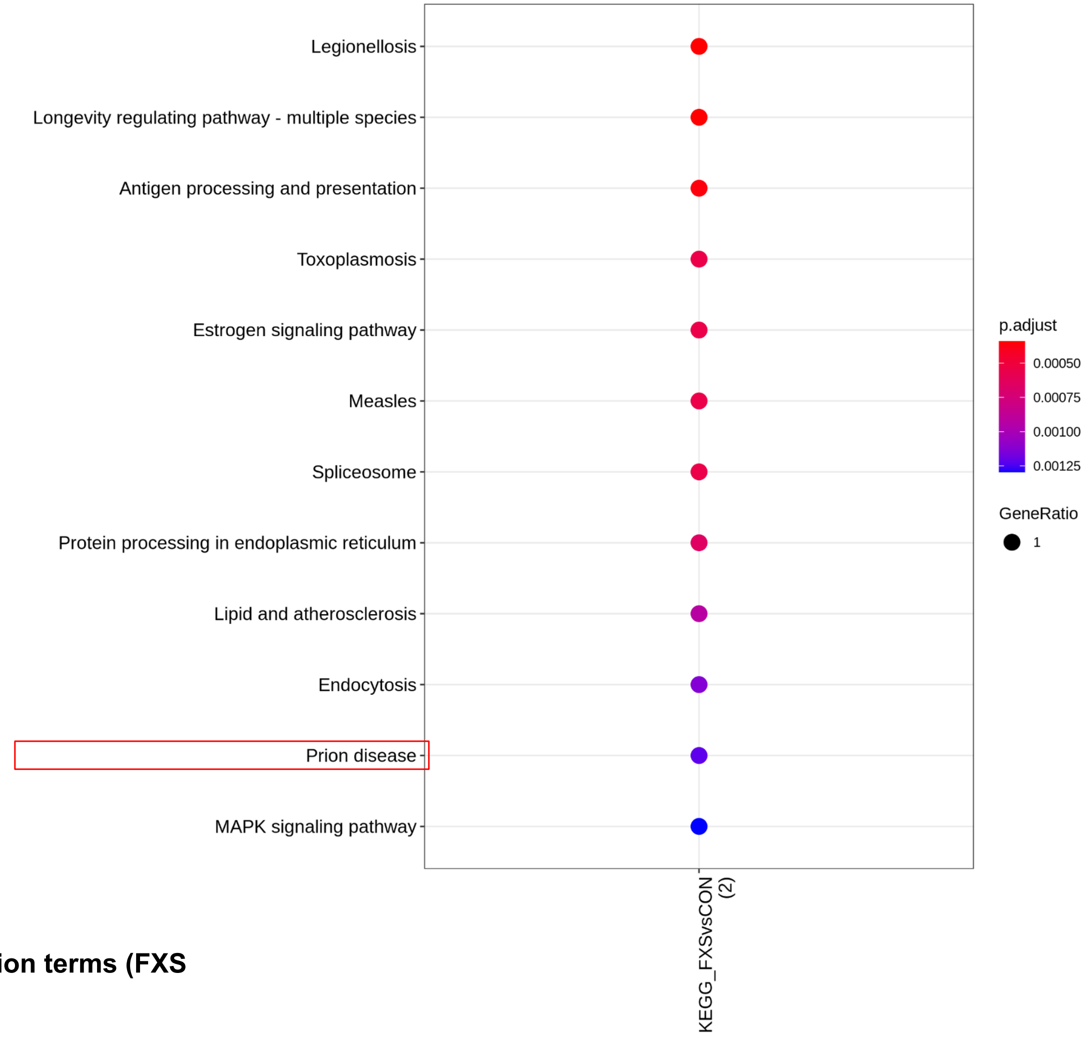

KEGG for cerebellar microglia demonstrate enrichment of prion terms (FXS comparisons).

Oligo

GO_BP

Top 20

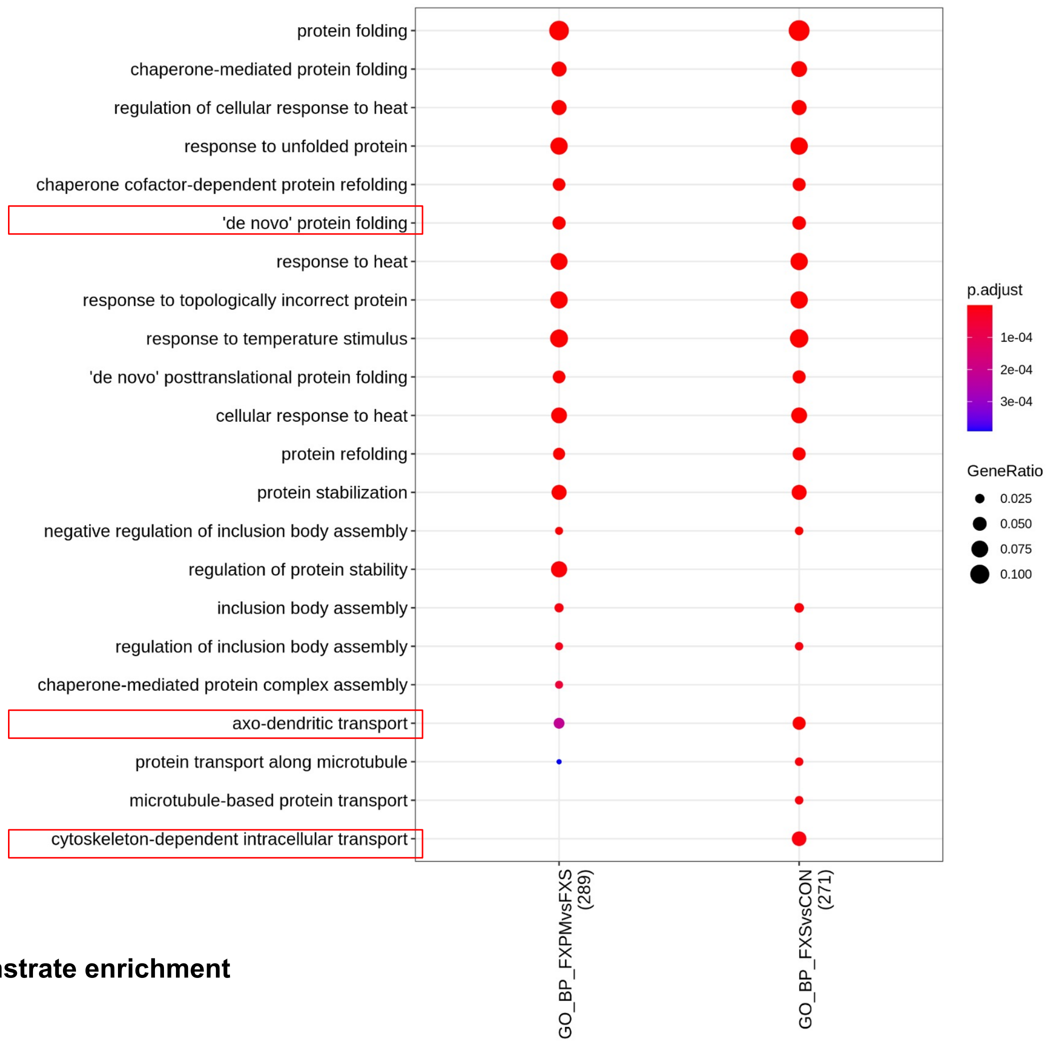

GO Biological Processes for cerebellar oligodendrocytes demonstrate enrichment of protein folding terms (PM and FXS comparisons).

Oligo

KEGG

Top 20

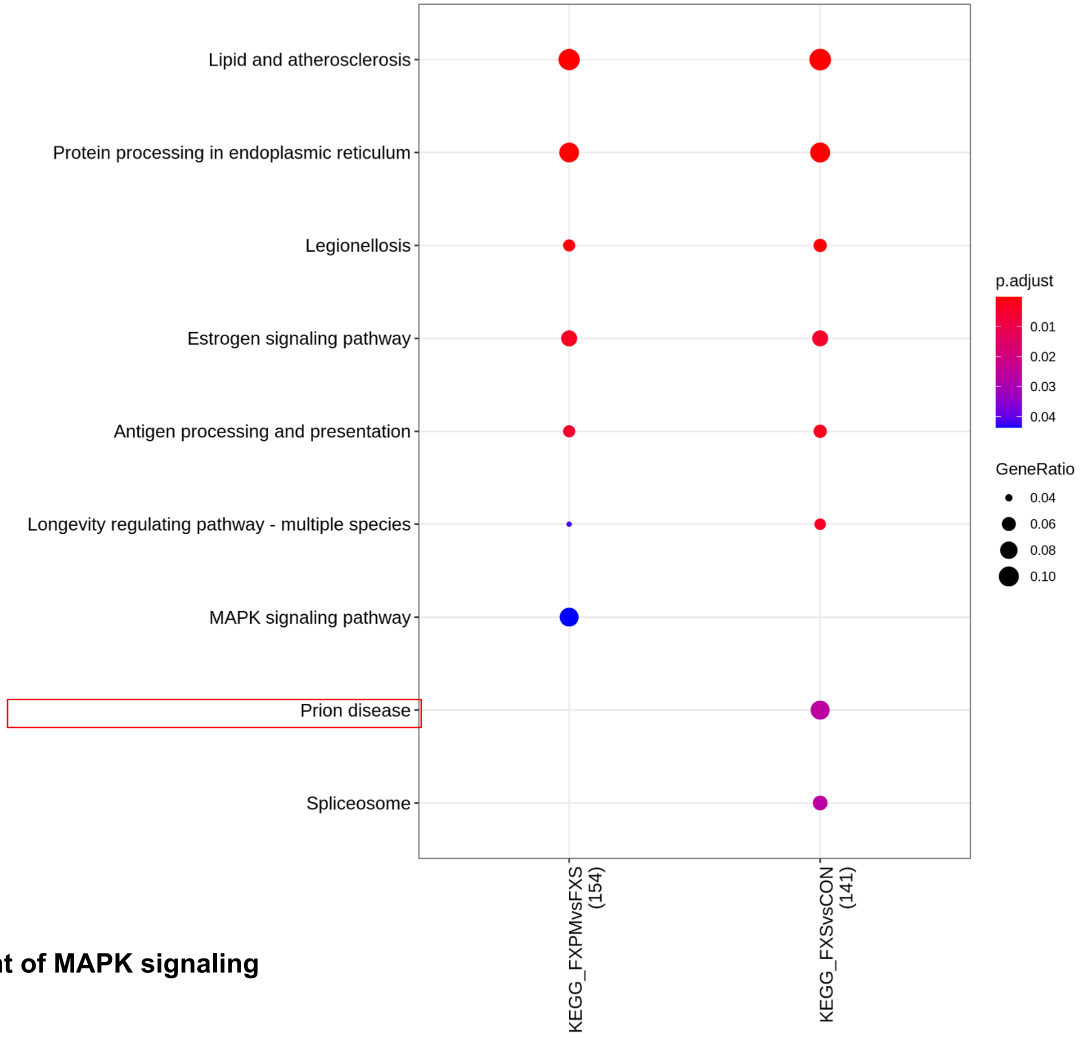

KEGG for cerebellar oligodendrocytes demonstrate enrichment of MAPK signaling and prion terms (PM and FXS comparisons).

OPC

GO_BP

Top 20

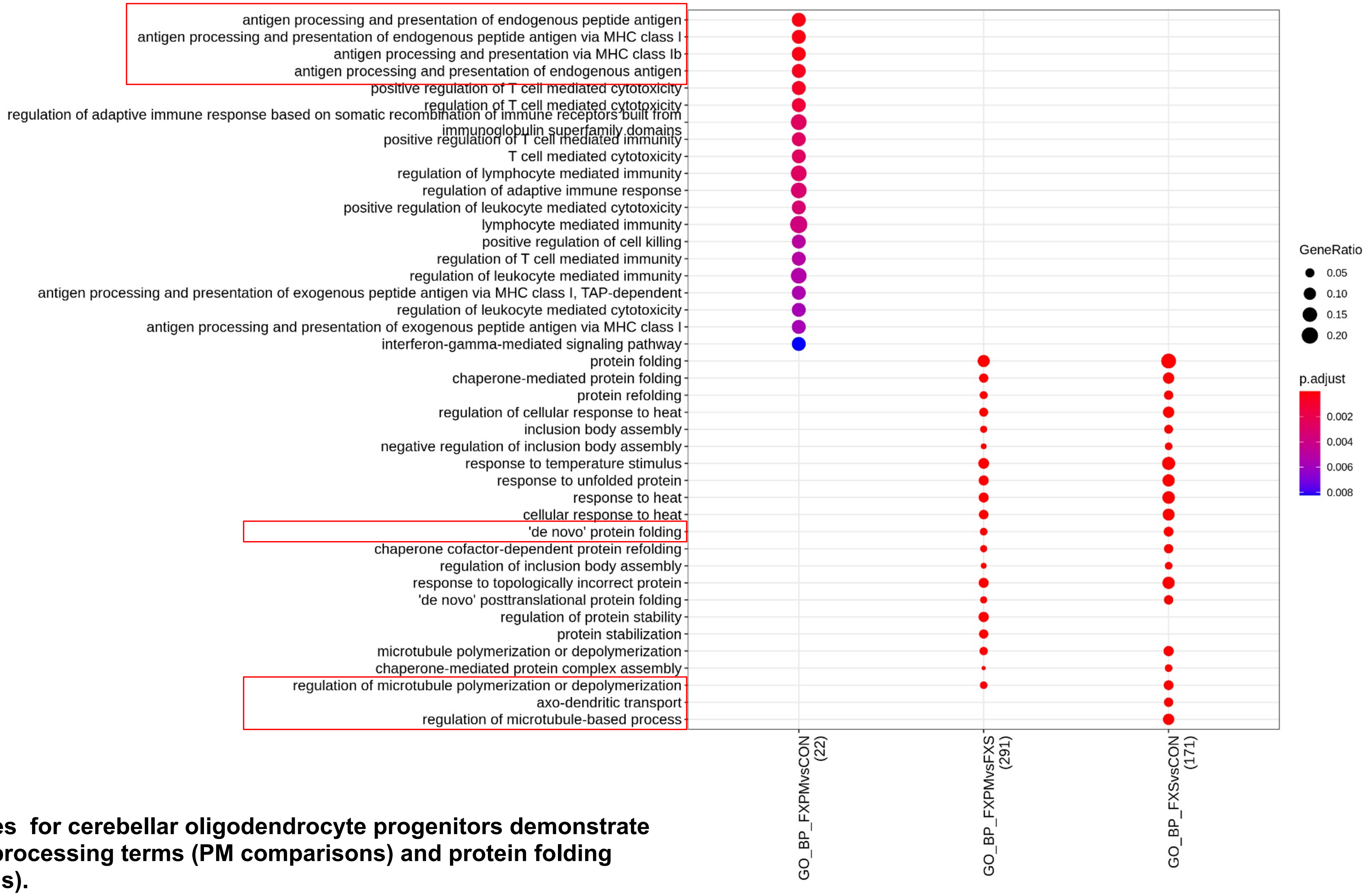

GO Biological Processes for cerebellar oligodendrocyte progenitors demonstrate enrichment of antigen processing terms (PM comparisons) and protein folding terms (FXS comparisons).

OPC

KEGG

Top 20

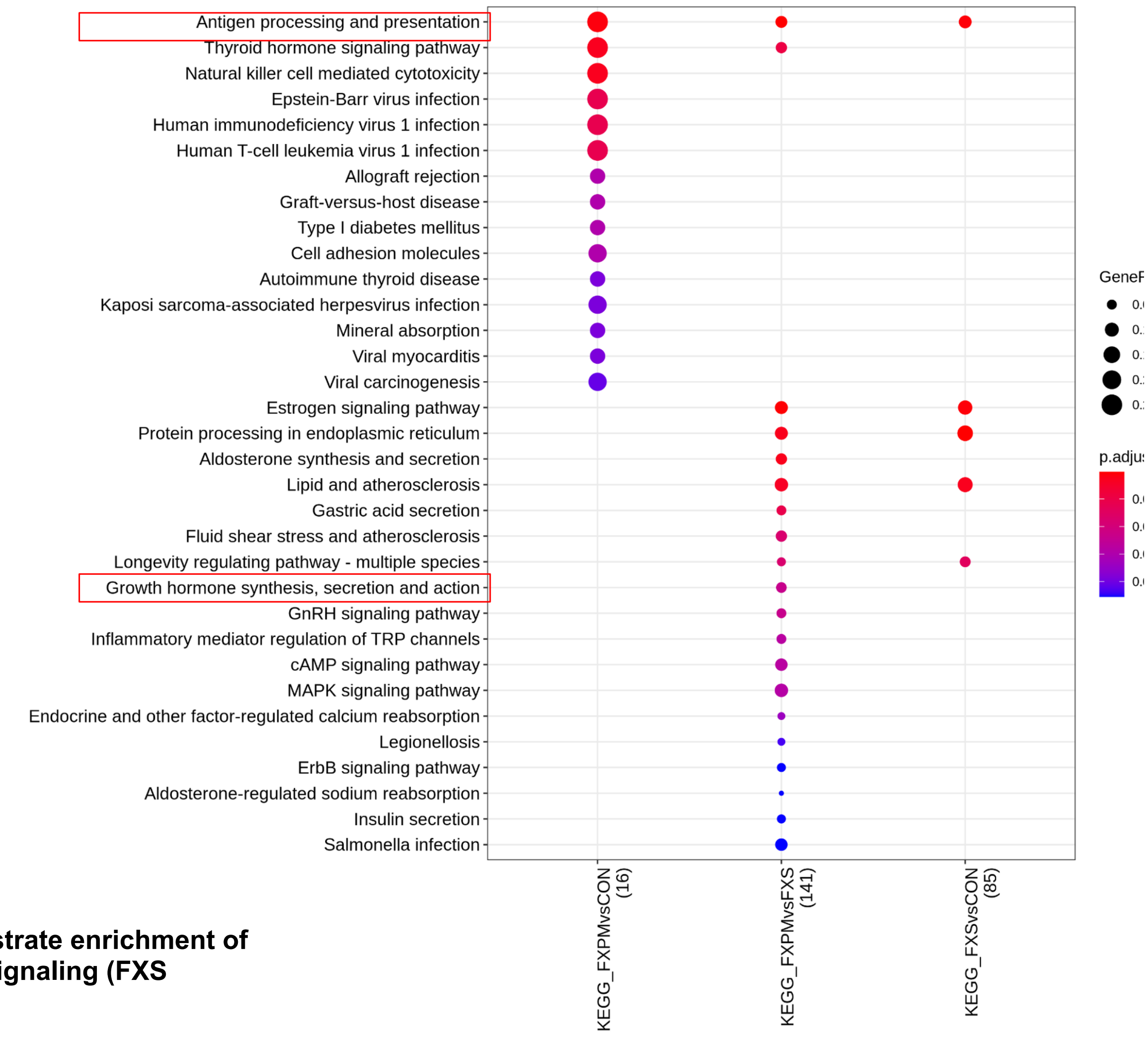

KEGG for cerebellar oligodendrocyte progenitors demonstrate enrichment of antigen processing terms (PM comparisons) and MAPK signaling (FXS comparisons).

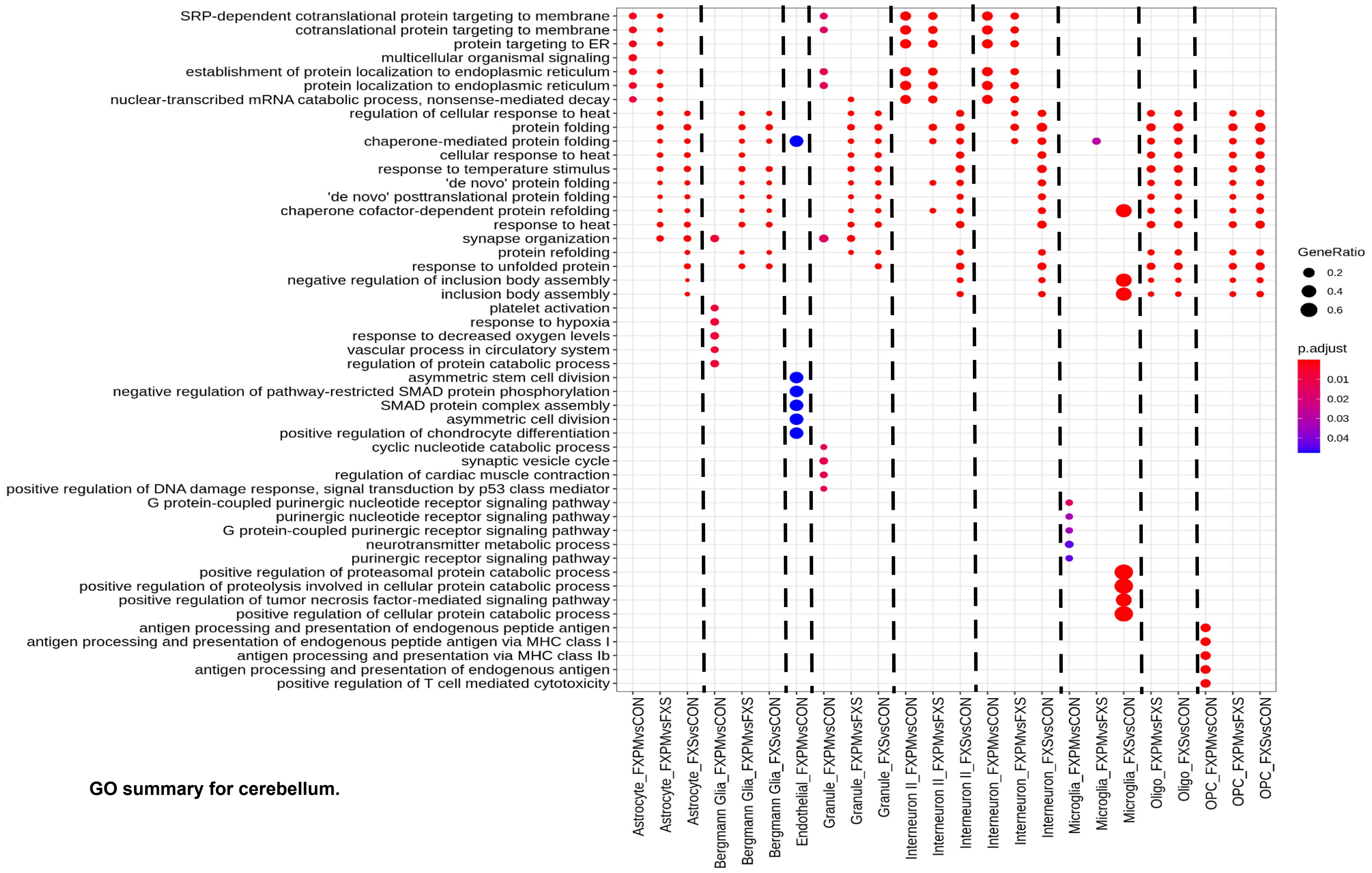

GO summary for cerebellum.

Supplemental Information File 3

Cortical top 20 terms for enriched biological processes (BP) and Kyoto Encyclopedia of Genes and Genomes (KEGG) terms for each cellular population with each condition comparison, potential terms of particular interest highlighted with red box.

Astro I

GO_BP

Top 20

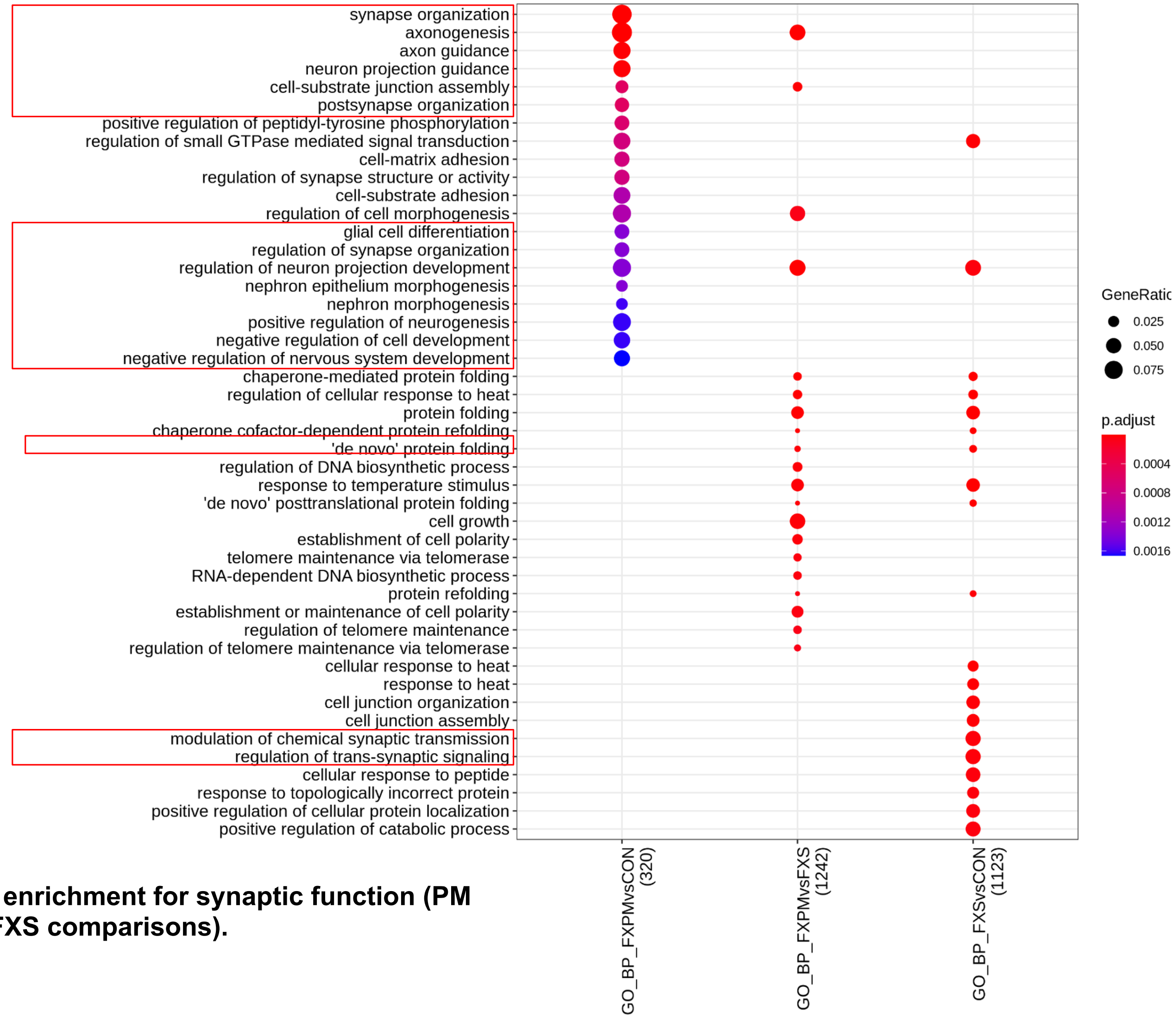

GO Biological Process for astrocyte I shows enrichment for synaptic function (PM and FXS comparisons) and protein folding (FXS comparisons).

Astro I

KEGG

Top 20

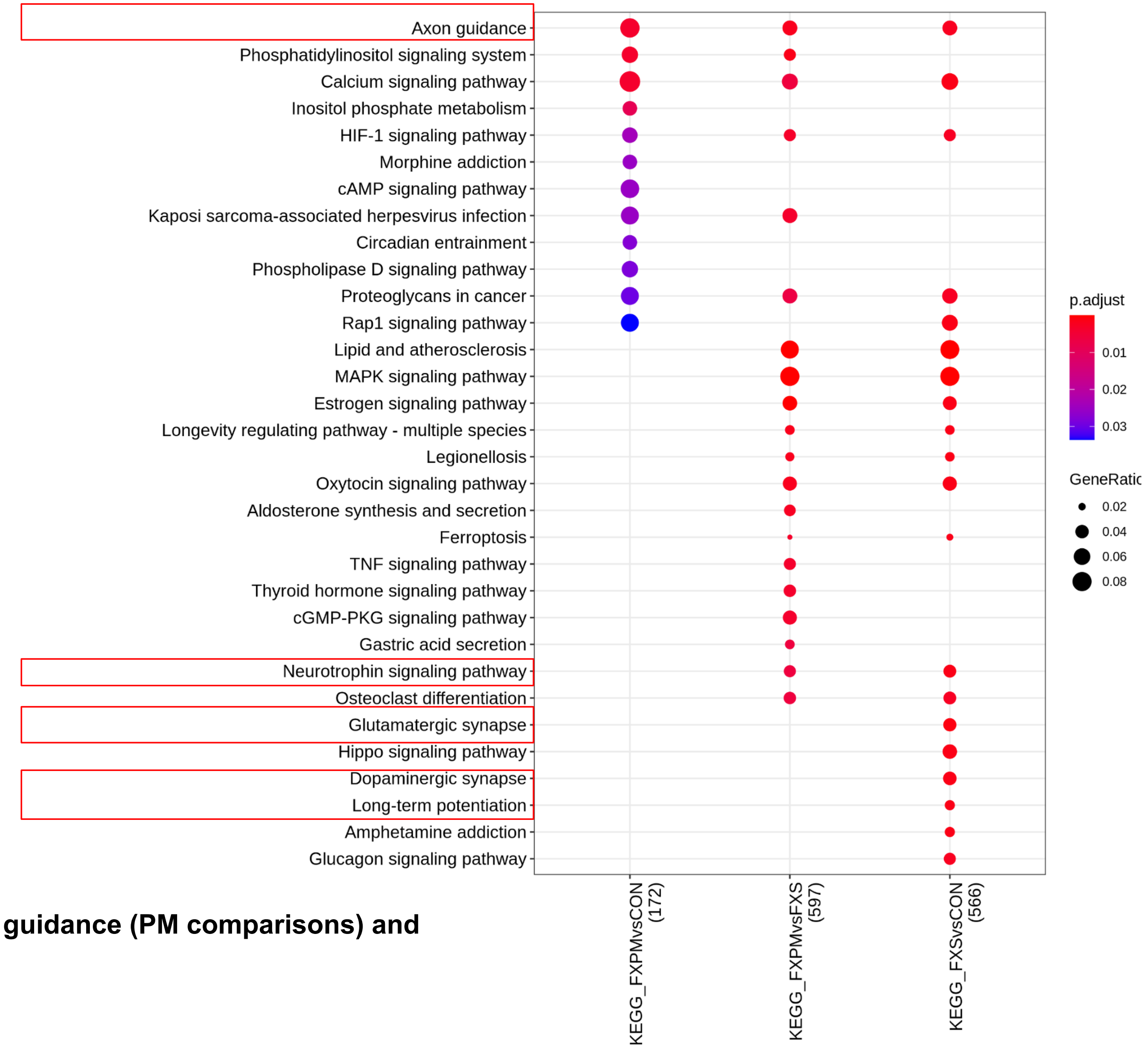

KEGG for astrocyte I shows enrichment for axon guidance (PM comparisons) and synaptic neurotransmission (FXS comparisons).

Astro II

GO_BP

Top 20

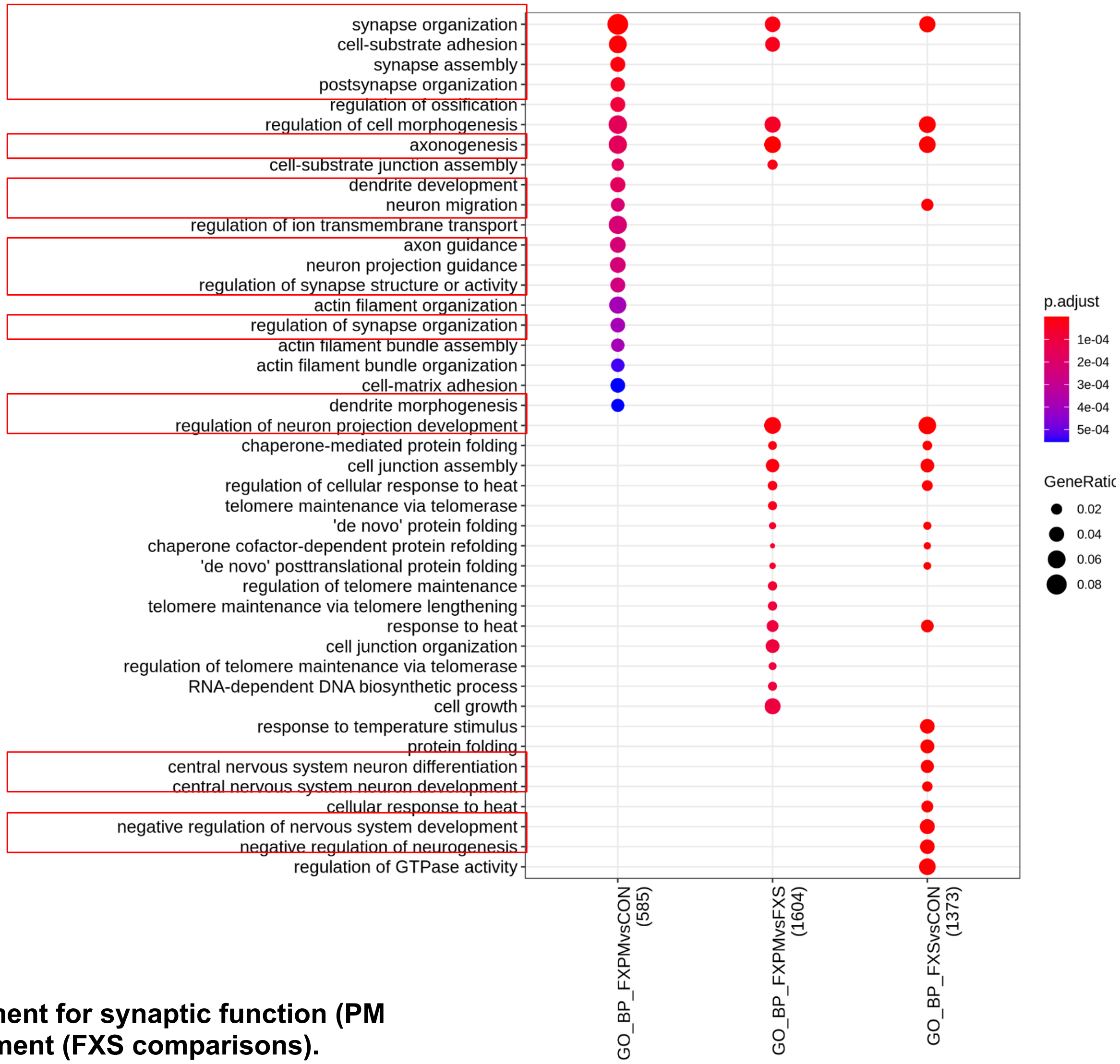

GO Biological Process for astrocyte II shows enrichment for synaptic function (PM and FXS comparisons) and nervous system development (FXS comparisons).

Astro II

KEGG

Top 20

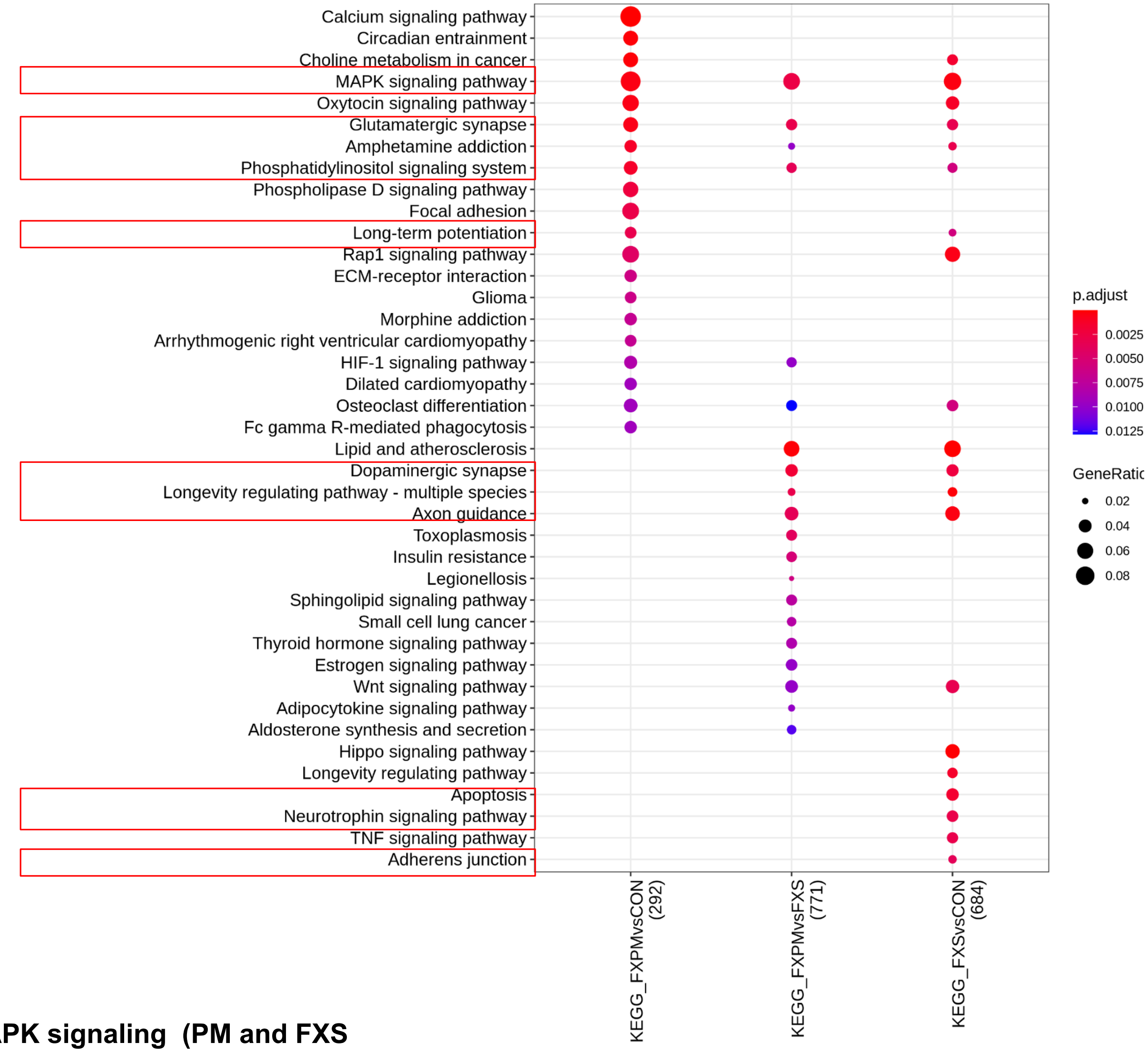

KEGG for astrocyte II shows enrichment for MAPK signaling (PM and FXS comparisons) and apoptosis (FXS comparisons).

Endo

GO_BP

Top 20

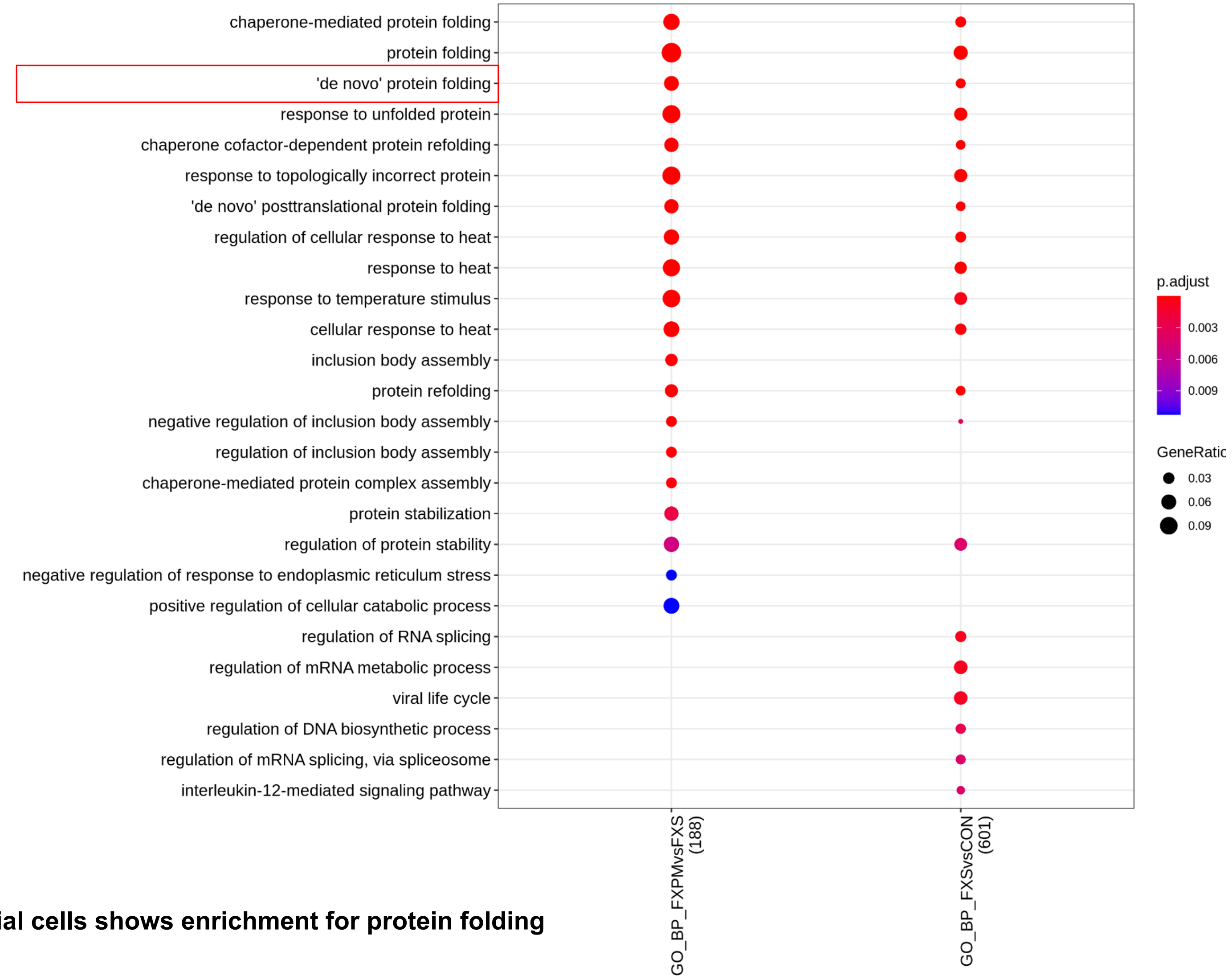

GO Biological Process for endothelial cells shows enrichment for protein folding (PM and FXS comparisons).

Endo

KEGG

Top 20

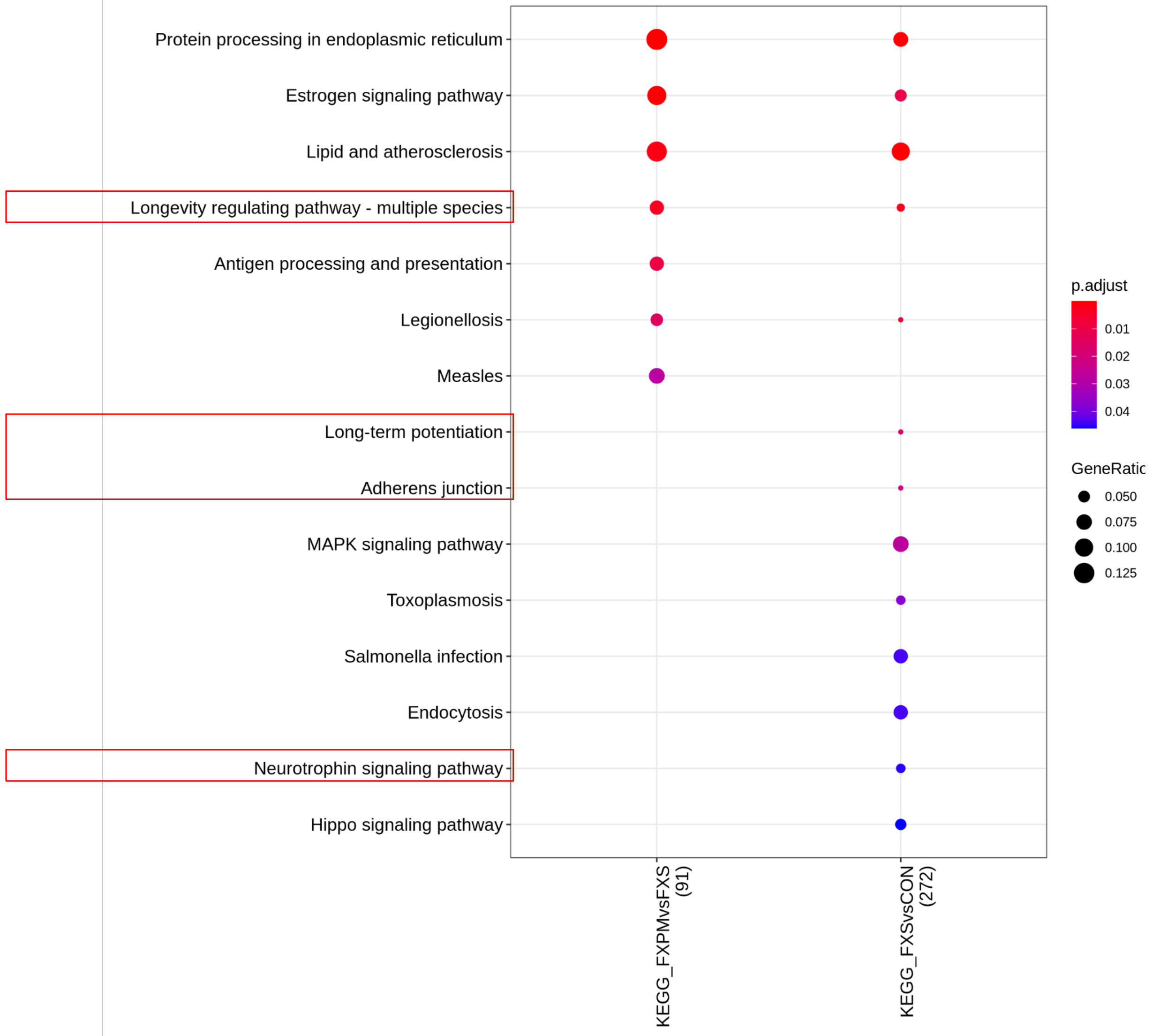

KEGG for endothelial cells shows enrichment for longevity (PM and FXS comparisons).

Inh-PVALB I

GO_BP

Top 20

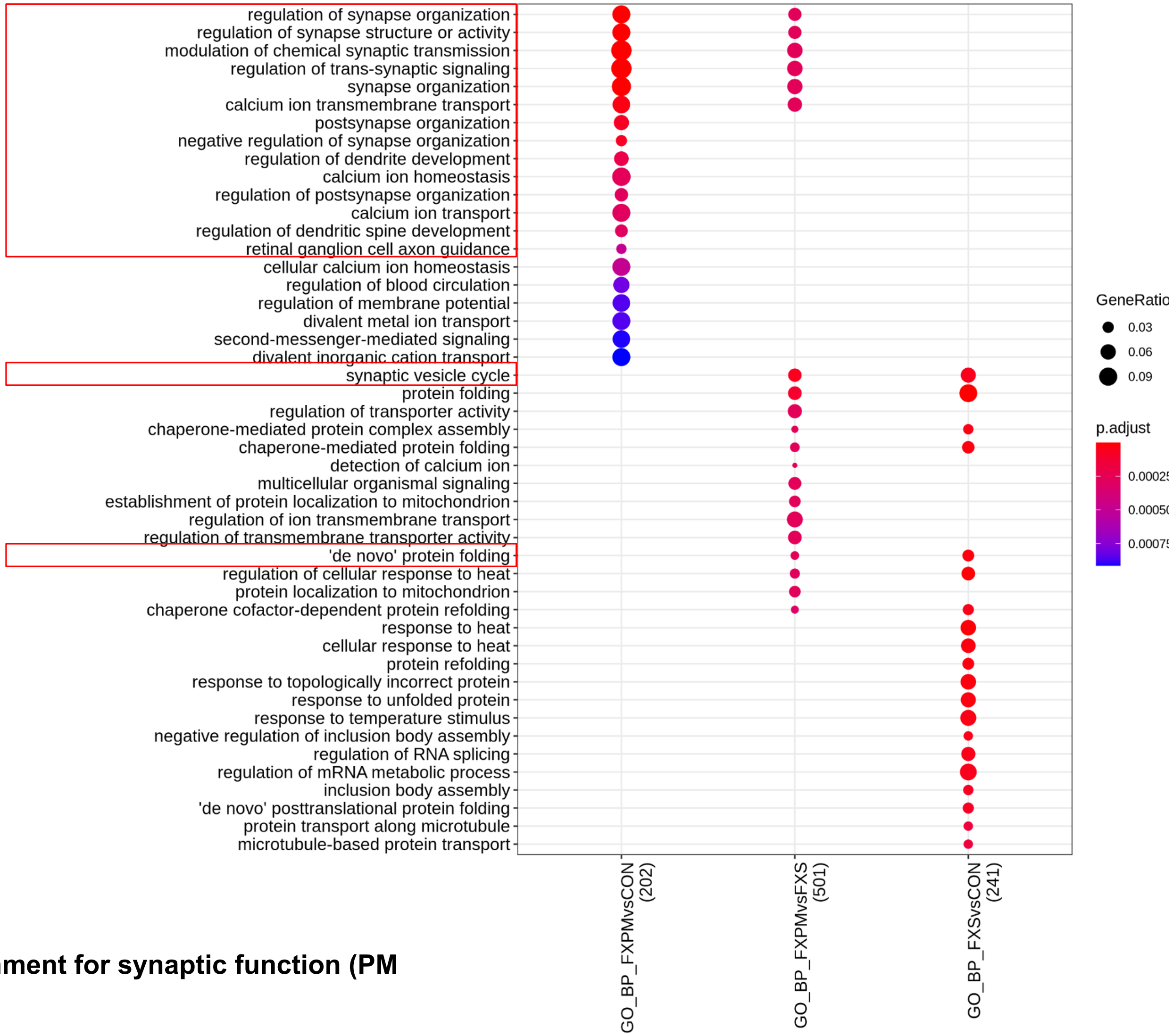

GO Biological Process for PVALB I shows enrichment for synaptic function (PM comparisons).

Inh-PVALB I

KEGG

Top 20

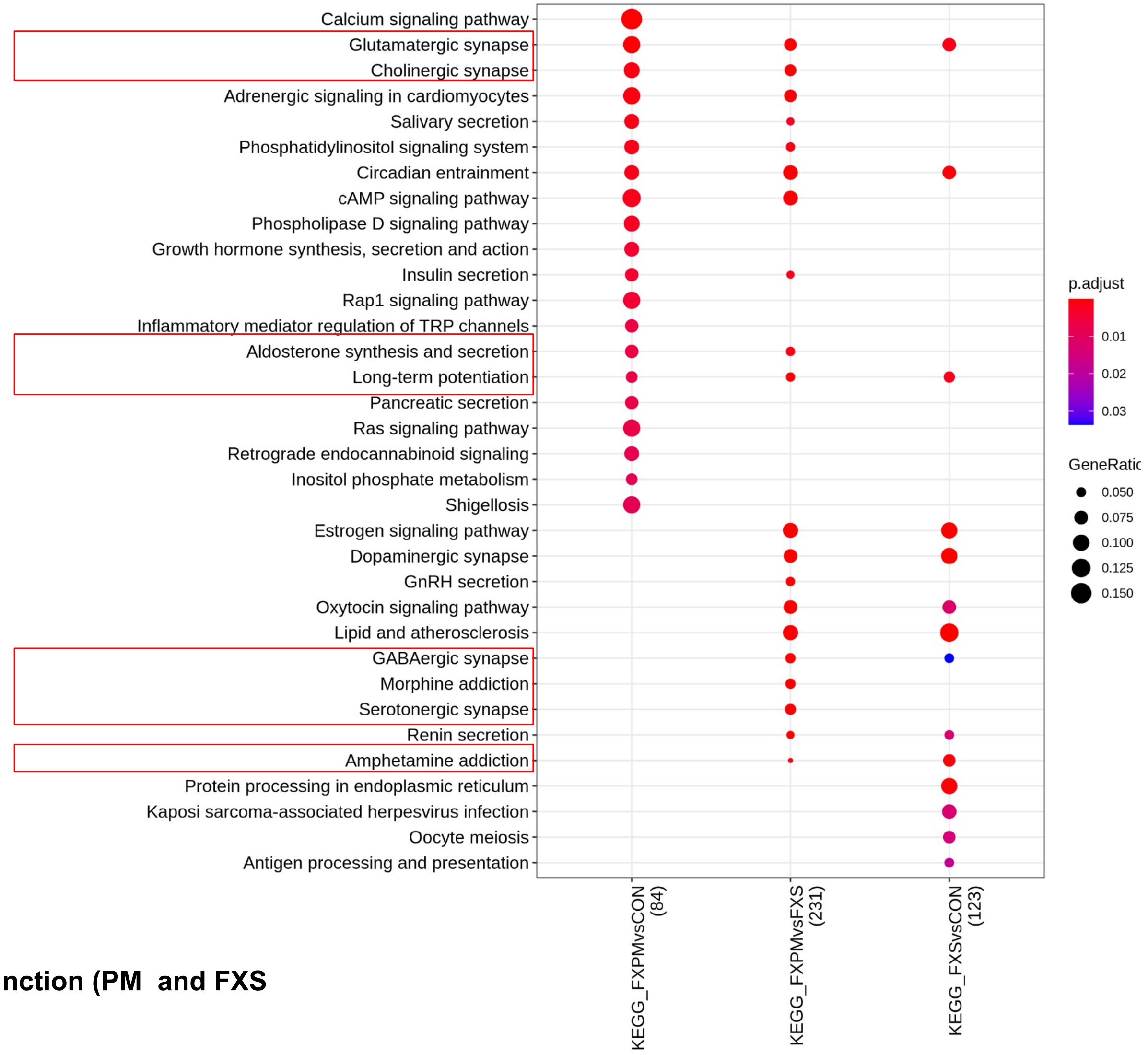

KEGG for PVALB I shows enrichment for synaptic function (PM and FXS comparisons).

Inh-PVALB II

GO_BP

Top 20

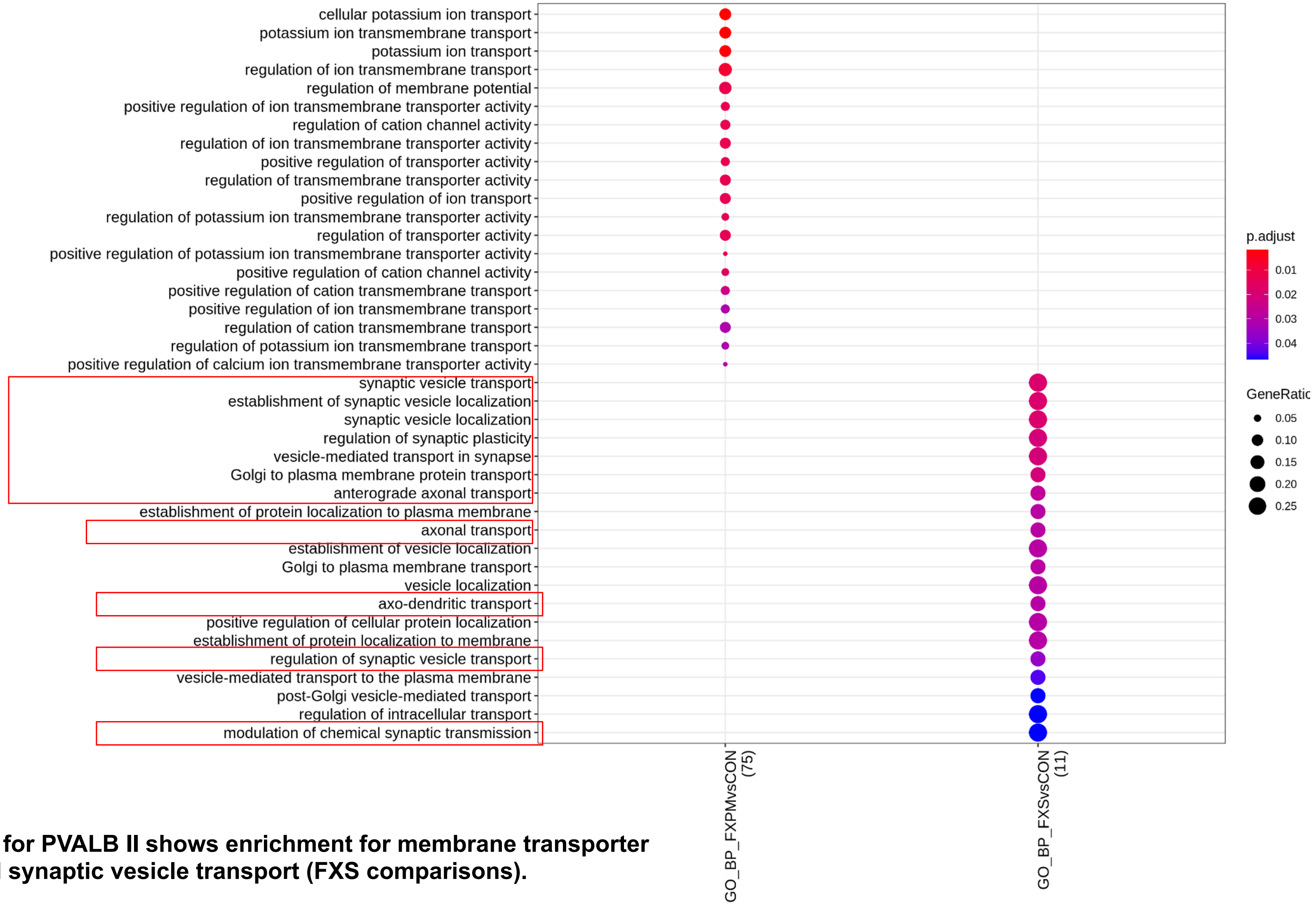

GO Biological Process for PVALB II shows enrichment for membrane transporter (PM comparisons) and synaptic vesicle transport (FXS comparisons).

Inh-PVALB II

KEGG

Top 20

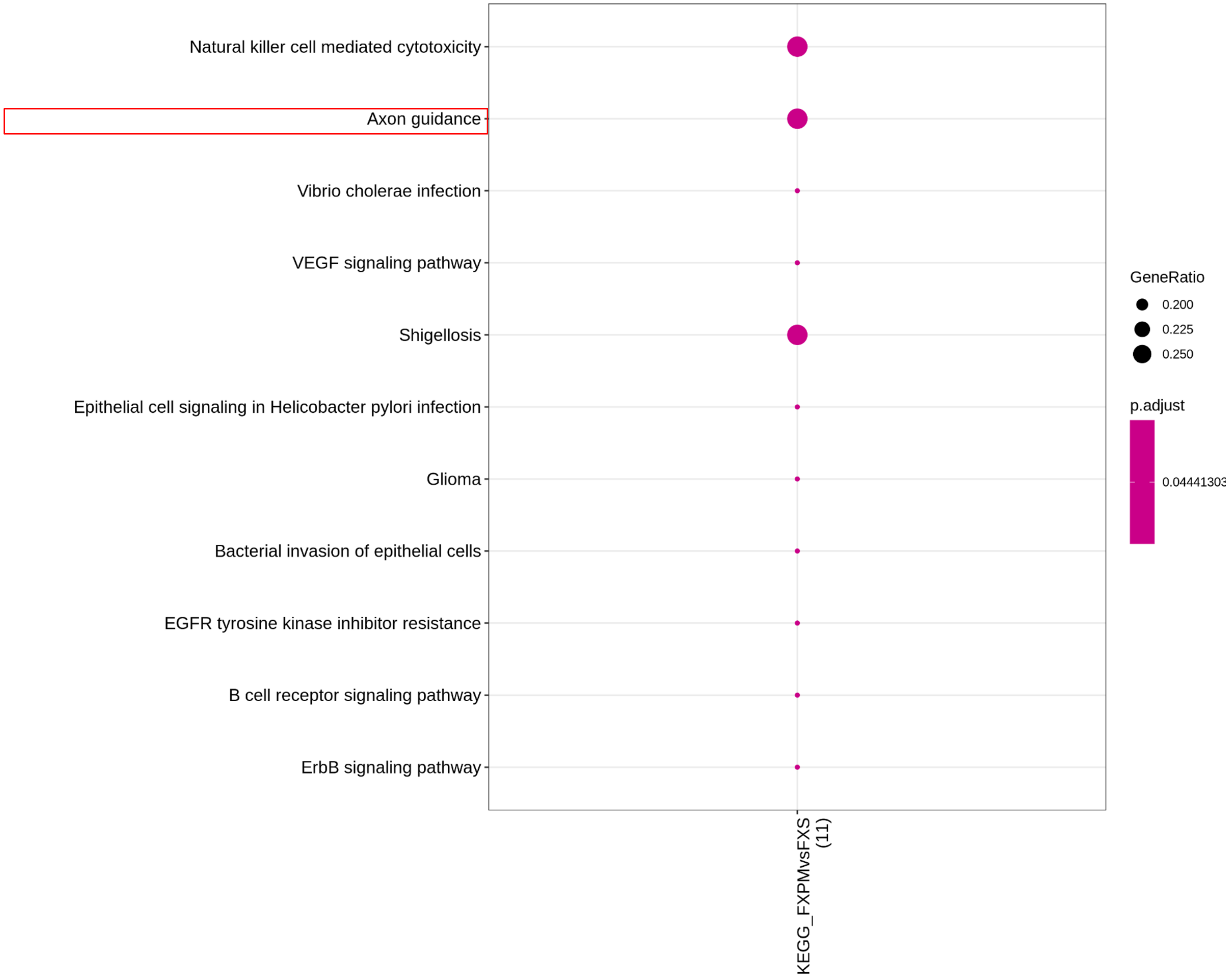

Inh-SST

GO_BP

Top 20

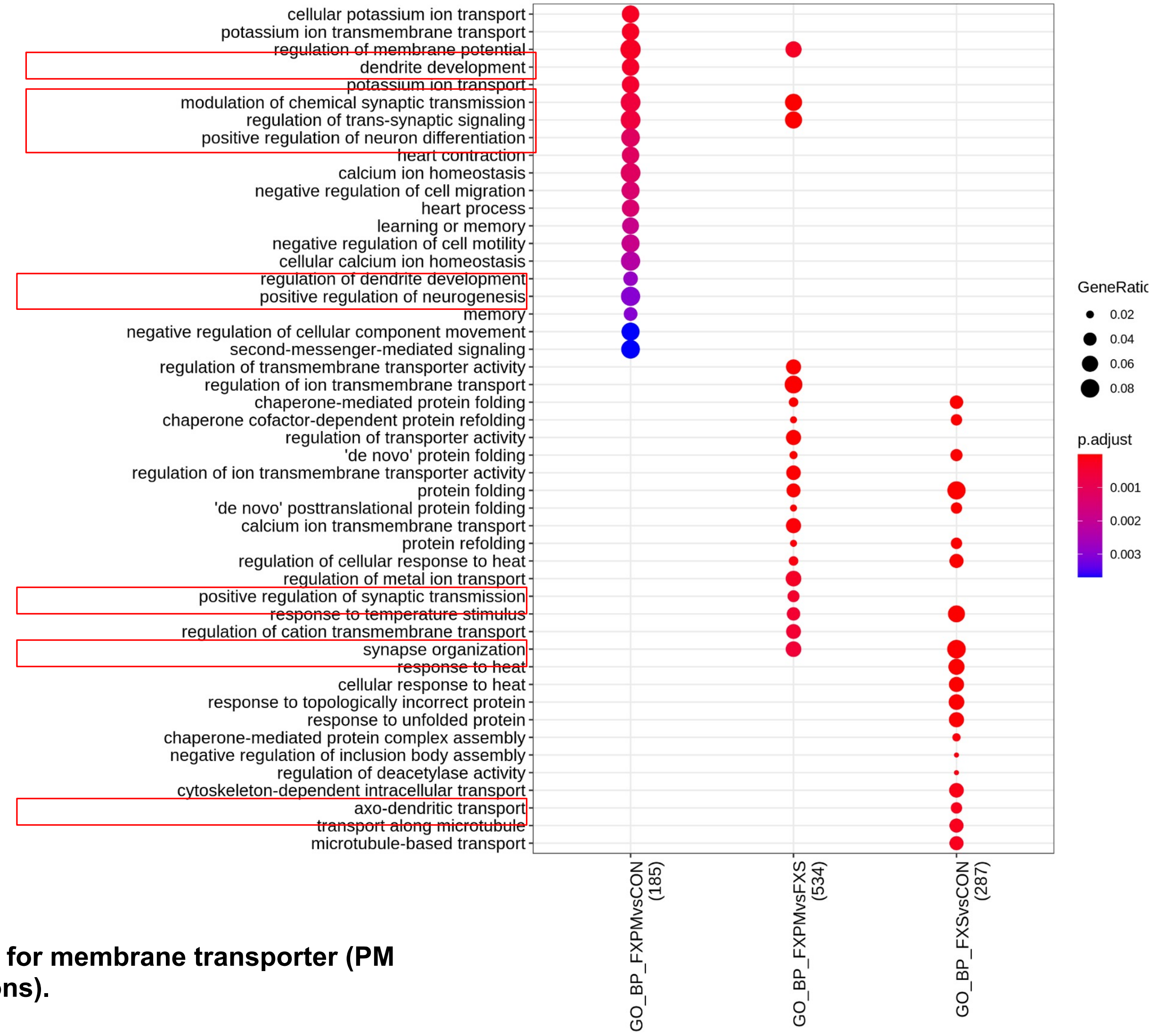

GO Biological Process for SST shows enrichment for membrane transporter (PM comparisons) and protein folding (FXS comparisons).

Inh-SST

KEGG

Top 20

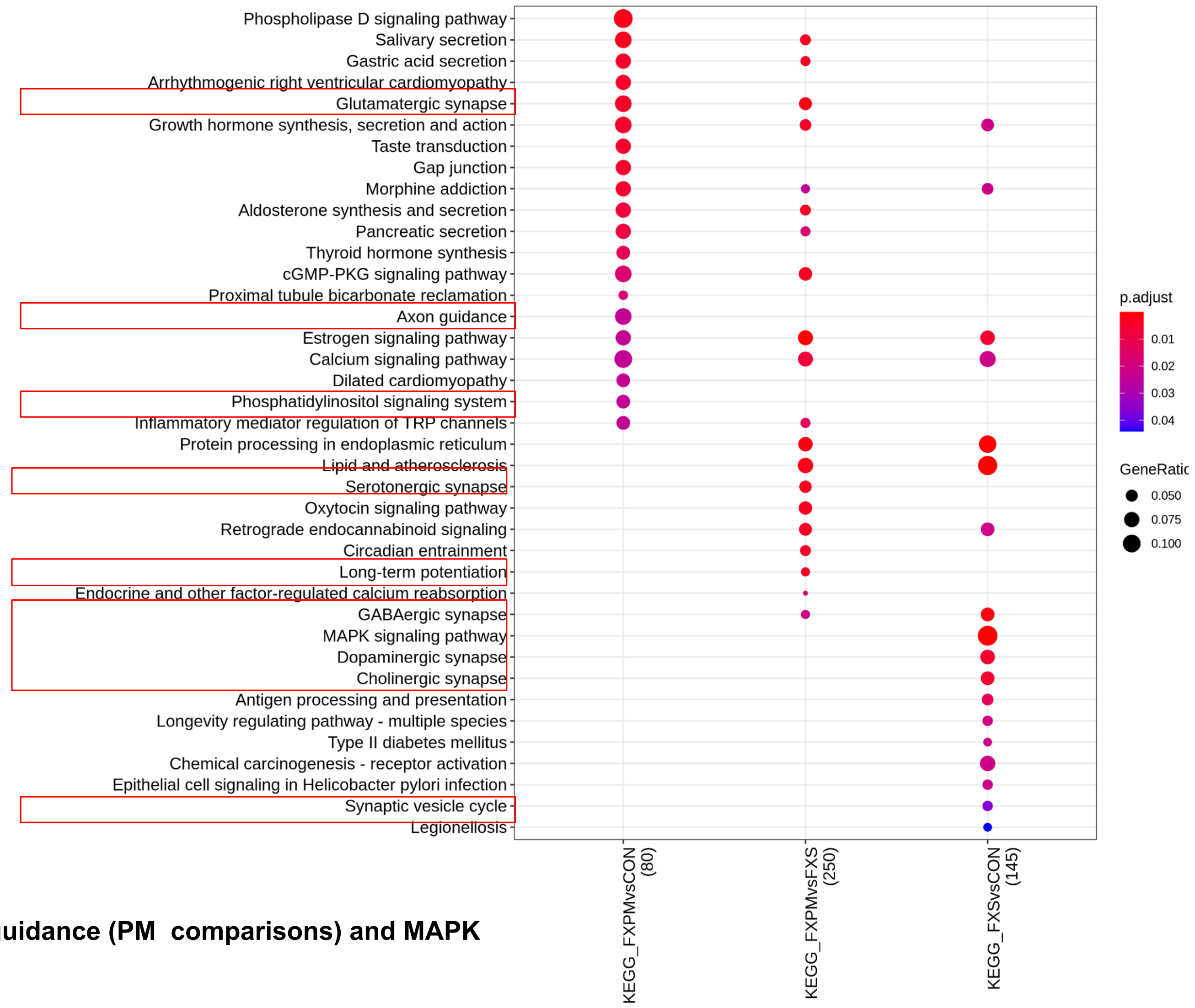

KEGG for SST shows enrichment for axon guidance (PM comparisons) and MAPK signaling (FXS comparisons).

Inh-SV2C I

GO_BP

Top 20

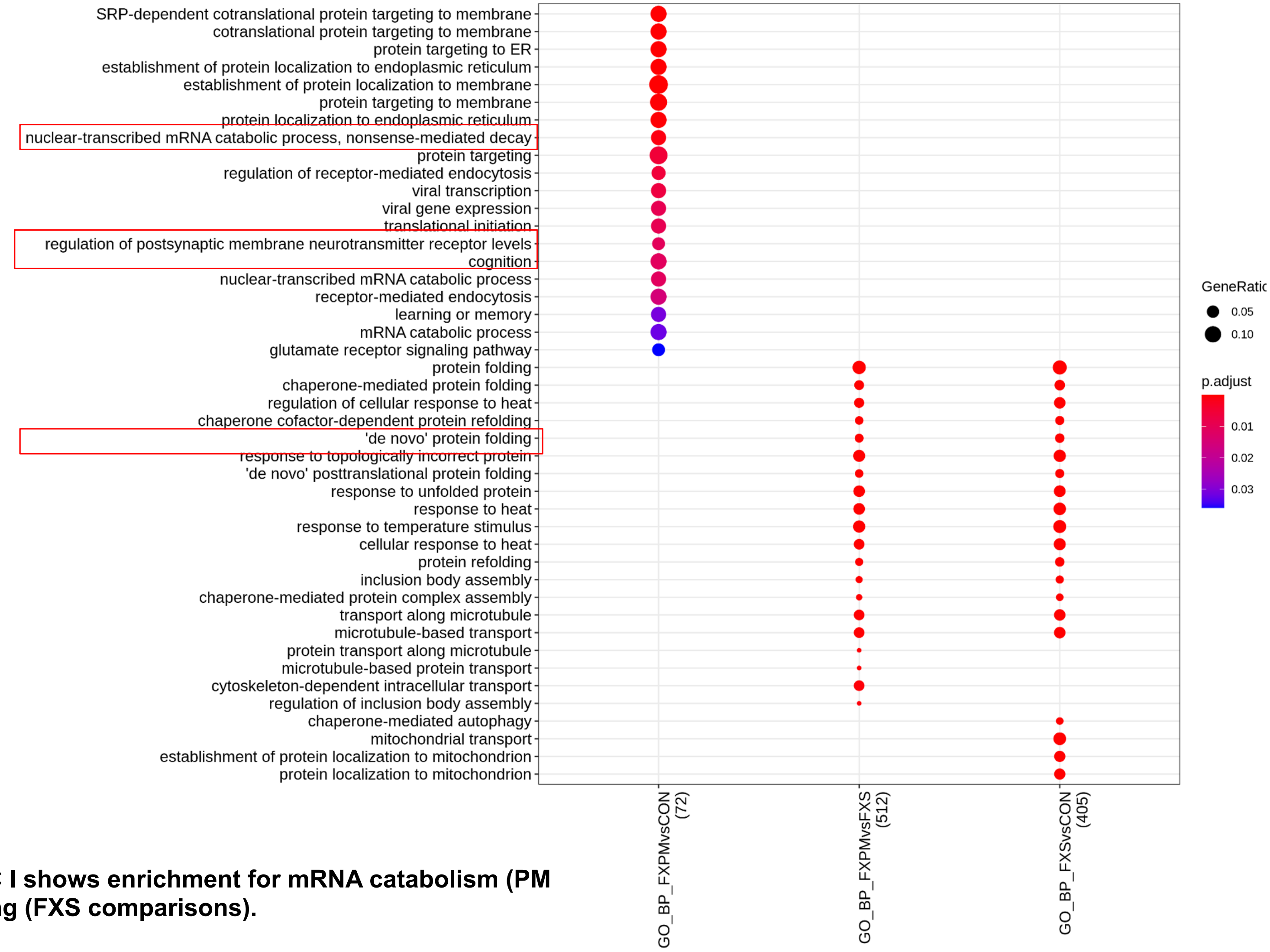

GO Biological Process for SV2C I shows enrichment for mRNA catabolism (PM comparisons) and protein folding (FXS comparisons).

Inh-SV2C I

KEGG

Top 20

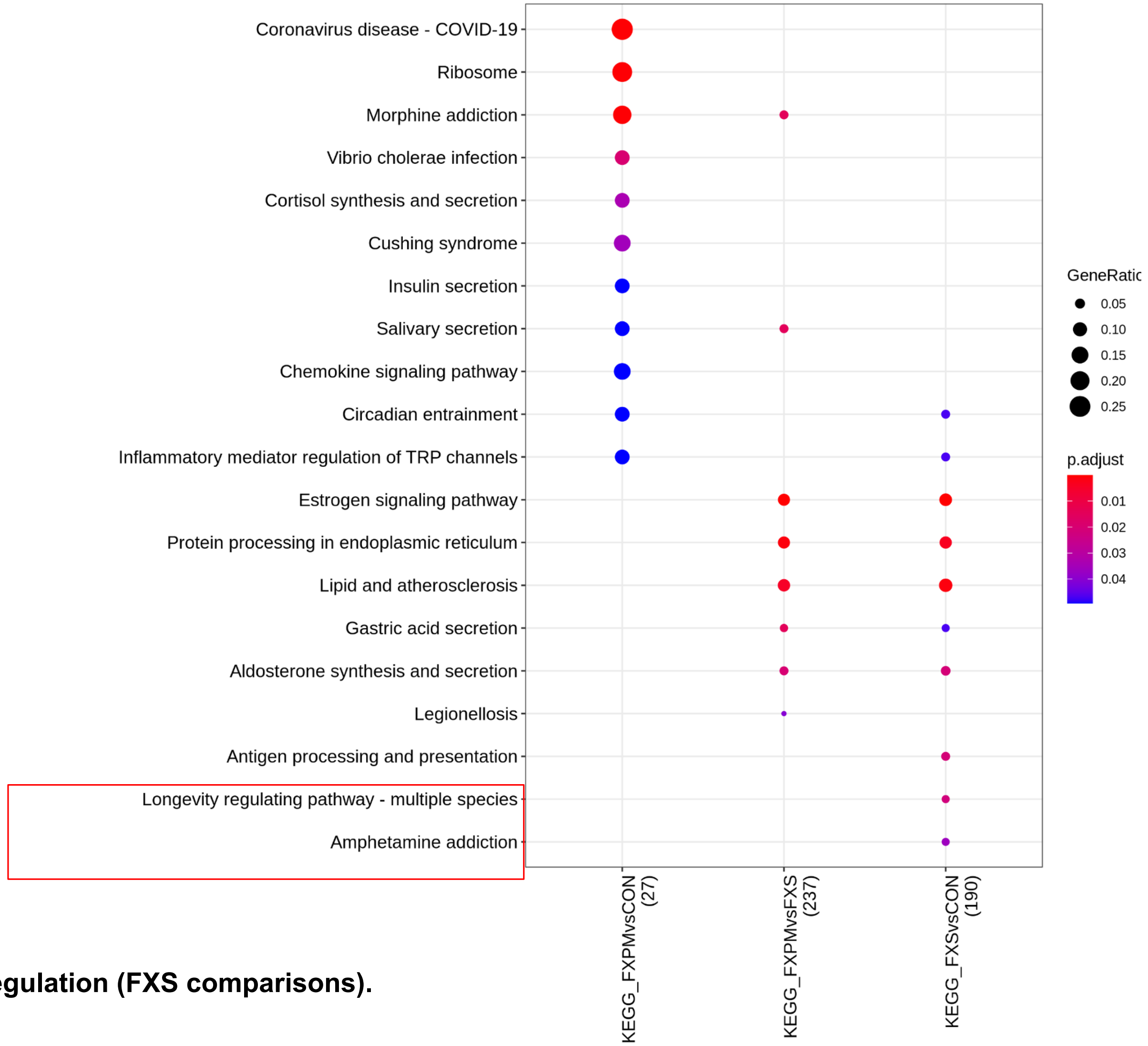

KEGG for SV2C I shows enrichment for longevity regulation (FXS comparisons).

Inh-SV2C II

GO_BP

Top 20

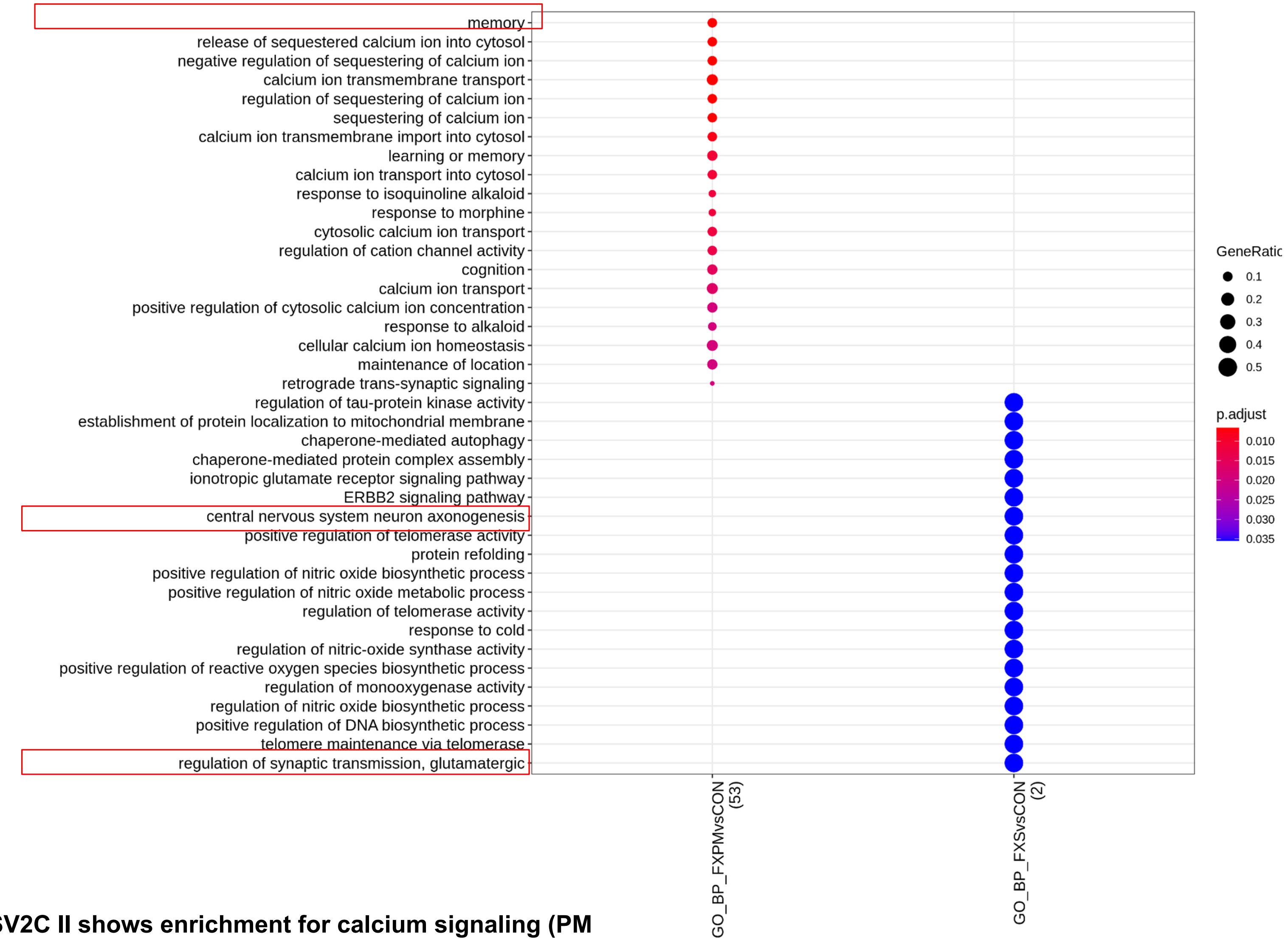

GO Biological Process for SV2C II shows enrichment for calcium signaling (PM comparisons) and synaptic transmission (FXS comparisons).

Inh-SV2C II

KEGG

Top 20

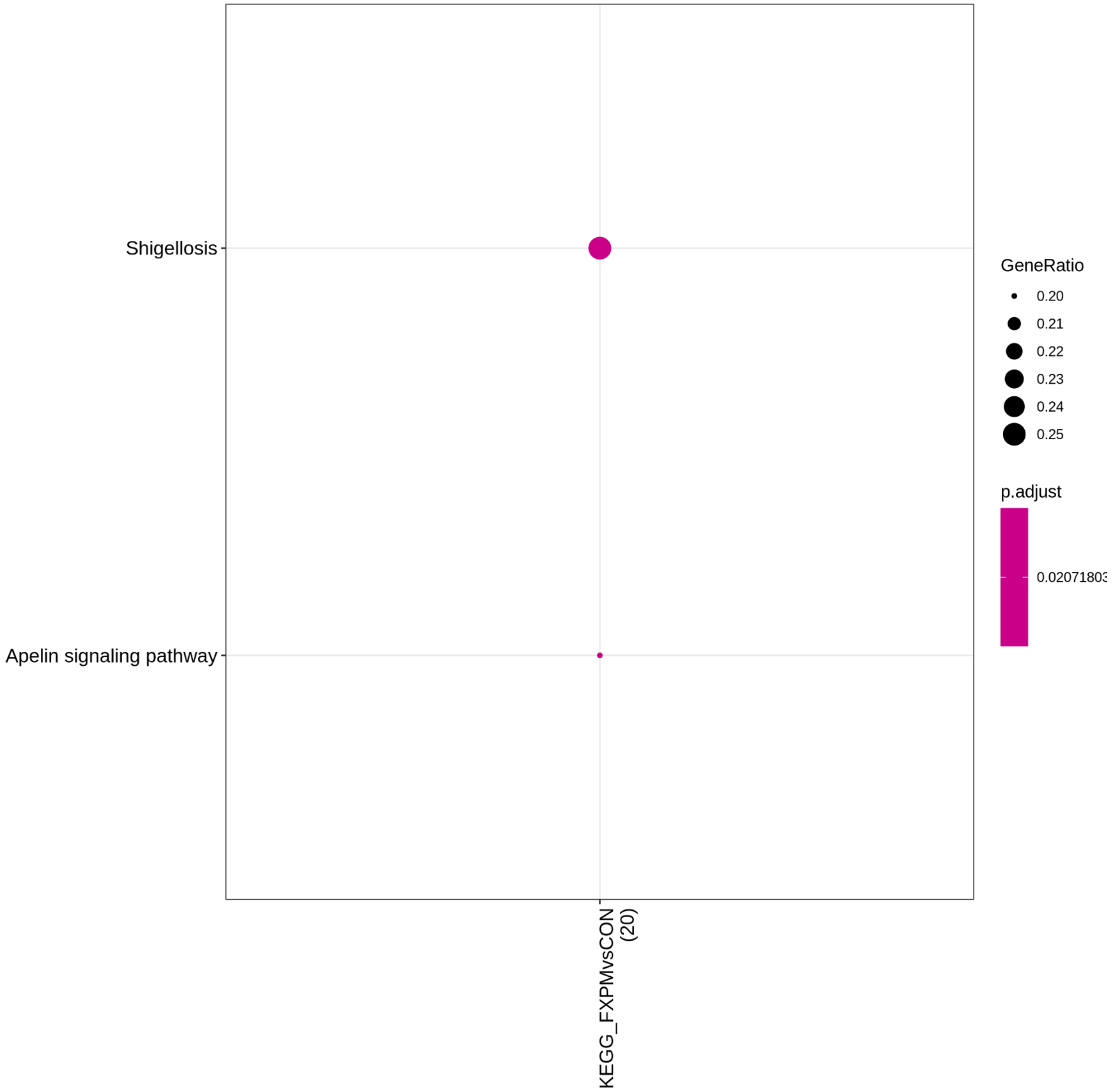

Inh-VIP

GO_BP

Top 20

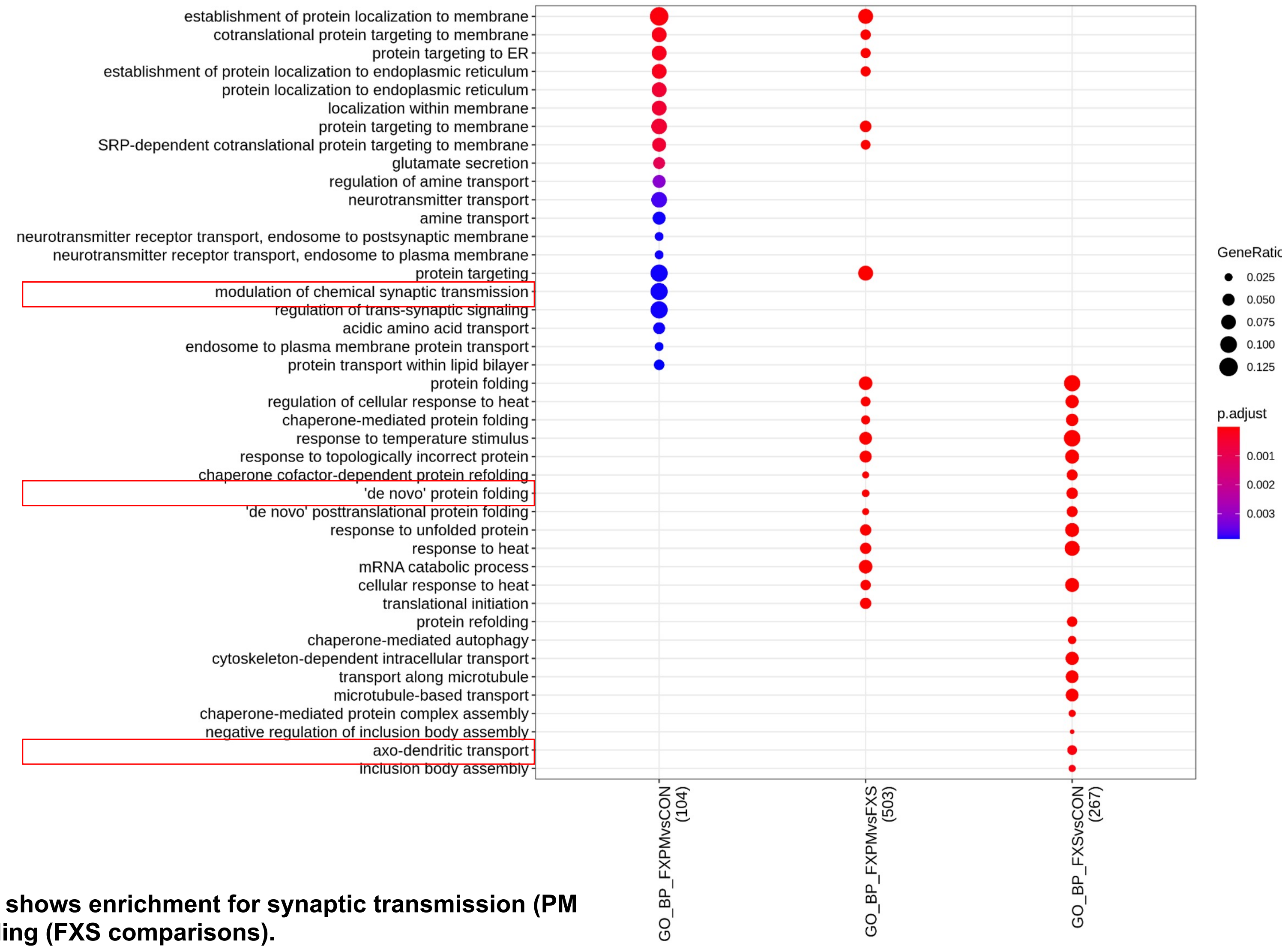

GO Biological Process for VIP shows enrichment for synaptic transmission (PM comparisons) and protein folding (FXS comparisons).

Inh-VIP

KEGG

Top 20

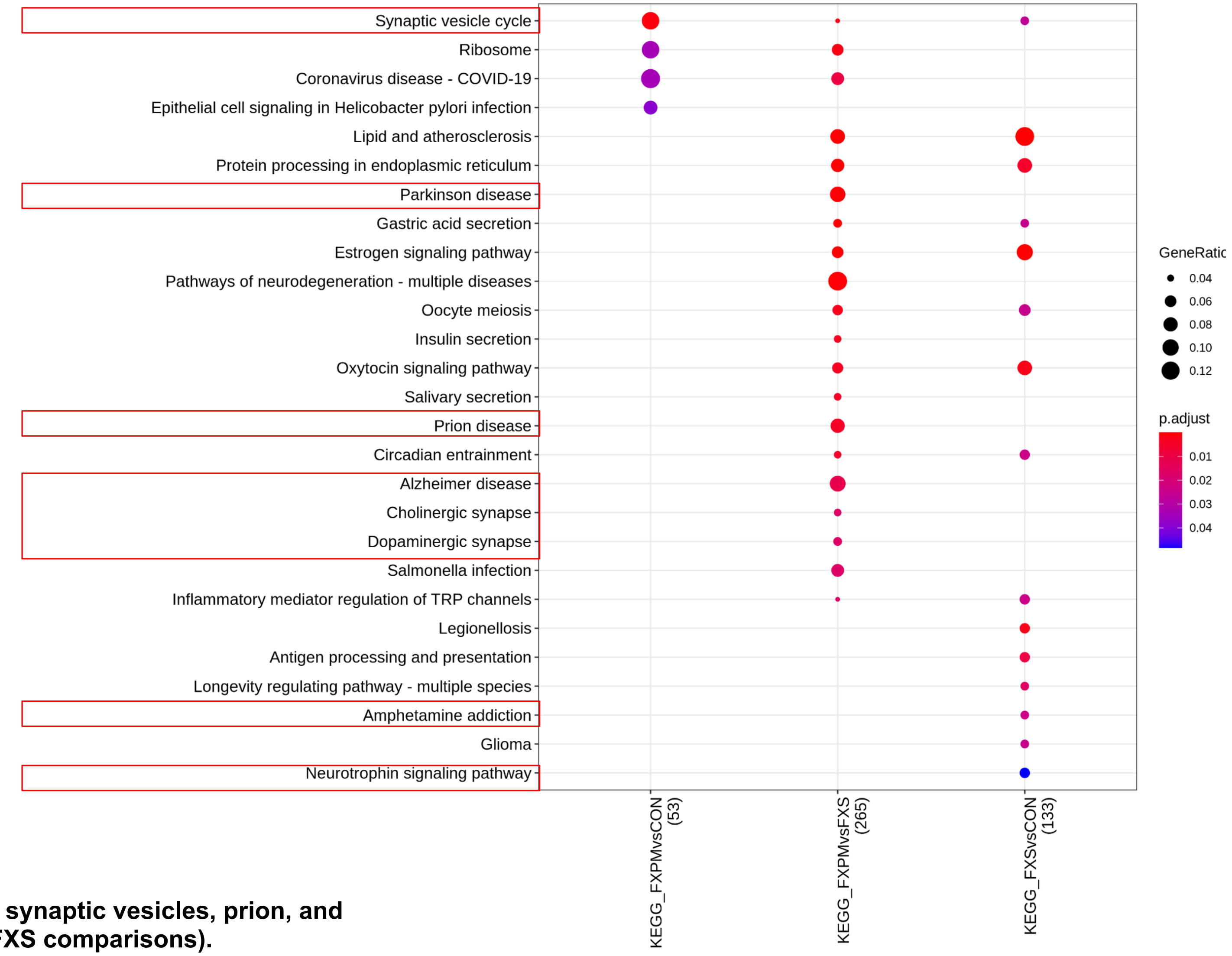

KEGG for VIP shows enrichment for synaptic vesicles, prion, and neurodegenerative terms (PM and FXS comparisons).

Microglia

GO_BP

Top 20

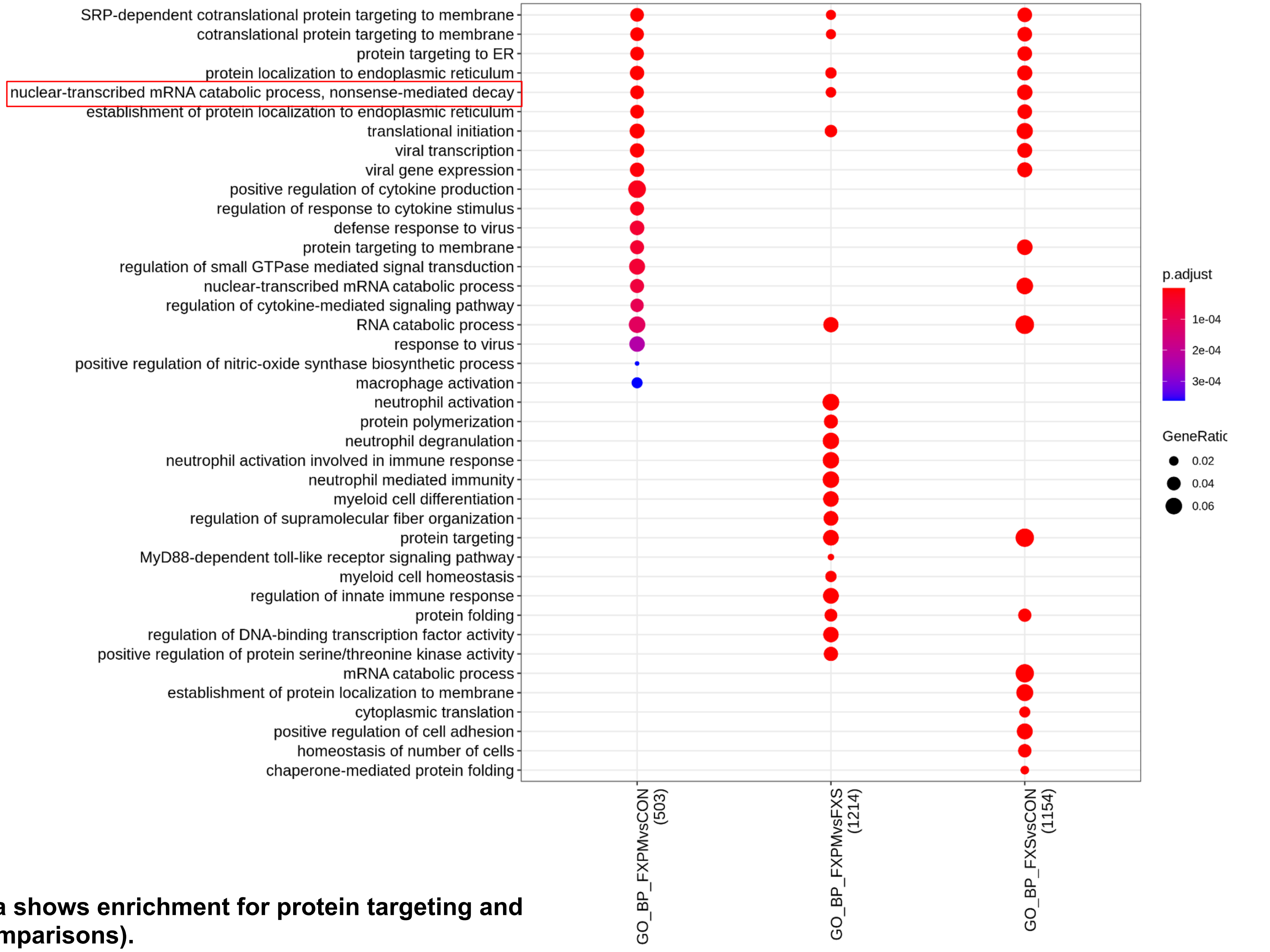

GO Biological Process for microglia shows enrichment for protein targeting and mRNA catabolism (PM and FXS comparisons).

Microglia

KEGG

Top 20

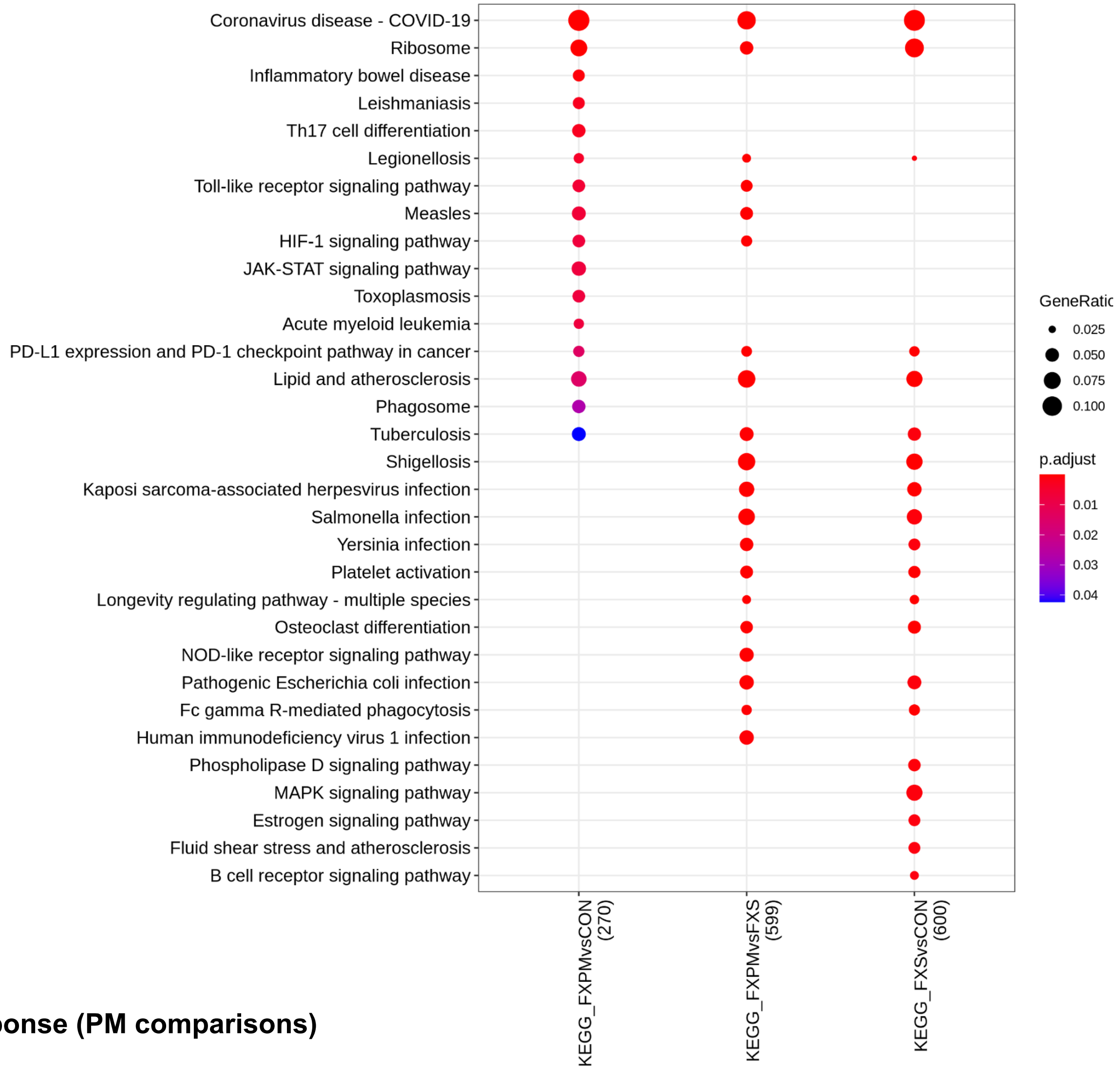

KEGG for microglia shows enrichment for infection response (PM comparisons) and MAPK signaling (FXS comparisons).

MOL

GO_BP

Top 20

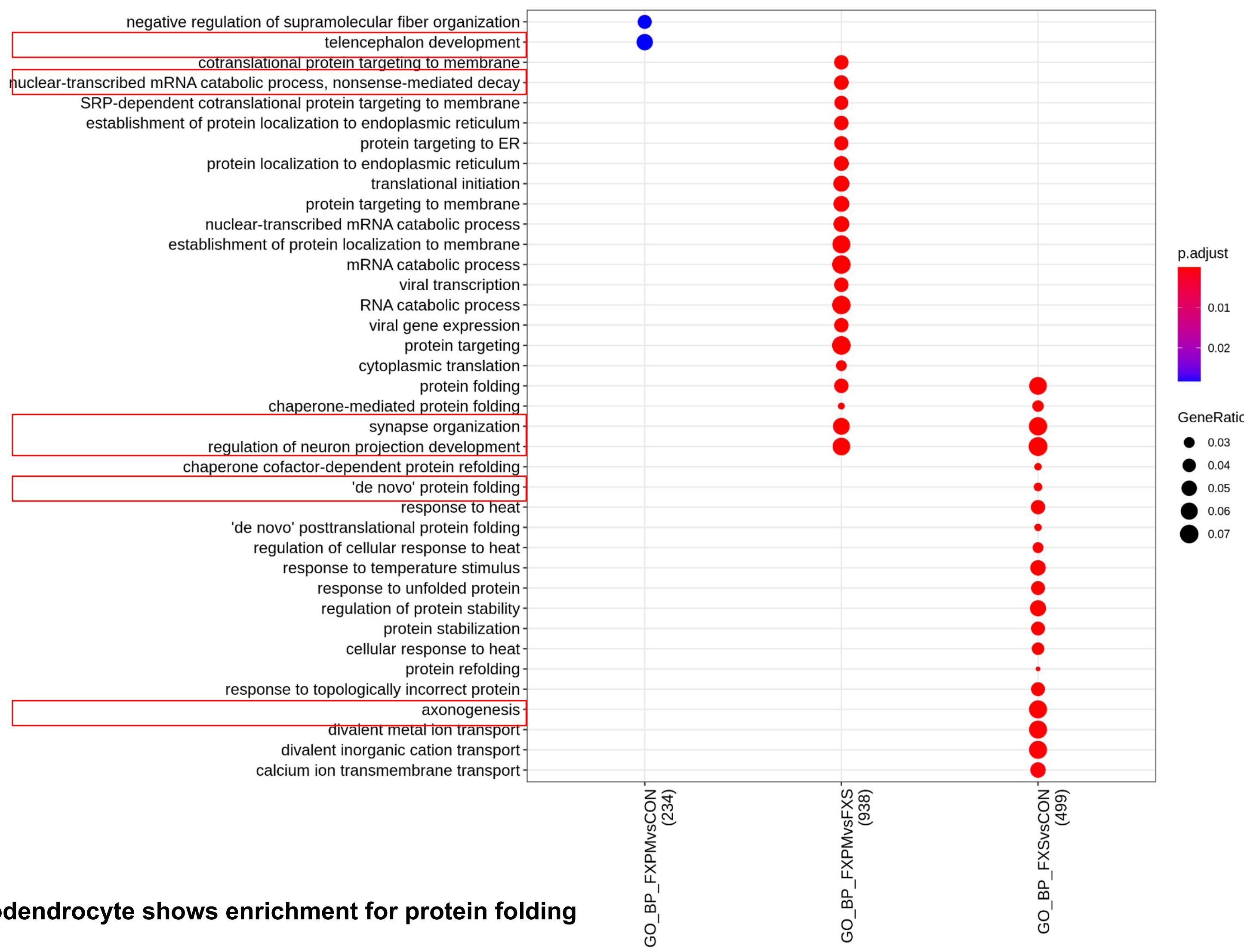

GO Biological Processes for oligodendrocyte shows enrichment for protein folding (FXS comparisons).

MOL

KEGG

Top 20

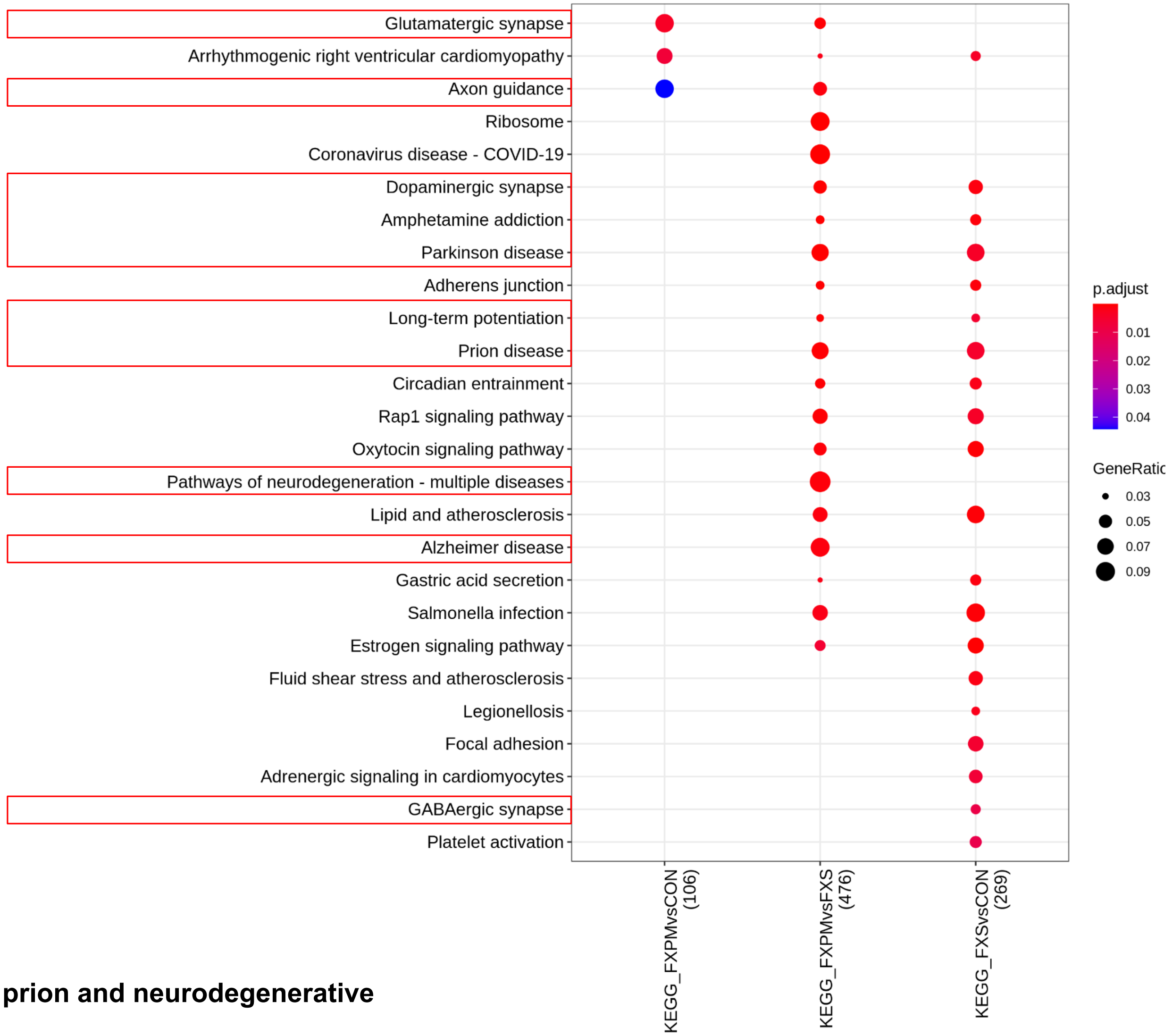

KEGG for oligodendrocyte shows enrichment for prion and neurodegenerative terms (FXS comparisons).

Neu L4 I

GO_BP

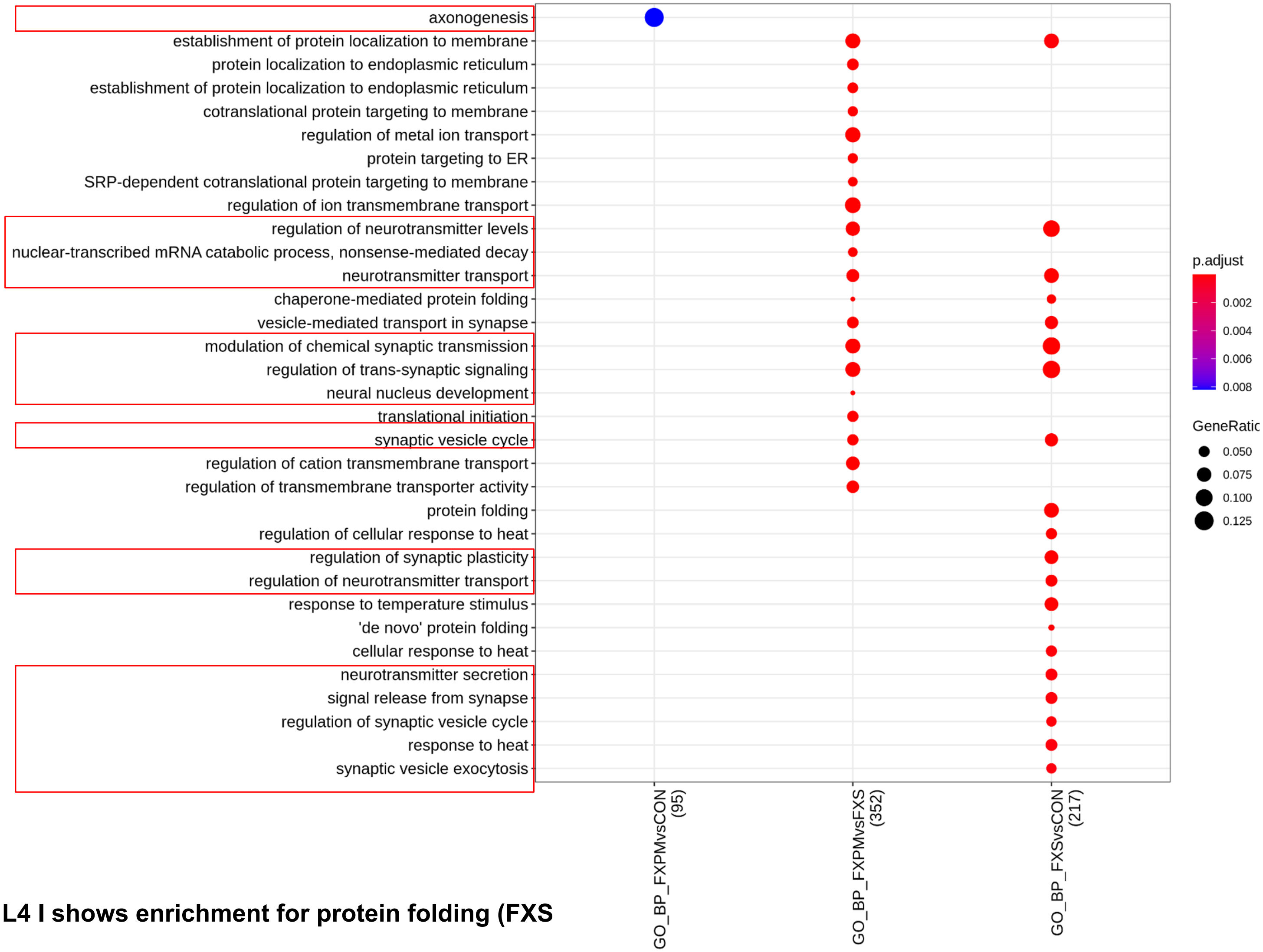

GO Biological Processes for Neu L4 I shows enrichment for protein folding (FXS comparisons).

Neu L4 I

KEGG

Top 20

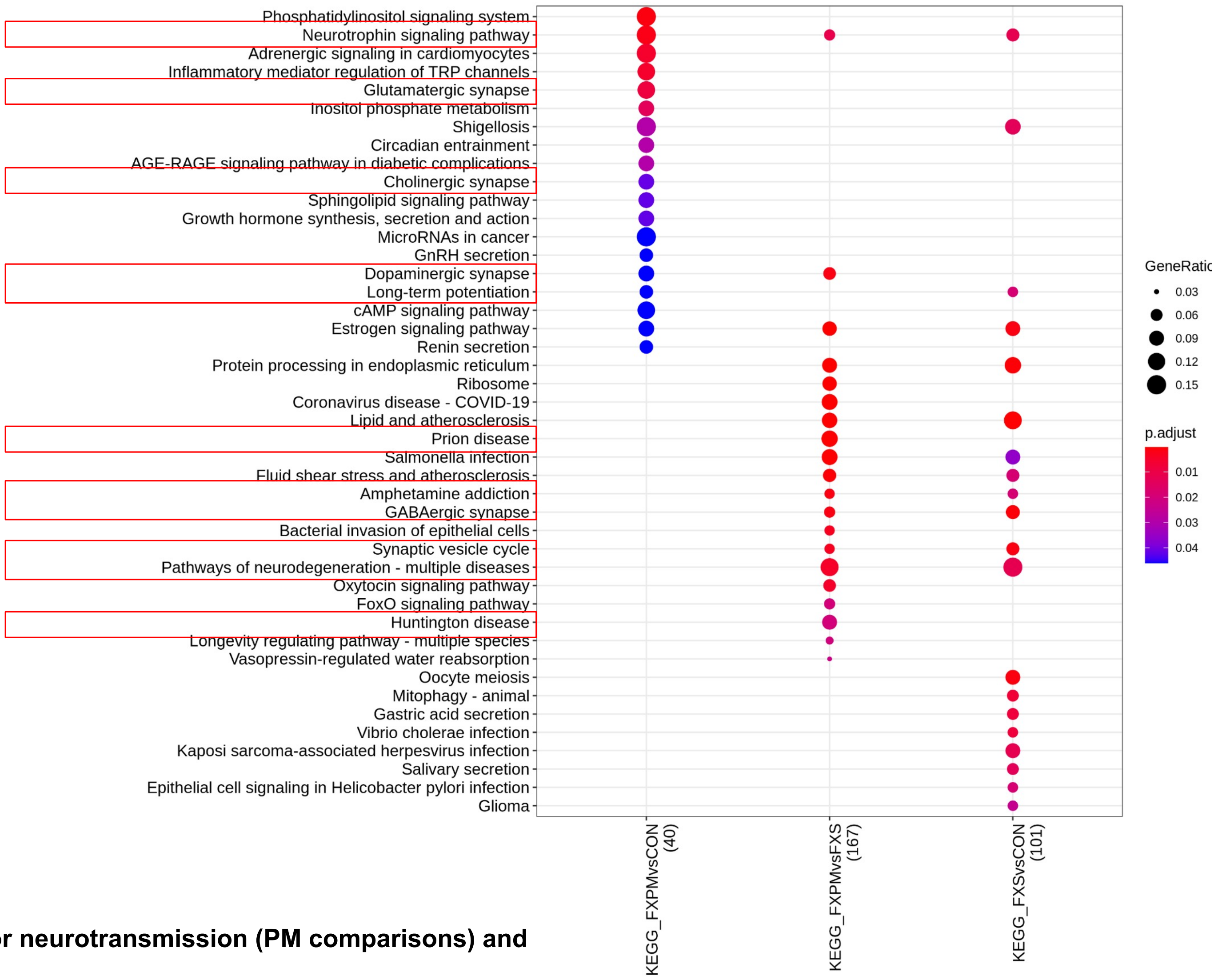

KEGG for Neu L4 I shows enrichment for neurotransmission (PM comparisons) and infection (FXS comparisons).

Neu L4 II

GO_BP

Top 20

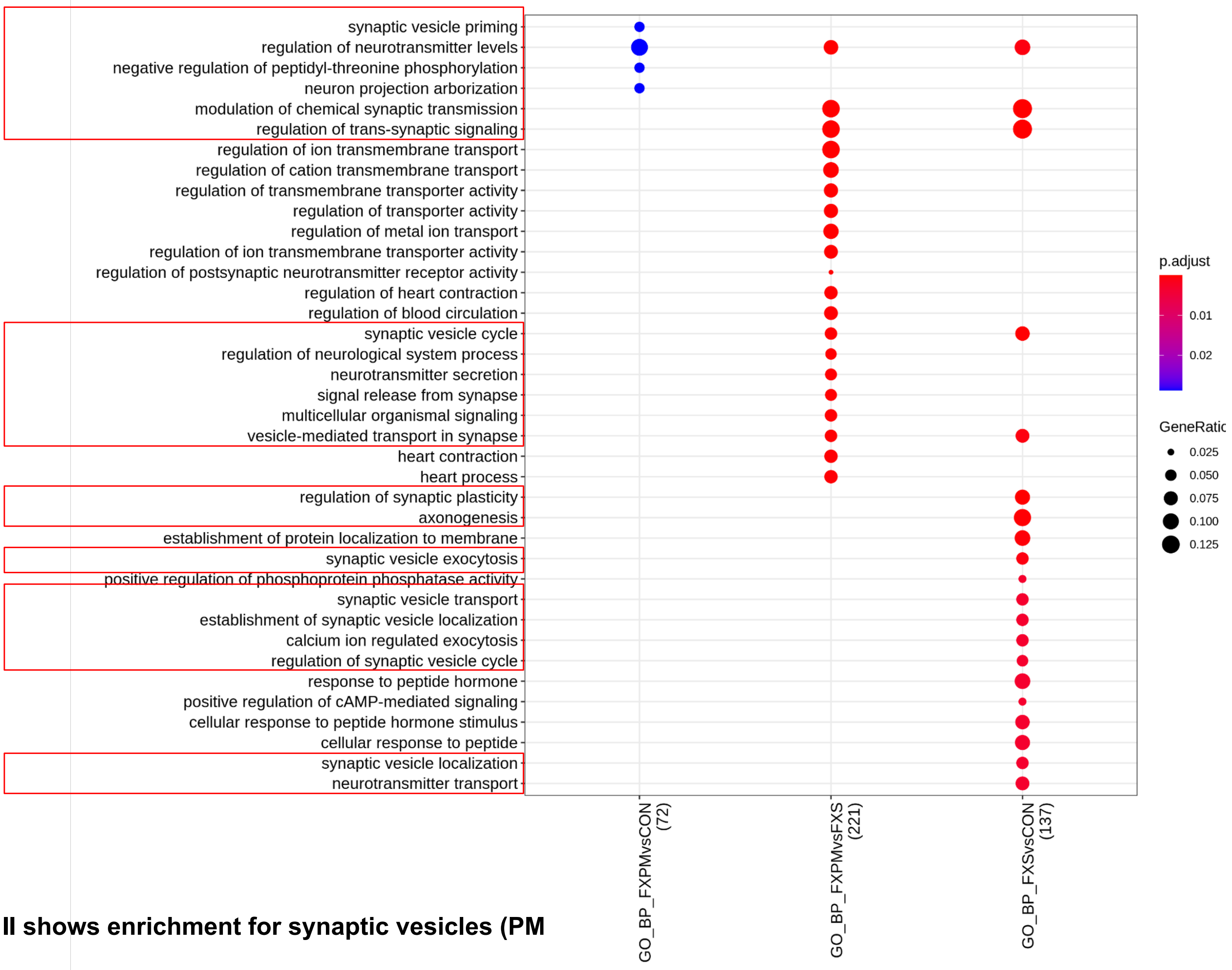

GO Biological Processes for Neu L4 II shows enrichment for synaptic vesicles (PM and FXS comparisons).

Neu L4 II

KEGG

Top 20

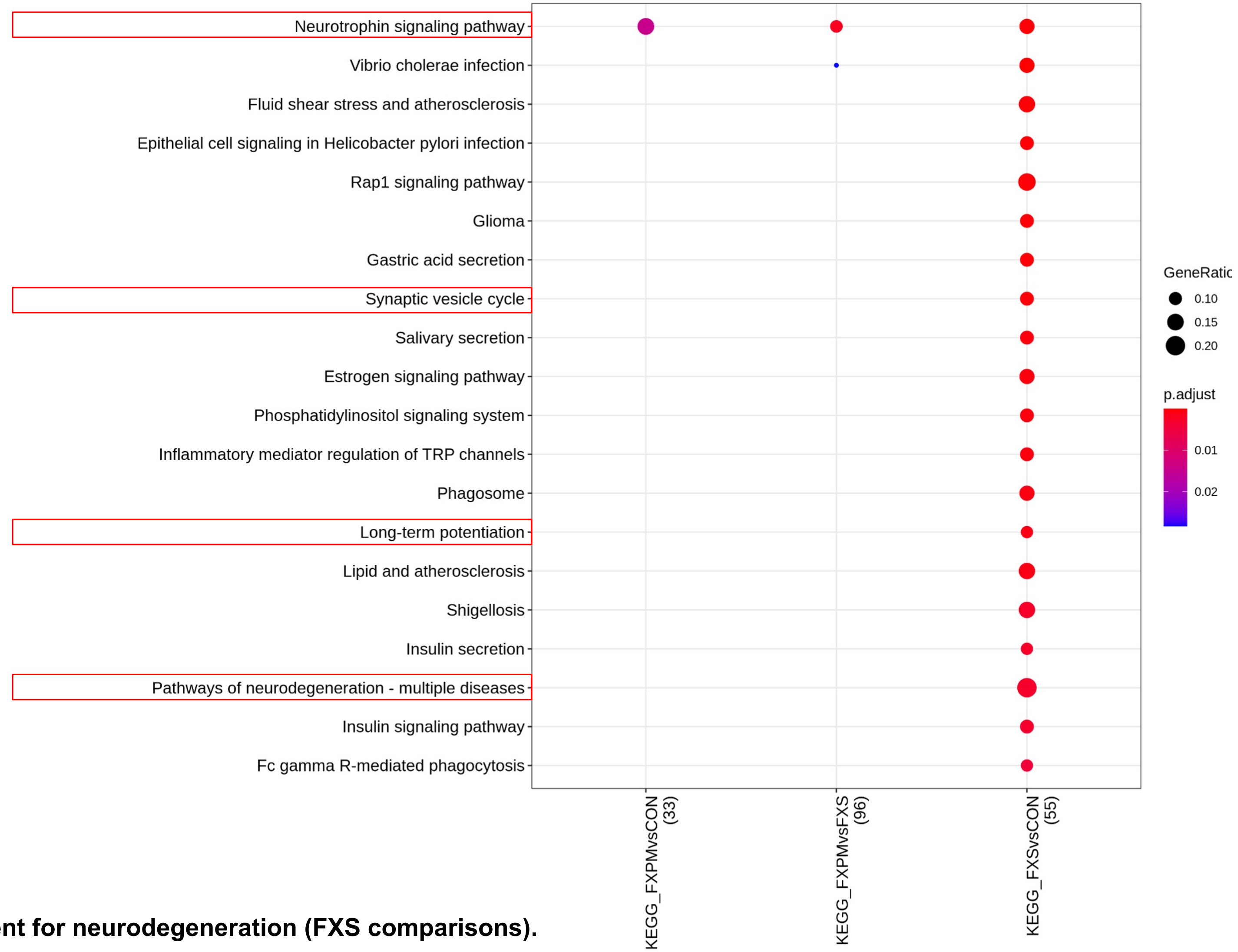

KEGG for Neu L4 II shows enrichment for neurodegeneration (FXS comparisons).

Neu NRGN

GO_BP

Top 20

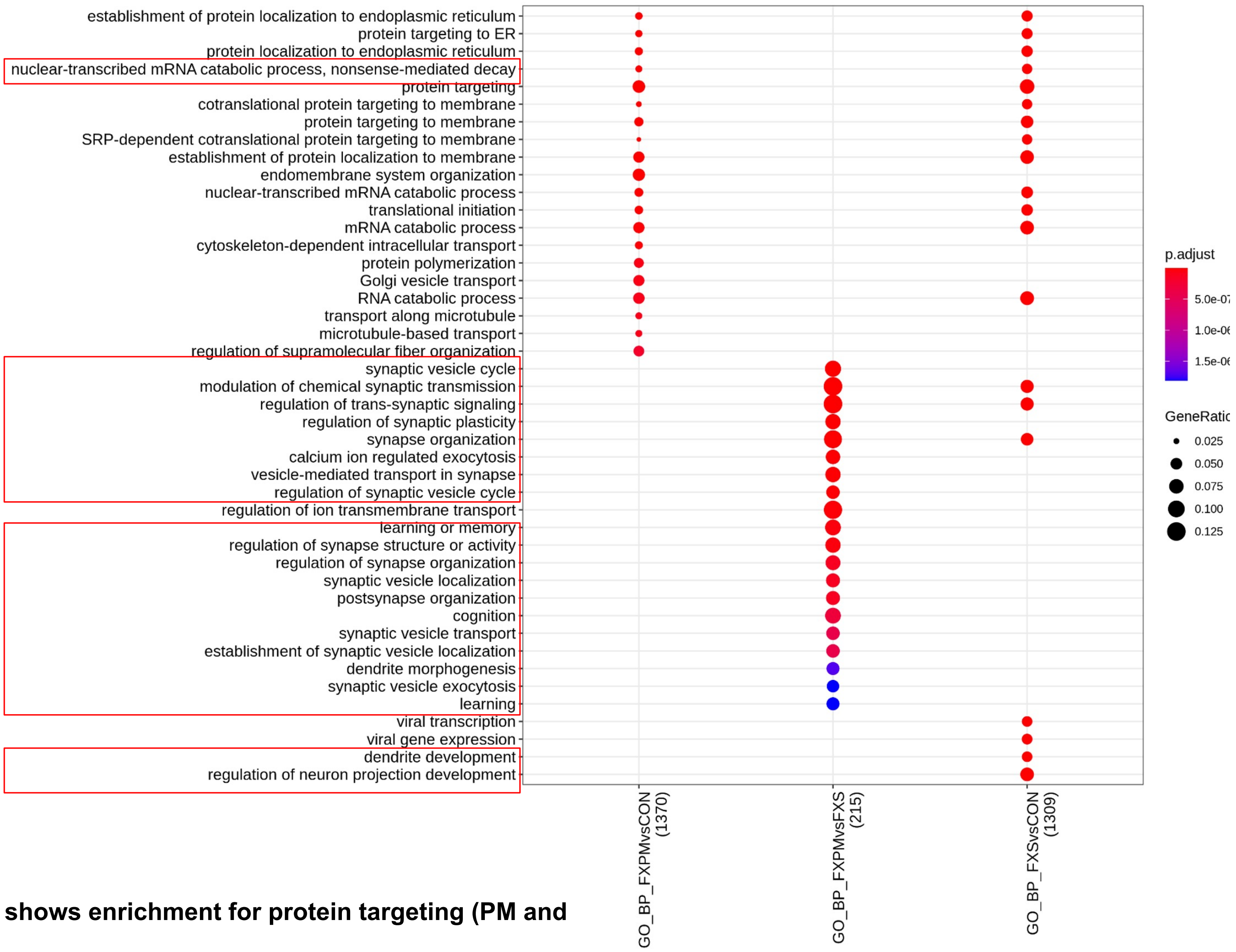

GO Biological Processes for NRGN shows enrichment for protein targeting (PM and FXS comparisons).

Neu NRGN

KEGG

Top 20

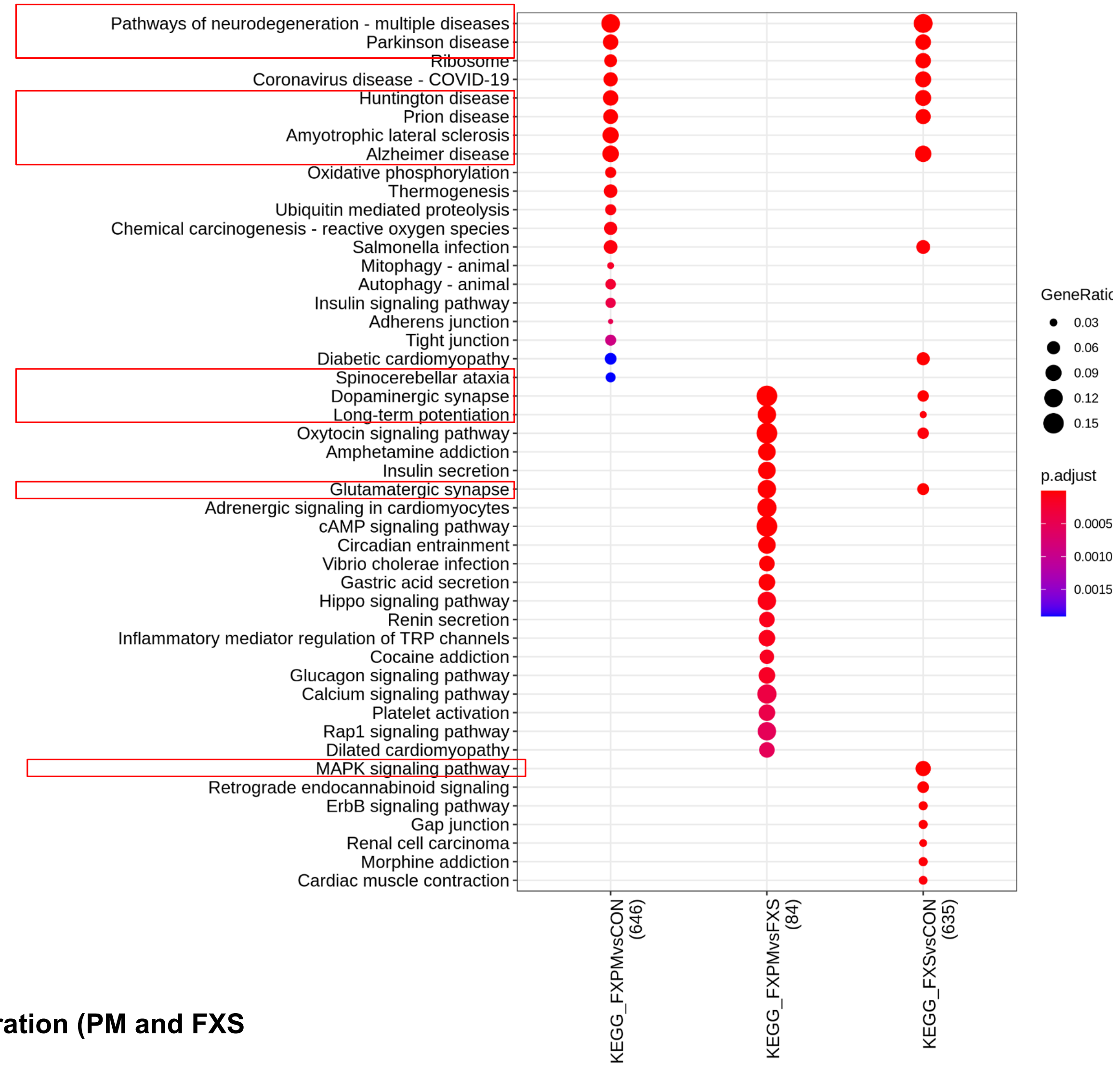

KEGG for NRGN shows enrichment for neurodegeneration (PM and FXS comparisons).

Neu NTNG2

GO_BP

Top 20

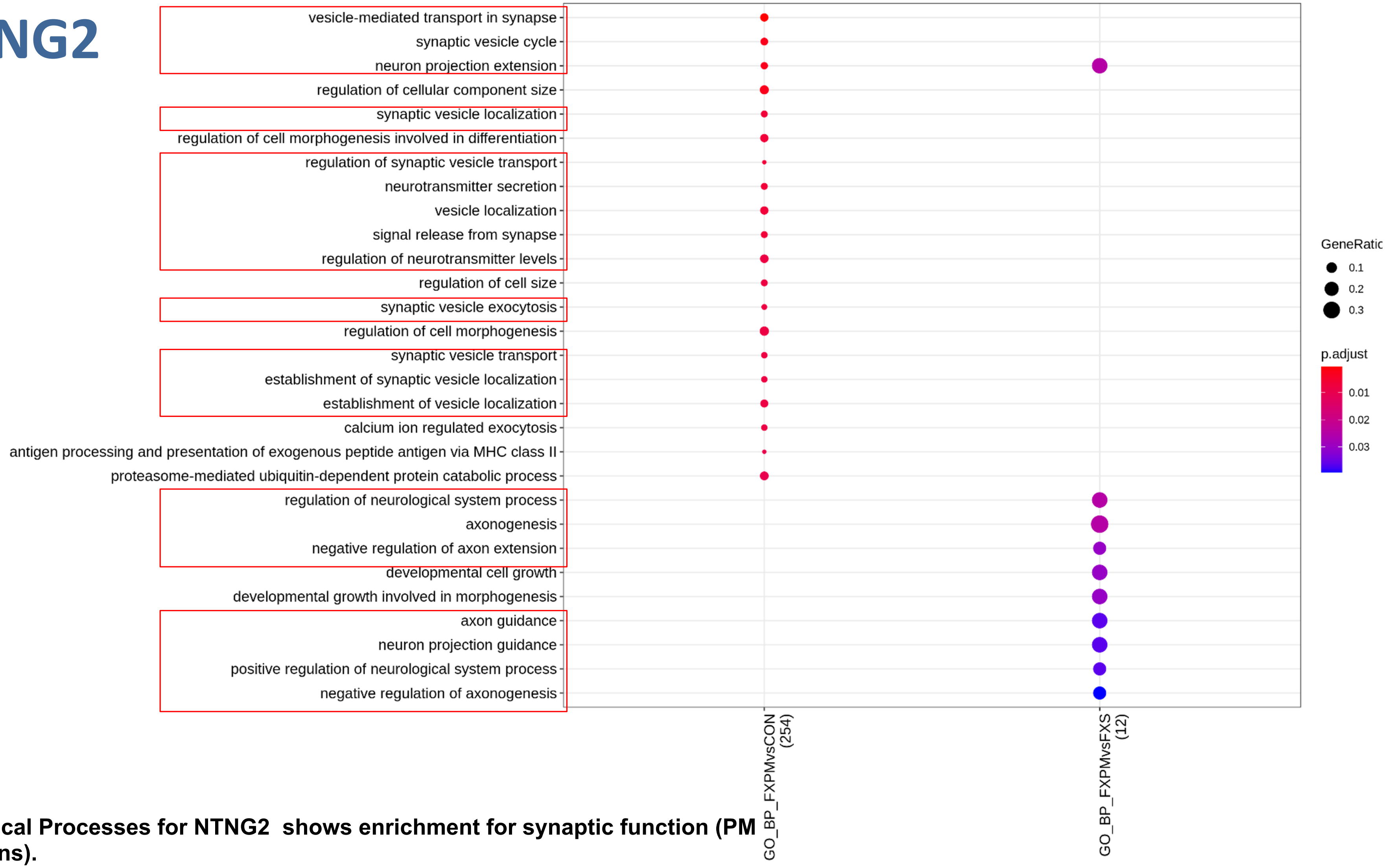

GO Biological Processes for NTNG2 shows enrichment for synaptic function (PM comparisons).

Neu NTNG2

KEGG

Top 20

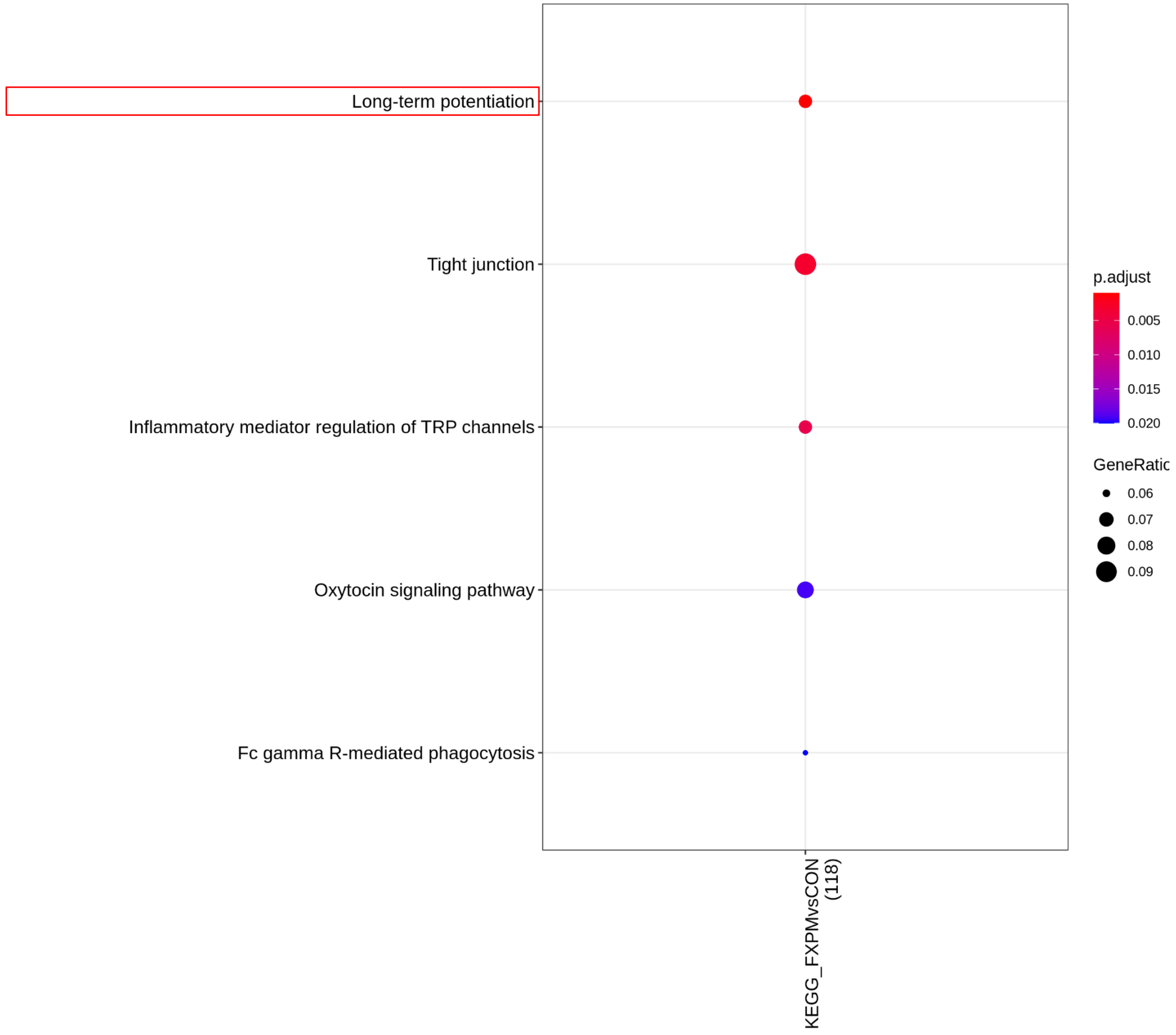

OL I

GO_BP

Top 20

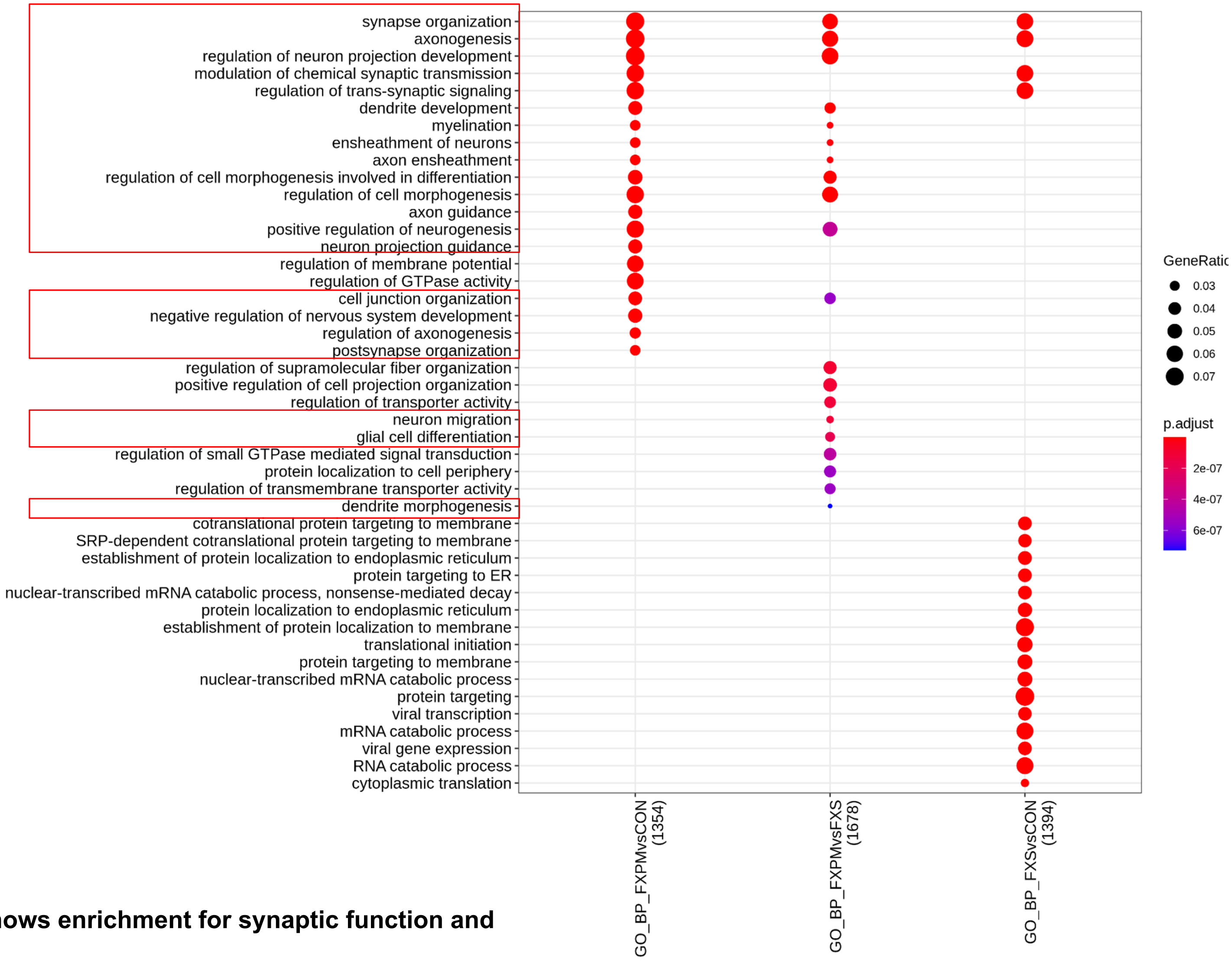

GO Biological Processes for OLI shows enrichment for synaptic function and myelination (PM comparisons).

OL I

KEGG

Top 20

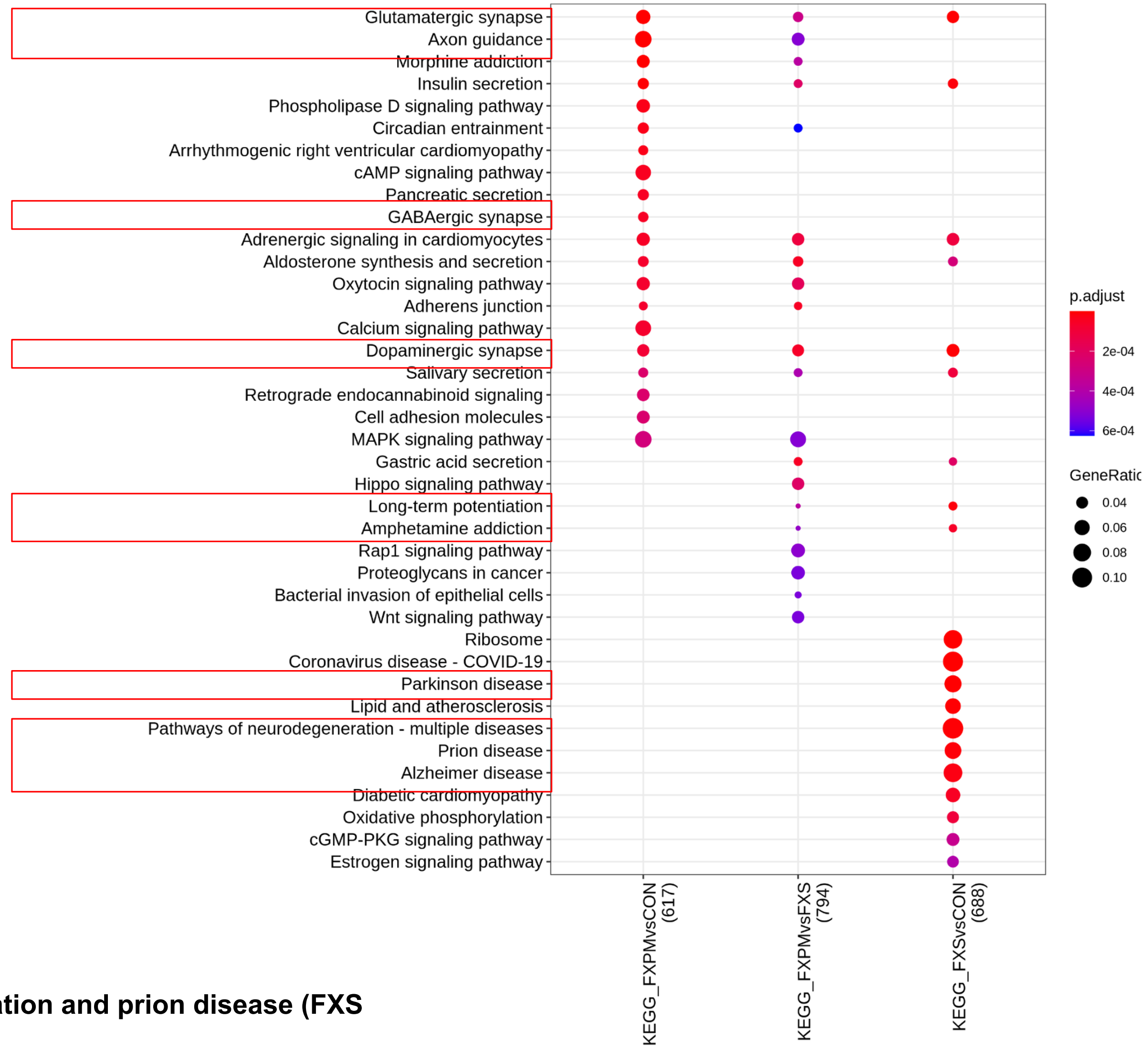

KEGG for OLI shows enrichment for neurodegeneration and prion disease (FXS comparisons).

OL II

GO_BP

Top 20

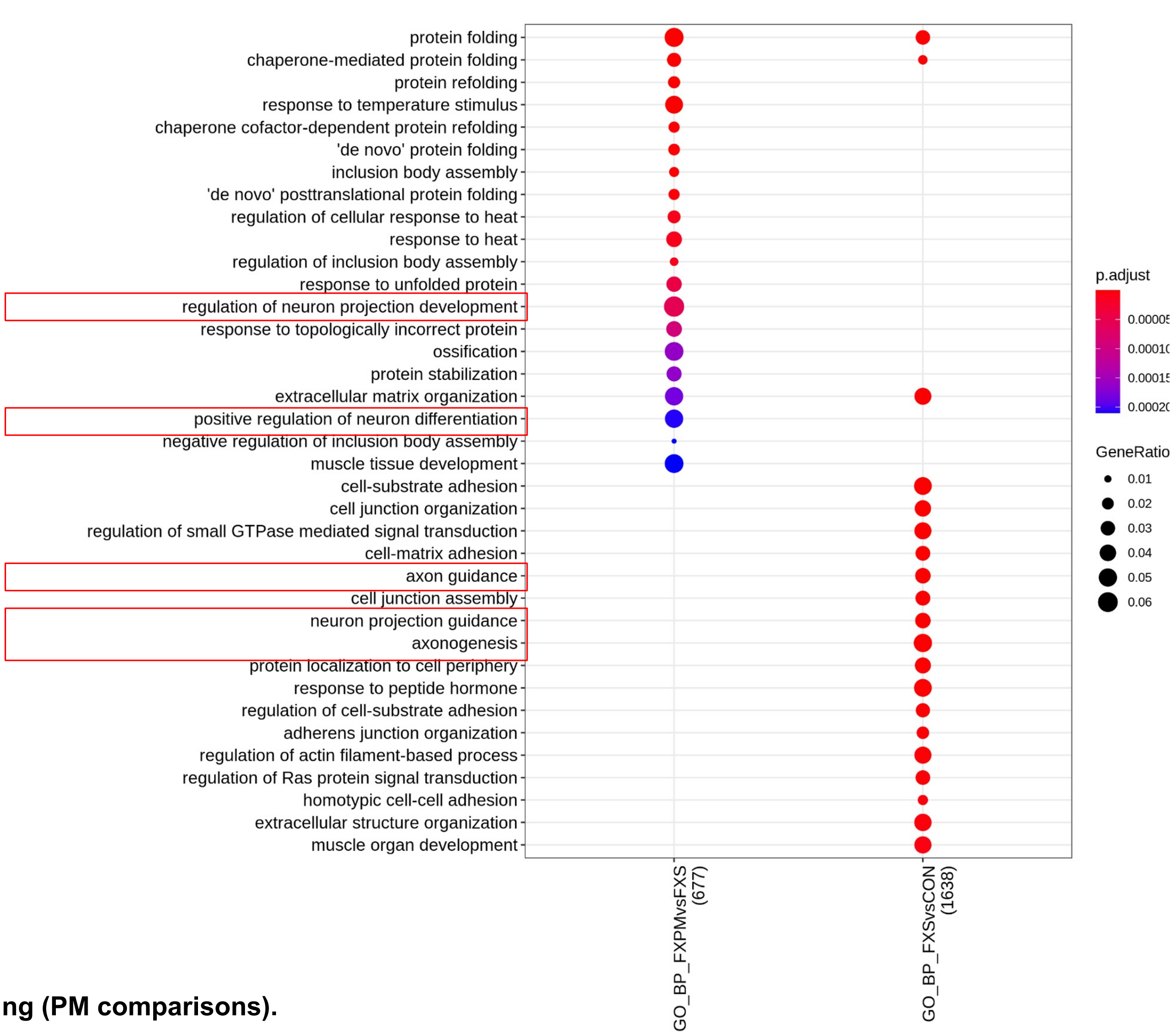

GO for OLII shows enrichment for protein folding (PM comparisons).

OL II

KEGG

Top 20

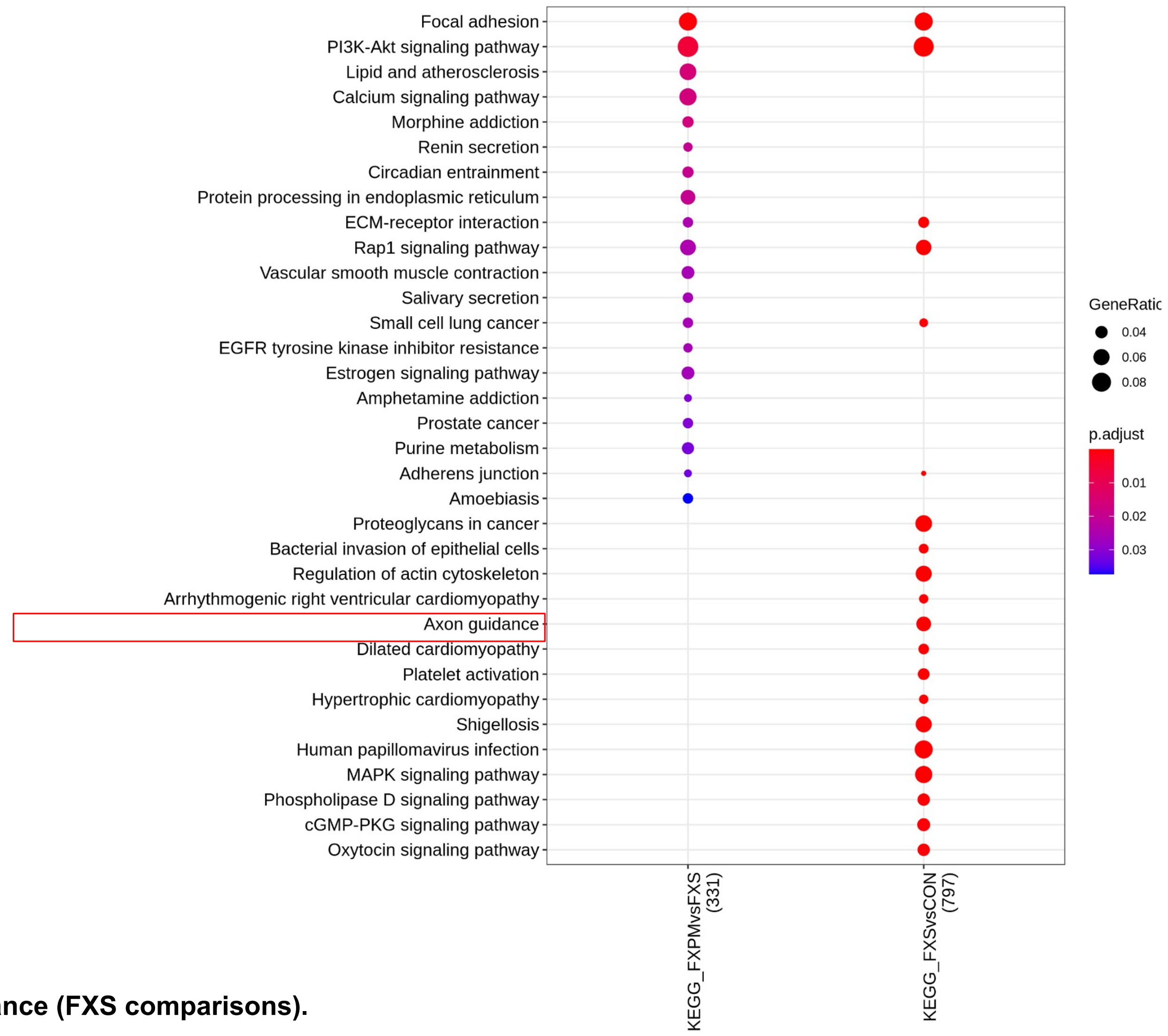

KEGG for OLII shows enrichment for axon guidance (FXS comparisons).

OPC

GO_BP

Top 20

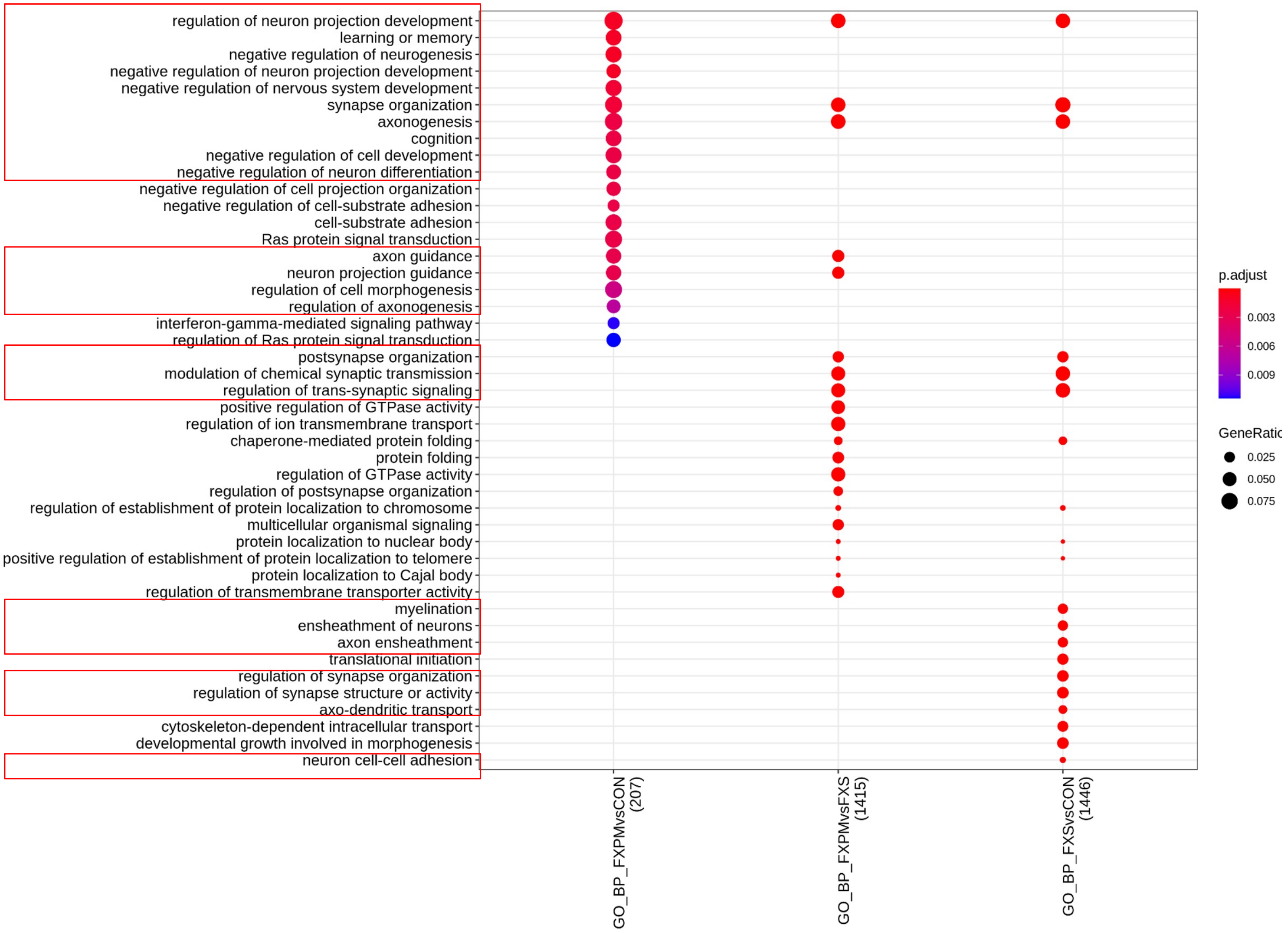

GO for OPC shows enrichment for cognition (PM comparisons) and myelination (FXS comparisons).

OPC

KEGG

Top 20

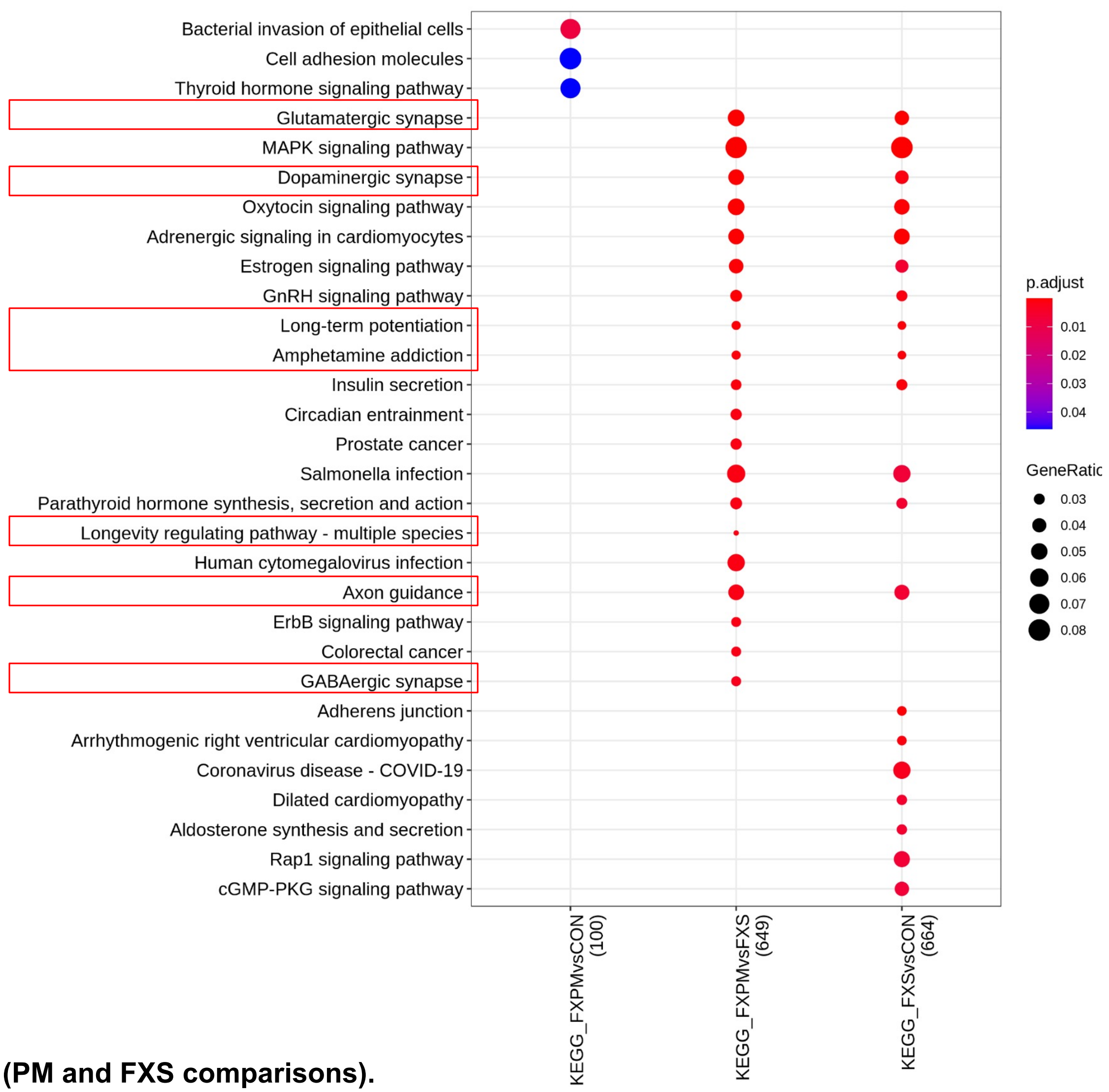

KEGG for OPC shows enrichment for MAPK signaling (PM and FXS comparisons).

L2/3-4

GO BP

Top 20

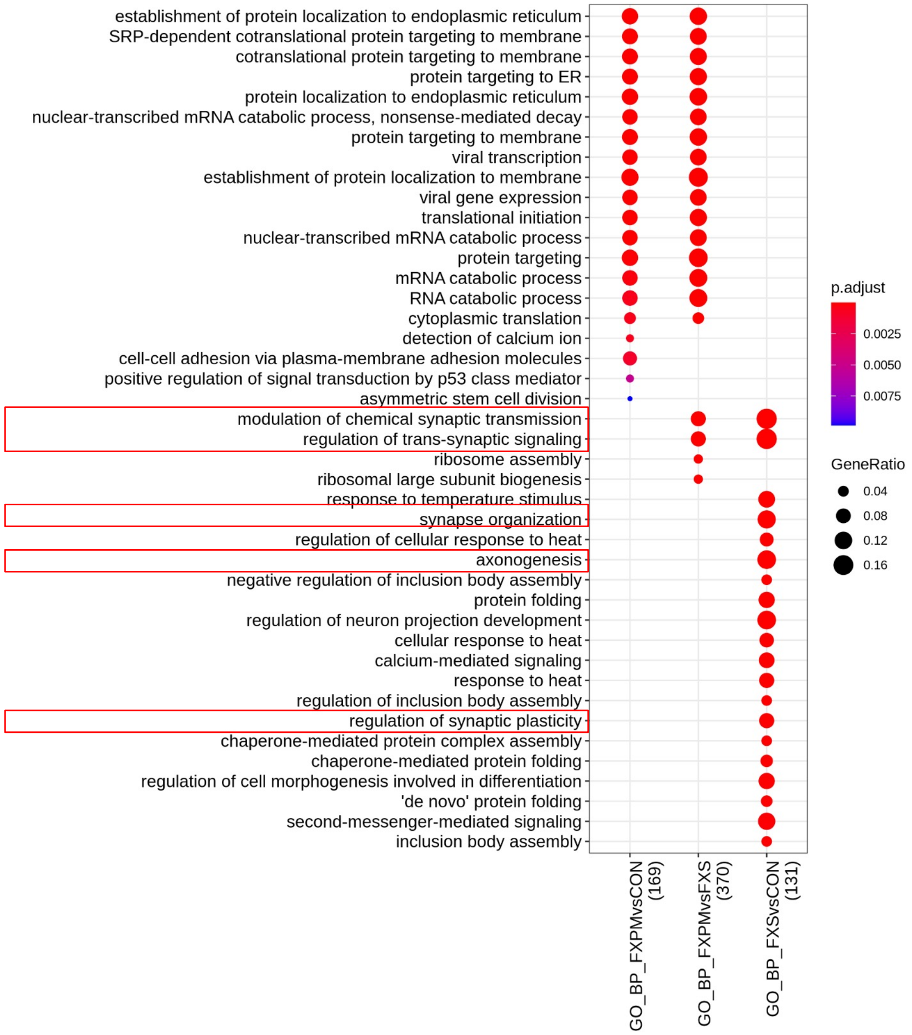

GO for L2/3-4 shows enrichment for protein targeting (PM comparisons).

L2/3-4

KEGG

Top 20

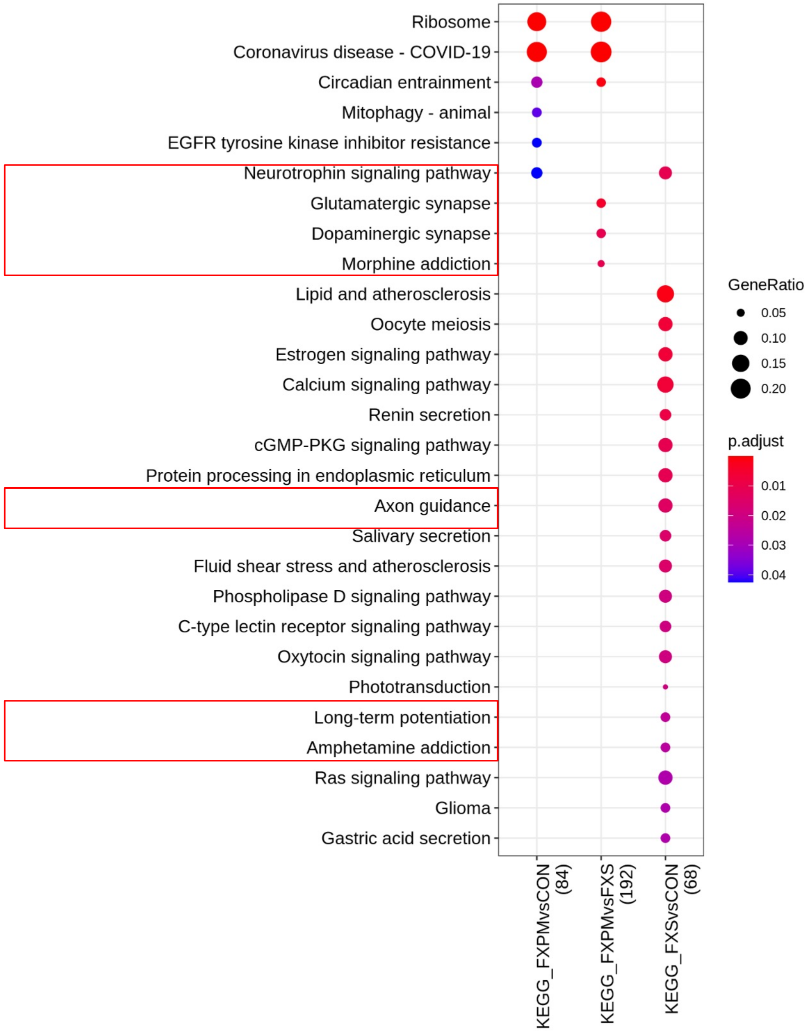

KEGG for L2/3-4 shows enrichment for axon guidance (FXS comparisons).

L5/6 I

GO BP

Top 20

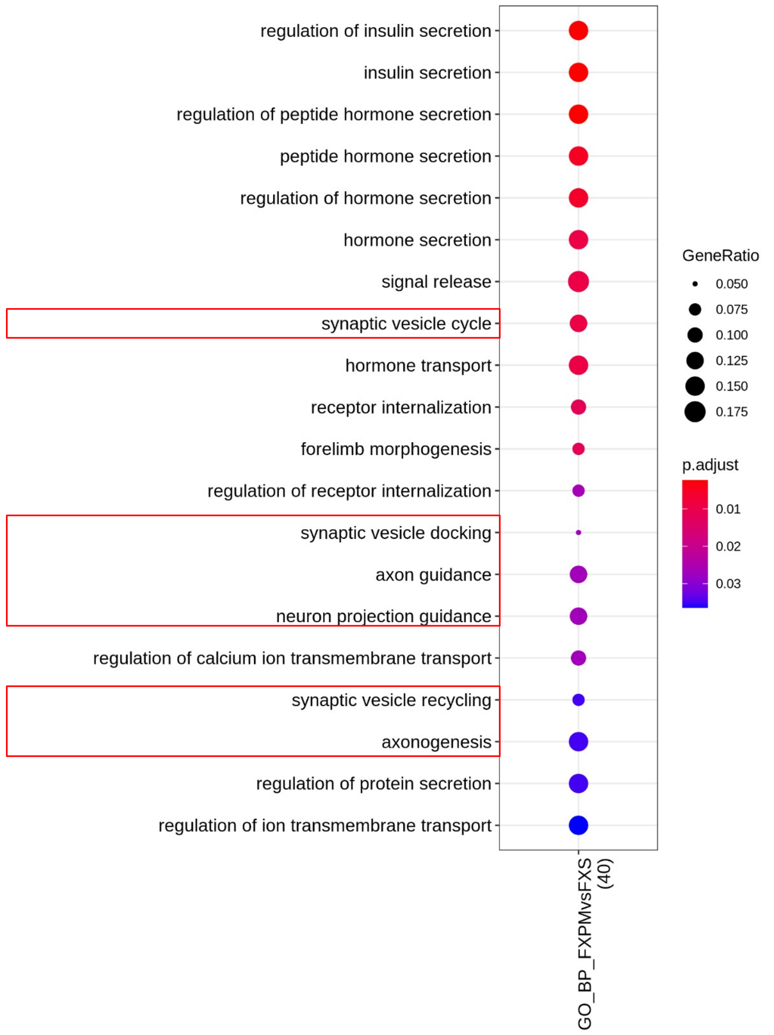

L5/6 I

KEGG

Top 20

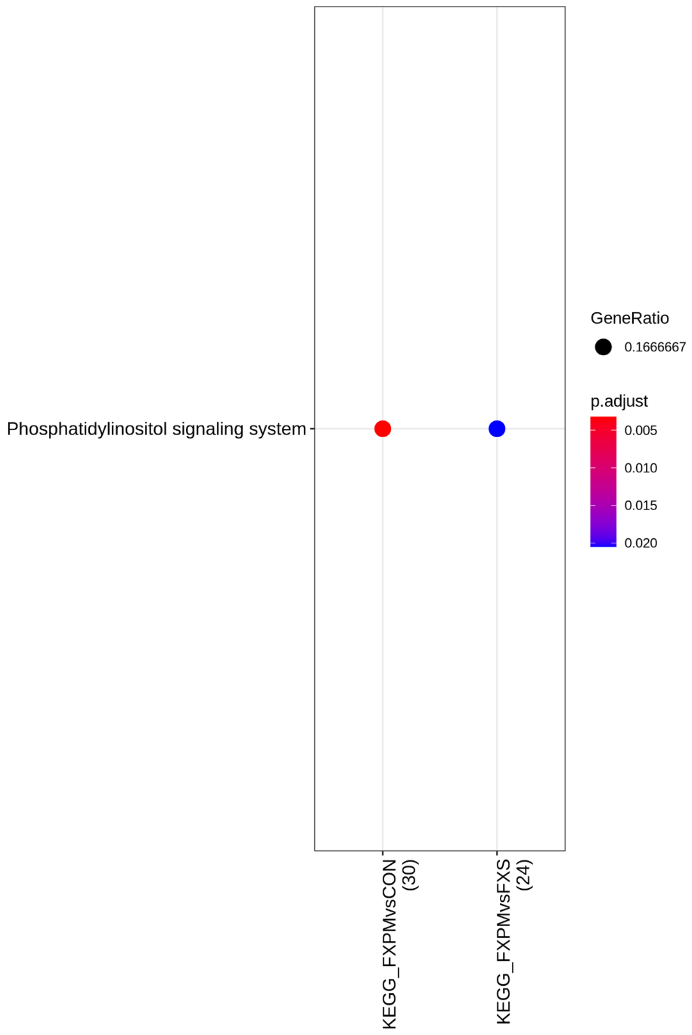

L5/6 II

GO BP

Top 20

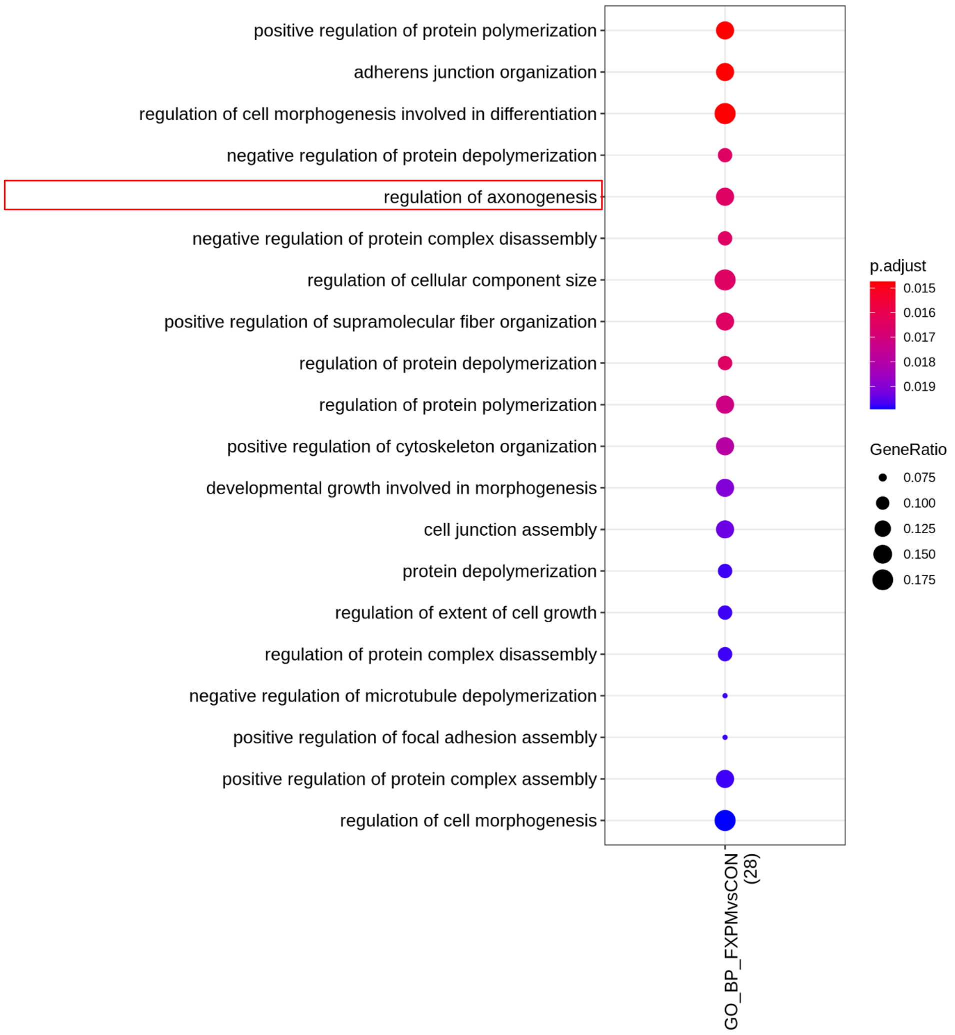

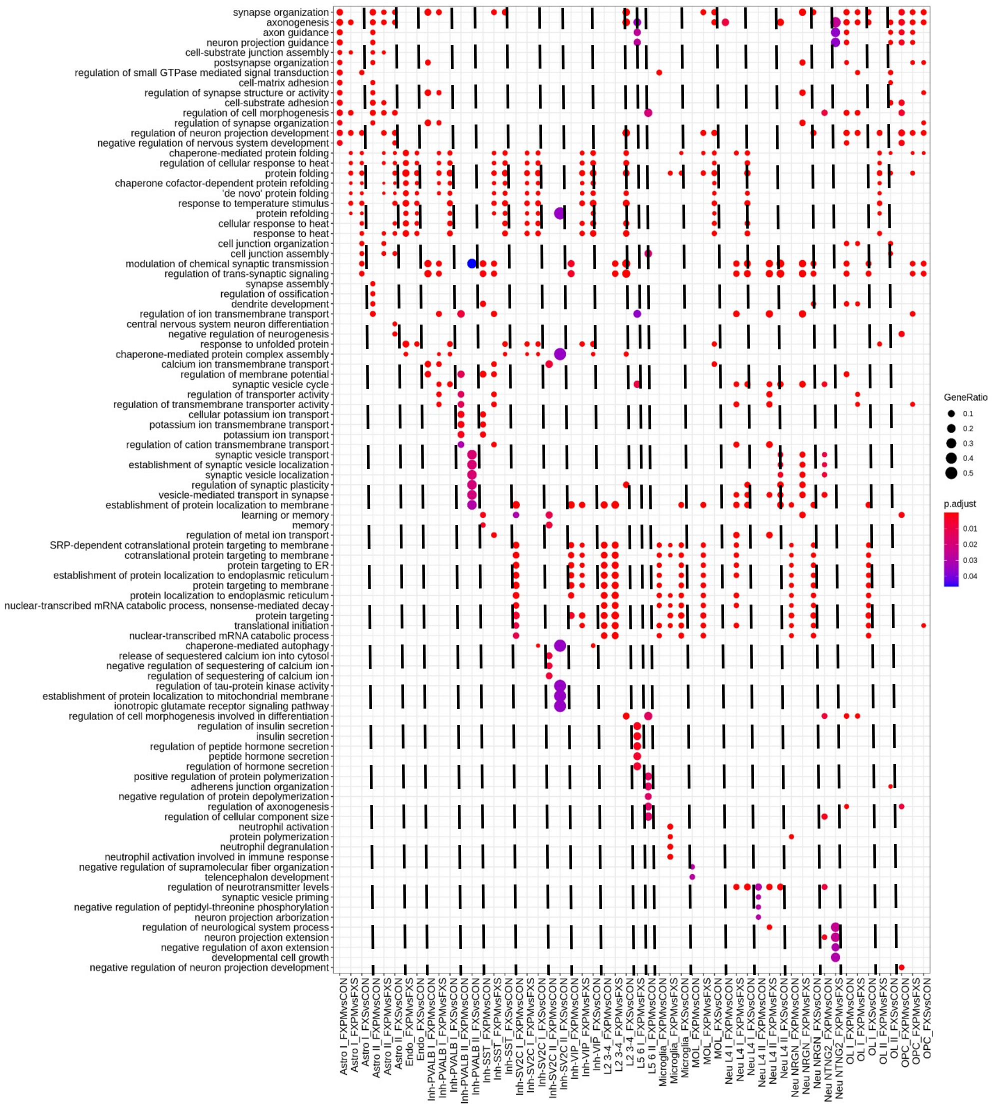

GO summary for cortex

Supplemental Methods: Analysis

For analysis of demographic data, one premutation case that was extremely aged is listed here as 89+ to ensure sample de-identification and sample points were removed from any graphs presented here to ensure de-identification. For premutation cluster proportion analysis, we used linear regression to determine the effect of both age and premutation status on cluster proportions. This approach is conservative, given our small sample size. We note that one young control sample had a high proportion of the committed progenitor OL I and one premutation sample demonstrated higher than expected OPC number. Additionally, the FXS case with the gene deletion demonstrated an unusually high presence of OLII.

Differential expression analysis was done with the FindMarkers functionality in Seurat (1) using settings of MAST test, padj < 0.05, and logfc.threshold = 0.25 for all cell clusters. A priori we calculated that 400 cells/condition cluster are required to detect 80% of differentially expressed genes with a false discovery rate of 5%. Thus, we are underpowered for rare cell types such as endothelial cells and Purkinje cells in which low cell numbers will make it more difficult to detect reliable changes in gene expression. We chose to omit downsampling nuclei to preserve power. Results were compared to a subset downsampled dataset for select clusters with larger nuclei number, and results were found to be similar both in the pattern of differentially regulated genes as well as the specific genes present in the data set. To ascertain the effects of age and PMI, differential expression analysis was performed using FindMarkers function in Seurat (MAST test, padj < 0.05, logfc.threshold = 0.25, latent.vars = "age" or "PMI"). We used covariate "age" and "PMI" in MAST separately. To generate a set of FMRP target genes in humans, FMRP targets that were functionally validated in human cell types were combined (2-4), and DiVenn (https://divenn.tch.harvard.edu) was used to visualize expression of this FMRP network in cellular subsets (5). Gene ontology/ enrichment analysis was conducted with clusterProfiler (6, 7) with statistical significance testing using padj (Benjamin Hochberg) < 0.05. Transcriptional regulators were identified using the RCisTarget R package (8) that utilizes the cisTarget database of gene regulators and identifies enriched transcription factor binding sites +/- 10kb for all genes. Input gene lists were taken from differential expression analysis results as described above from the premutation vs control cortical microglia and cerebellar Bergmann glia lists. A normalized enrichment score cutoff greater than 3 was used to identify significantly enriched motifs.

For pseudotime analysis, Monocle3 (9, 10) was used to recluster oligodendrocyte clusters, rescaling integrated data and regressing out the top 9 most differentially expressed genes. Spatial autocorrelation for gene expression changes across pseudotime were detected with Moran's I test (full and single branches separately). Genes were selected with q-value < 0.05 and Moran's statistic > 0.1 . To fit NB-GAM models and fitting smoothers in a condition-specific manner (tradeSeq), all genes were used for normalization but the model fitting is done only to the genes selected and the genes of interest (oligodendrocyte markers). Differential gene expression across

pseudotime between conditions (fold change greater than 2) was conducted with the Wald test, testing whether conditions are the significant variable in a model of expression along the trajectory (pseudotime). Mitochondrial genes were omitted from visualization of results given potential confounding effects.

Supplemental References

1. R. Satija, J. A. Farrell, D. Gennert, A. F. Schier, A. Regev (2015) Spatial reconstruction of single-cell gene expression data. in *Nature Biotechnology*.
2. S. S. Tran *et al.*, Widespread RNA editing dysregulation in brains from autistic individuals. *Nat Neurosci* **22**, 25-36 (2019).
3. M. Ascano, Jr. *et al.*, FMRP targets distinct mRNA sequence elements to regulate protein expression. *Nature* **492**, 382-386 (2012).
4. M. Li *et al.*, Identification of FMR1-regulated molecular networks in human neurodevelopment. *Genome Res* **30**, 361-374 (2020).
5. L. Sun *et al.*, DiVenn: An Interactive and Integrated Web-Based Visualization Tool for Comparing Gene Lists. *Front Genet* **10**, 421 (2019).
6. T. Wu *et al.*, clusterProfiler 4.0: A universal enrichment tool for interpreting omics data. *Innovation (Camb)* **2**, 100141 (2021).
7. G. Yu, L. G. Wang, Y. Han, Q. Y. He, clusterProfiler: an R package for comparing biological themes among gene clusters. *OMICS* **16**, 284-287 (2012).
8. S. Aibar *et al.*, SCENIC: single-cell regulatory network inference and clustering. *Nat Methods* **14**, 1083-1086 (2017).
9. C. Trapnell *et al.*, The dynamics and regulators of cell fate decisions are revealed by pseudotemporal ordering of single cells. *Nat Biotechnol* **32**, 381-386 (2014).
10. X. Qiu *et al.*, Reversed graph embedding resolves complex single-cell trajectories. *Nat Methods* **14**, 979-982 (2017).
